# Solid-Phase Methodology for Synthesis of *O*-Alkylated Aromatic Oligoamide Inhibitors of α-Helix-Mediated Protein–Protein Interactions

**DOI:** 10.1002/chem.201204098

**Published:** 2013-03-18

**Authors:** Natasha S Murphy, Panchami Prabhakaran, Valeria Azzarito, Jeffrey P Plante, Michaele J Hardie, Colin A Kilner, Stuart L Warriner, Andrew J Wilson

**Affiliations:** [a] School of Chemistry, University of LeedsWoodhouse Lane, Leeds, LS29 JT (UK), Fax: (+44) 1133431409 E-mail: A.J.Wilson@leeds.ac.uk; [b] Astbury Centre for Structural Molecular Biology, University of LeedsWoodhouse Lane, Leeds, LS2 9JT (UK)

**Keywords:** amides, foldamers, helical structures, protein–protein interactions, solid-phase synthesis

Dedicated to Prof. Andrew D. Hamilton on the occasion of his 60th birthday

A major effort in modern bio-organic chemistry focuses on the design, synthesis and structural characterisation of foldamers:^1^ non-natural oligomers that adopt well-defined secondary, tertiary and quaternary structures.^2^–^5^ One ultimate objective of such studies is to recapitulate the functional behaviour of biomacromolecules.^6^ Particular emphasis has been placed on inhibitors^7^–^12^ of α-helix-mediated^13^ protein–protein interactions^14^—an endeavour that in its own right represents a major challenge.^15^, ^16^ The development of synthetic methodologies that allow access to small-to-medium sized libraries of foldamers incorporating diverse side chains, represents the cornerstone upon which such studies are pursued. In this regard, it is noteworthy that the most robust methodology exists for peptoids,^17^ β-peptides^18^ and more recently oligoureas;^19^ templates that have seen the most significant use in a biological context.^7^, ^20^ We^21^–^24^ and others^25^–^29^ have recently reported on the use of aromatic oligoamides^5^ as potential α-helix mimetics.^30^, ^31^ Rather than topographical mimicry of the α-helix (as is the case for β,^32^ α/β^7^, ^9^, ^12^ and other foldamers^8^), these compounds mimic an α-helix by presenting key side chains from a rod-like template in a spatial orientation that matches that of the α-helix (Figure 1).^33^ Although solution methods for assembly of very large^34^ and long aromatic oligoamides^35^ have been described, a significant advance in this area would be the ready availability of solid-phase methods tolerant to a diverse array of side chains; this would facilitate library generation and ease of purification. Other than our own preliminary report on *N*-alkylated aromatic oligoamides,^22^ only a limited number of reports have been described on the synthesis of benzanilides^36^, ^37^ and related aromatic oligoamides^38^, ^39^ that meet the criteria outlined above. Herein, we describe such a method that can be used for synthesis of 3-*O*-alkylated aromatic oligobenzamides. Using microwave irradiation, trimers can be assembled on a solid support in 2.5 h in sufficient purity for screening purposes. The methodology is tolerant to a large and diverse collection of monomers and amenable to synthesis of significantly longer oligomers. The approach and our observations in developing it should have general applicability for synthesis of aromatic oligoamide foldamers.

**Figure 1:**
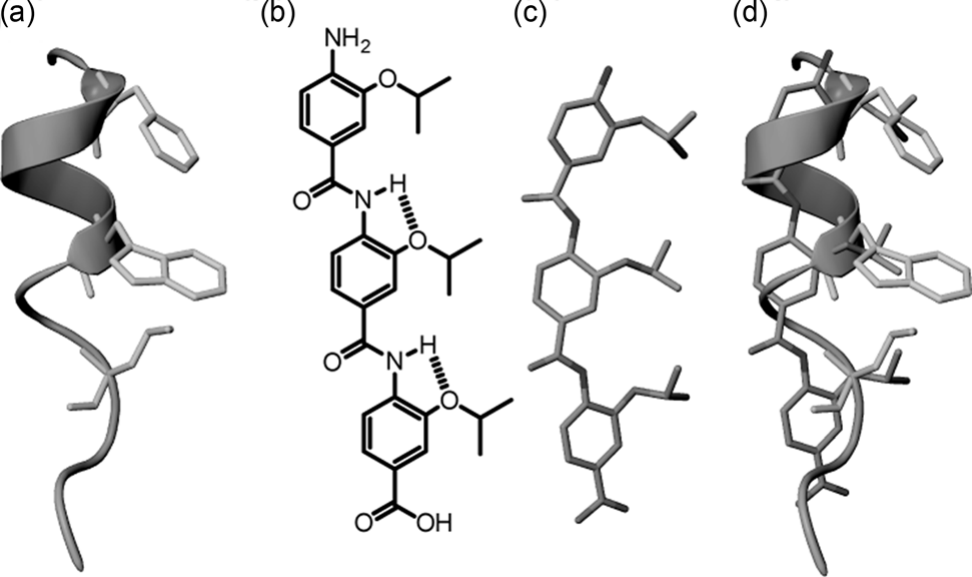
a) α-Helix (taken from protein database ID: 1YCR) with *i*, *i*+4 and *i*+7 side chains highlighted; b) chemical structure of 3-*O*-alkylated oligoamide helix mimetic c) energy minimised structure of a helix mimetic with R^3^=R^2^=R^3^=*i*Pr; d) idealized α-helix superimposed onto minimised aromatic oligoamide.

In developing our approach we sought to avoid implementation of novel protecting group chemistries and constrained ourselves to use of Fmoc (Fmoc=fluorenylmethyoxycarbonyl) as a semi-permanent protecting group and permanent acid labile protecting groups on the side chains. On this basis a four-step synthesis of a broad array of monomers **1 a**–**r** was developed (Scheme 1) exploiting either alkylation of the intermediate phenol at the diversification point using alkyl halides or alcohols under Mitsunobu conditions (i.e., **2** to **3** in Scheme 1). As is shown, a full array of peptide based side chains covering the entirety of functionality found in native peptide side chains is accessible (with the exception of cysteine, arginine and histidine), whilst several non-natural side chains and chiral side chains can also be incorporated. There are several noteworthy points as follows: 1) for benzylic side chains (e.g., **3 e**–**k**) it was necessary to use tin(II) chloride for nitro group reduction (**3** to **4** in Scheme 1) as opposed to palladium on charcoal, so as to avoid cleavage of the side chain, 2) for the hydrolysis step (**4** to **5** in Scheme 1), care was be required to avoid cleavage of the side chain, requiring use of lithium hydroxide and mild conditions (e.g., room temperature). Cleavage of the side chain by elimination of the phenol occured under forcing conditions—a feature that prevented us from obtaining a monomer mimicking histidine. Additionally, for side chains possessing an electron-withdrawing group γ to the phenol, E1CB was promoted (not shown). Finally, for the *tert*-butyl ester side chain **1 m** we observed deprotection of the *tert*-butyl group using sodium hydroxide presumably via a ketene intermediate. We also synthesised an Fmoc-protected monomer mimicking glycine; our design positions the phenolic oxygen as the atom mimicking the α position of the amino acid within the helix and therefore this represents a poor mimic of glycine and would require protection during synthesis. We therefore protected commercially available 3-methyl-4-aminobenzoic acid with Fmoc to furnish the glycine mimic **1 s**.

**Scheme 1:**
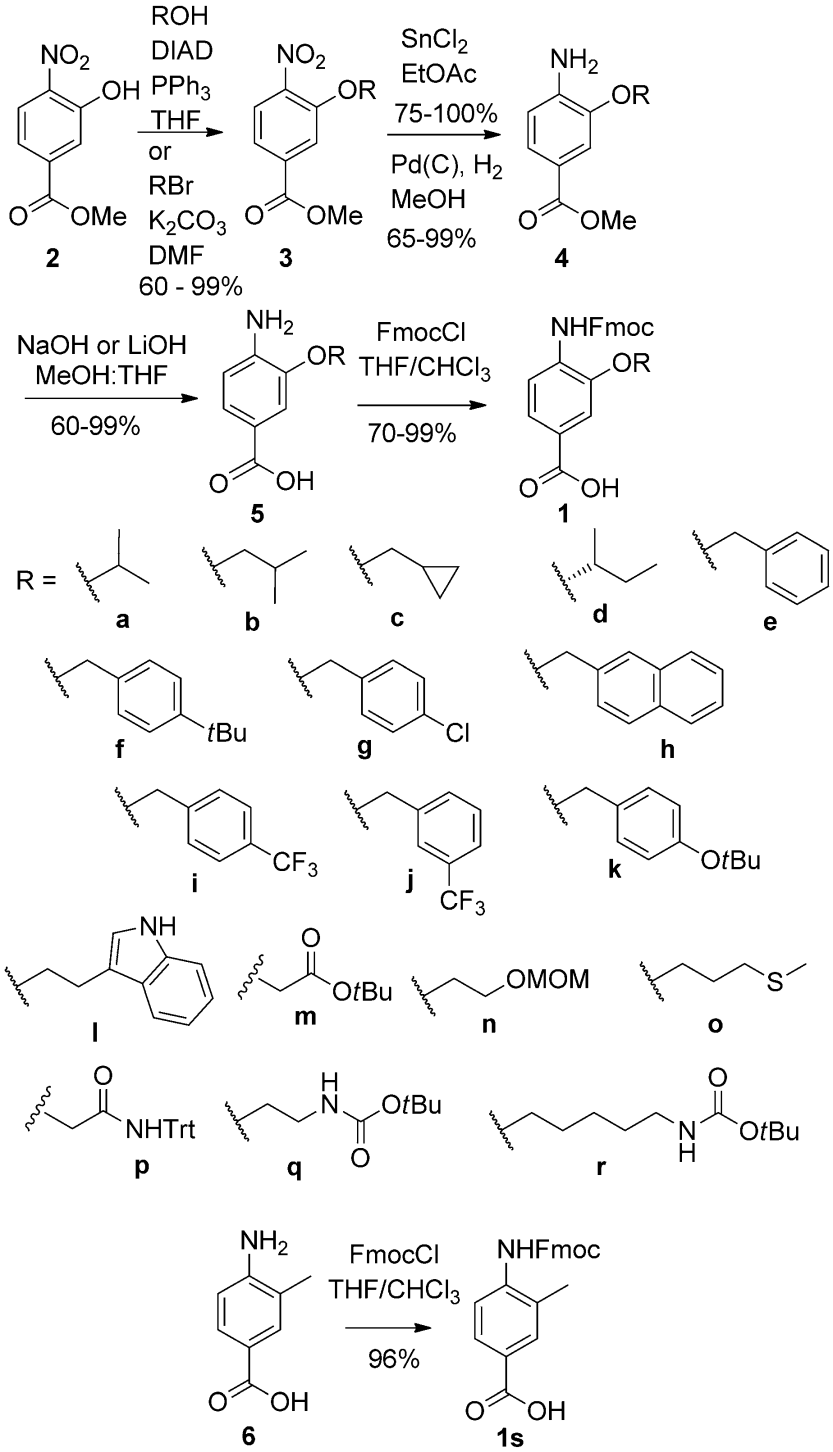
Synthesis of Fmoc-protected monomers for SPS.

A general outline of the solid-phase synthesis (SPS) method is illustrated in Scheme 2. In terms of developing this methodology, the amide bond forming reaction is challenging as the substrate is a deactivated aniline. We selected acid labile Wang resin for these studies and achieved resin loading using thionyl chloride or Ghosez’s reagent either directly to the resin or to glycine loaded resin. We attempted a series of screening experiments using the isopropyl monomer **1 a**, chloroform as solvent and microwave assistance (using a CEM peptide synthesiser) to identify suitable coupling regents. From our screening experiments, only activating agents that form acid chlorides proved successful (i.e., thionyl chloride and Ghosez’s reagent). This was not entirely surprising given that our prior studies,^21^, ^22^, ^24^ in the absence of microwave, indicated to us that strongly activated acids (e.g., acid chlorides) would be necessary to mediate amide bond formation. We then proceeded to develop an optimised method by attempting oligomer synthesis and broadening the monomer set. Unfortunately, the majority of monomers were found to be poorly soluble in chloroform, so we resorted to the use of DMF. Using in situ formation of the acid chloride from Ghosez’s reagent and microwave irradiation, no anilide formation was observed. Similarly, with pre-activation or isolation of the acid chloride followed by microwave assistance, we were unable to effect the anilide formation. An explanation for these results was obtained from LC-MS analysis of the reaction mixture, which revealed capping of the immobilised aniline by both DMF and Ghosez’s reagent to give a stable amidine (Figure 2). This capping reaction, which is observed even when the acid chloride is used directly, indicates that the solvent reversibly reacts with the acid chloride to generate the Vilsmeier intermediate, which can then cap the aniline. We did not observe this behaviour for synthesis of *N*-alkylated aromatic oligoamides—one explanation is that capping of an *N*-alkylated aniline results in an unstable intermediate, which cannot lose a proton to form the amidine (Figure 2). With these results in hand we performed a solvent screen to identify more polar solvents, which would not lead to such side reactions.

**Scheme 2:**
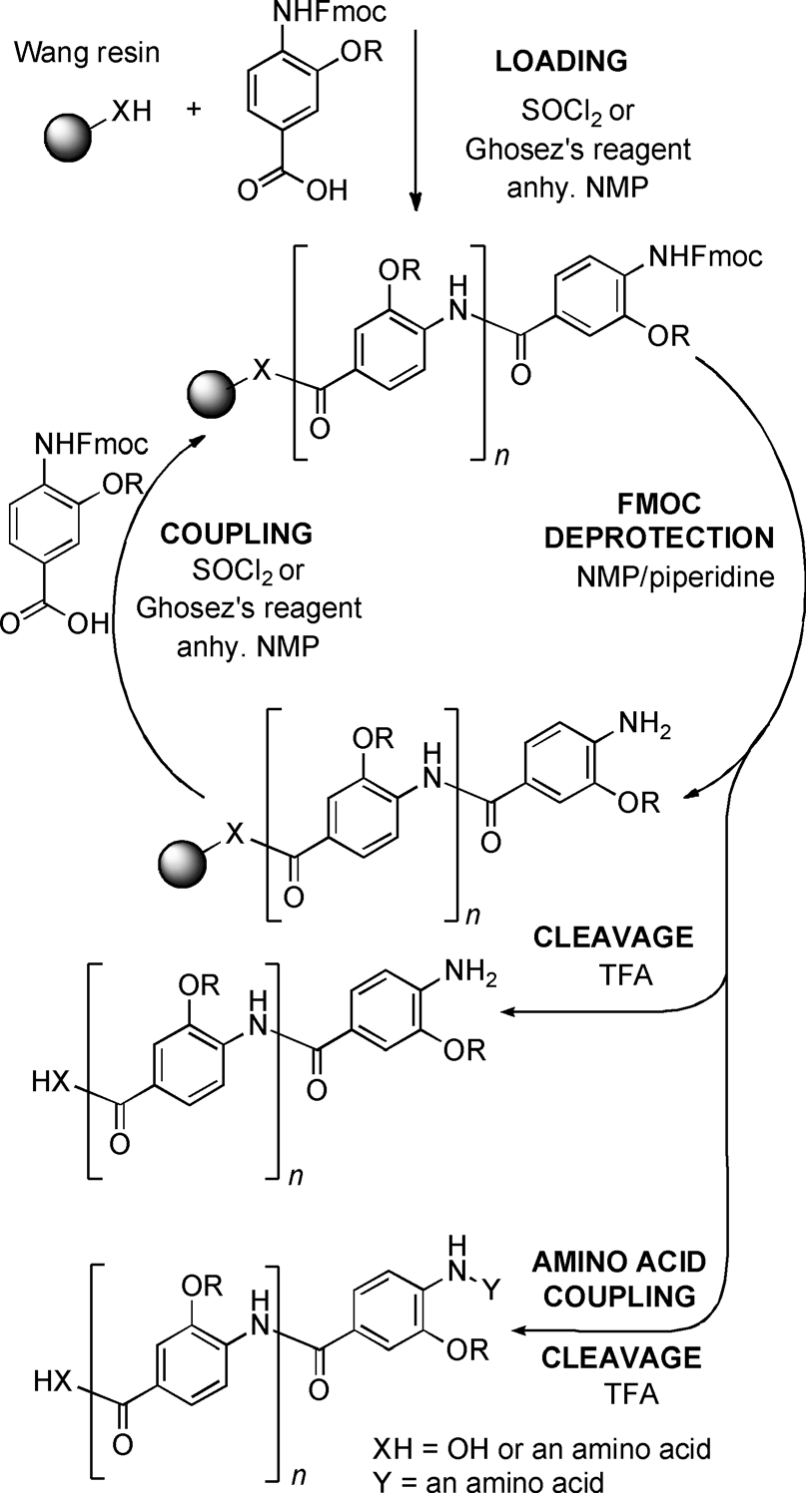
Solid-phase synthesis protocol.

**Figure 2:**
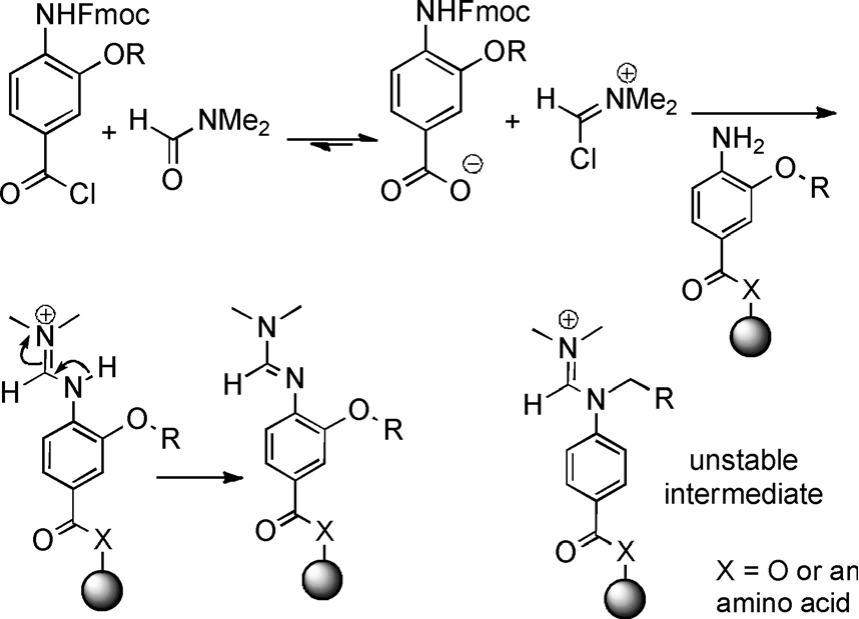
Mechanism for capping of anilines during SPS via Vilsmeier intermediates.

From our solvent screen we identified *N*-methylpyrrolidinone (NMP) as a suitable solvent with which to perform solid-phase coupling to give the aromatic benzamides. After further screening and optimisation we established that direct use of the acid chlorides obtained from thionyl chloride or pre-activation using Ghosez’s reagent prior to coupling in the microwave synthesiser could effect coupling in high yield (Scheme 2). The use of acid chlorides was preferable for the majority of alkyl/aryl Fmoc-protected monomers **1** as these could be precipitated and stored for at least one month with no decomposition. For the more highly functionalised monomers that tended not to precipitate upon reaction with thinoyl chloride it was preferable to use the in situ method (these highly functionalised monomers tended to be less stable as acid chlorides). We found that for direct addition of acid chlorides, a single cycle of coupling at 50 °C for 30 min in the absence of base was sufficient to achieve high conversion, however, for longer oligomers we used double couplings. For Fmoc removal no special optimisations were required and 20 % piperidine in NMP was sufficient (Scheme 2). Care was required with the global deprotection reaction, which we performed off-line from the synthesiser; certain side chains (see below) were found to be susceptible to cleavage by elimination with the indole side chain a notable example, thus this stage of the procedure requires careful monitoring.

With these observations and optimisations established, we demonstrated the versatility of the method by synthesising a sufficient number of trimers **7**–**24** (Table 1) so as to demonstrate that each monomer in the set could couple and be coupled to. In addition we also illustrated that amino acids (other than glycine could be appended to the C-terminus **27** (through use of different amino acid loaded Wang resins) and to the N-terminus. The only problematic monomer was **1 k** with the resulting oligomer undergoing cleavage of the benzylic phenol under the standard deprotection conditions required to cleave the phenolic *tert*-butyl protecting group. In addition, whilst we observed reasonable coupling with monomer **1 l**, we were unable to isolate and characterise the resulting trimer (**18**). Finally we also illustrated the versatility and power of the method through synthesis of longer oligomers **25**, **26** and **28** (up to a hexamer). This foldamer was obtained in 10 h using double couplings and the NMR spectrum is given in Figure 3 a for the product obtained directly from the resin. This spectrum is typical of the spectral data that is obtained immediately following resin cleavage and indicates that the oligomers are obtained in sufficient purity for preliminary screening. In several instances, this was not the case, however, cleaner material could be obtained by preperative HPLC.

**Table 1:** Oligomers synthesised.^[a]^

Trimer	R^1^	R^2^	R^3^	Final purity [%]	Yield [%]	
					Precipitate	HPLC
**7**	**a**	**a**	**a**	95	73	–
**8**	**e**	**e**	**e**	95	92	–
**9**	**e**	**h**	**a**	90	71	–
**10**	**a**	**h**	**e**	90	82	–
**11**	**e**	**i**	**a**	99	86	–
**12**	**g**	**j**	**a**	99	99	–
**13**	**e**	**g**	**a**	95	78	–
**14**	**a**	**s**	**a**	99	64	32
**15**	**a**	**d**^[b]^	**a**	99	73	35
**16**	**a**	**f**^[b]^	**a**	99	79	21
**17**	**a**	**k**	**a**	N/A	N/A	N/A
**18**	**a**	**l**	**a**	N/A	N/A	N/A
**19**	**a**	**m**^[b]^	**a**	99	69	22
**20**	**a**	**n**^[b]^	**a**	99	–	19
**21**	**a**	**o**^[c]^	**a**	99	–	22
**22**	**a**	**p**	**a**	90	69	32
**23**	**a**	**q**^[a]^	**a**	99	–	28
**24**	**a**	**r**^[b]^	**a**	99	–	17

Oligomer	Sequence	Final purity [%]	Yield [%]	
			Precipitate	HPLC
**25**	**acbb**(G)	95	91	–
**26**	**acca**(G)	95	93	–
**27**	**abd**(I)	99	–	35
**28**	**aaaaaa**(G)	95	87	–

[a] For an explanation of the R groups, see Scheme 1. [b] By using in situ activation of monomer with thionyl chloride. [c] By using in situ activation of monomer with Ghosez’s reagent.

**Figure 3:**
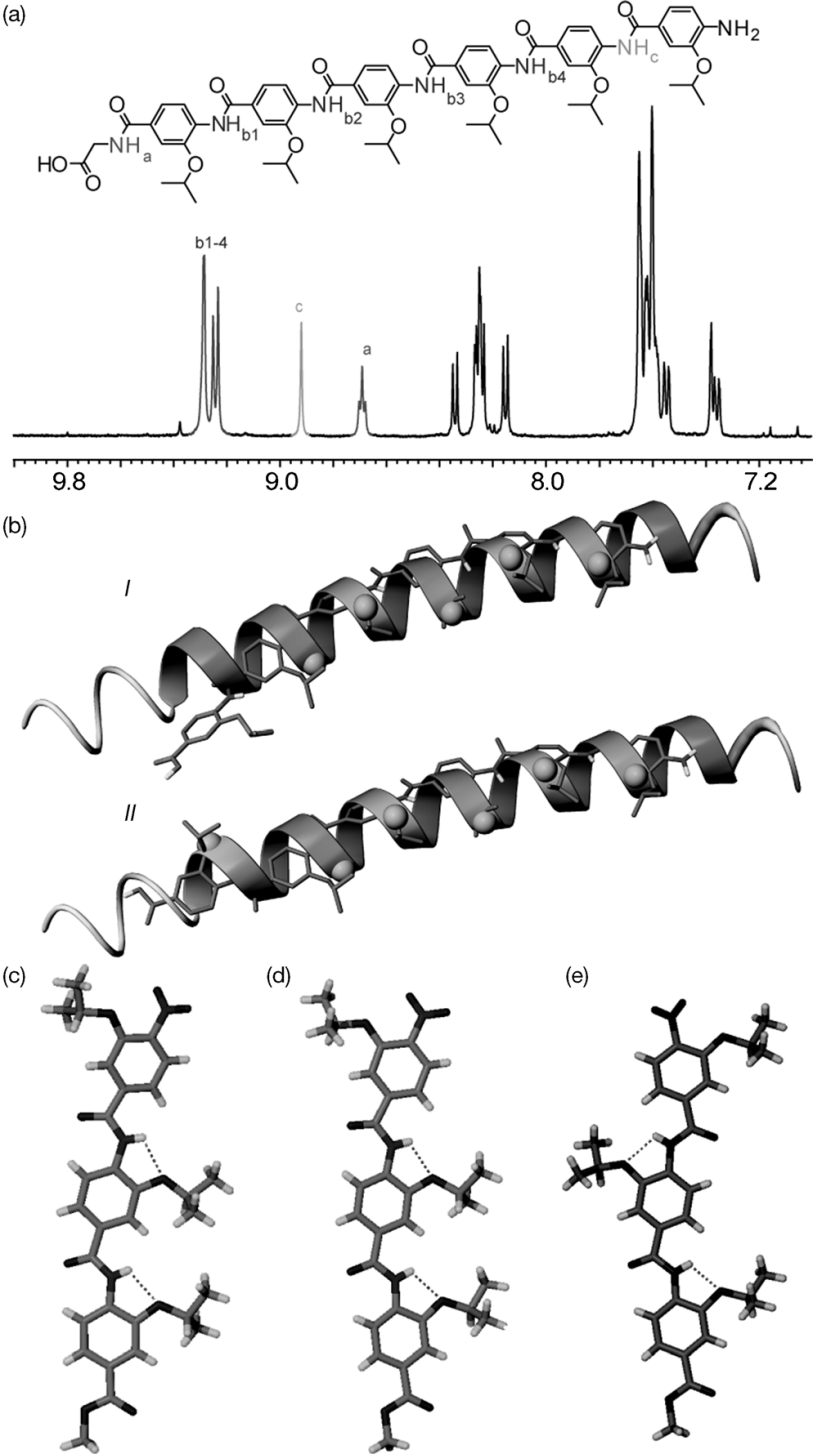
Characterisation of *O*-alkylated aromatic oligoamides a) ^1^H NMR spectrum (500 MHz, [D_6_]DMSO, 60 °C) of a hexamer b) molecular model of a hexamer with all side chains on one face together with an overlay onto an α-helix illustrating mimicry of more extended surfaces c) solid-state structure I of trimer **29** (from THF) d) solid-state structure II of trimer **29** (from chloroform/ethanol) e) solid-state structure III of trimer **29** (from chloroform/cyclohexane).

To illustrate the potential for longer oligomers to act as mimics of extended helices, we performed molecular modelling on the hexamer **28** as is illustrated in Figure 3 b (see the Supporting Information for more details). With all side chains located on one face, hexamer **28** can project side chains in such an orientation so as to mimic five consecutive side chains along an α-helical surface, whilst rotation around the terminal Ar–CO bond permits the hexamer **28** to mimic a sixth chain. This demonstrates that such oligomers could find use in the inhibition of more extended α-helix mediated protein–protein interactions. Finally we obtained several crystal structures of a representative trimer **29** (described previously)^21^, ^24^ comprising isopropyl monomers and with a C-terminal methyl ester and N-terminal nitro group (Figure 3 c–e). These exemplify the salient points of our previously published analysis of the conformational preference of these oligomers,^21^, ^23^, ^40^ that is, they adopt a rod-like conformation with free rotation around the Ar–CO axes and rotation around the Ar–NH axes restricted through S(5) intramolecular hydrogen bonding. Critically, side chains adopt both *syn* and *anti* orientations with respect to one another, whilst variations along the backbone permit the side chains to project in subtly different orientations.^23^

In conclusion, we have developed a method for synthesis of aromatic oligoamides containing a diverse array of natural and non-natural amino acid side chains using a microwave-assisted automated peptide synthesiser. A four-step monomer synthesis allows generation of Fmoc-protected building blocks for SPS with trimers accessible in 2.5 h in sufficient purity for screening. These foldamers represent good templates to act as mimetics of the α-helix and hence as inhibitors of protein–protein interactions. Our method represents a useful tool with which to obtain protein–protein interaction inhibitors by sequence based design^9^ and for library generation to screen against unknown targets. Our own future efforts in this area will describe detailed studies on aromatic oligoamide helix mimetics targeted against a broad range of protein–protein interactions.

## Experimental Section

**General procedure for oligomer formation**—**single coupling**: Fmoc-protected pre-loaded Wang resin (127 mg, 0.1 mmol, 1 equiv) was loaded onto a CEM microwave peptide synthesiser after being swelled for a total of 30 min in NMP and CH_2_Cl_2_ solutions. A series of washes (3×NMP), deprotection (2×20 % Piperidine/NMP, total of 3.5 min at 75 °C) and further washes (5×NMP) prepared the resin for coupling. Fmoc protected acyl chloride of **1 a**–**s** (0.4 mmol, 4 equiv) obtained by pre-activation or prepared separately was dissolved in NMP (2.5 mL), delivered to the reaction vessel and submitted to microwave irradiation at 50 °C for 30 min. A final series of filtered washes of the reaction vessel (3×NMP) finishes a coupling cycle.

## Supplementary material

As a service to our authors and readers, this journal provides supporting information supplied by the authors. Such materials are peer reviewed and may be re-organized for online delivery, but are not copy-edited or typeset. Technical support issues arising from supporting information (other than missing files) should be addressed to the authors.
