# Supplementary material for: Solid-Phase Methodology for Synthesis of O-Alkylated Aromatic Oligoamide Inhibitors of α-Helix-Mediated Protein–Protein Interactions
Source: Chemistry. 2013 Mar 18;19(18):5546–50. doi: 10.1002/chem.201204098 (PMC3743211; doi:10.1002/chem.201204098)
Supplement: Supplementary file 1 [file chem0019-5546-SD1.pdf]

CHEMISTRY
A EUROPEAN JOURNAL

Supporting Information

© Copyright Wiley-VCH Verlag GmbH & Co. KGaA, 69451 Weinheim, 2013

Solid-Phase Methodology for Synthesis of *O*-Alkylated Aromatic Oligoamide Inhibitors of α-Helix-Mediated Protein–Protein Interactions

Natasha S. Murphy,[a, b] Panchami Prabhakaran,[a, b] Valeria Azzarito,[a, b] Jeffrey P. Plante,[a] Michaele J. Hardie,[a] Colin A. Kilner,[a] Stuart L. Warriner,[a, b] and Andrew J. Wilson*[a, b]

chem_201204098_sm_miscellaneous_information.pdf

Contents

General Experimental Points 2
General Procedures for Monomer Syntheses 3
General Procedures for SPS 4
Characterization of Monomers 6
Characterization of Oligomers 25
Molecular Modelling 31
Crystallographic Details 32
LC-MS Data 34

General Experimental Points

All reagents were obtained from commercial sources and used without further purification unless otherwise stated. All solvents used were HPLC grade. Dry solvents were bought from Aldrich. Analytical TLC was performed using 0.2 mm silica gel 60 F_{254} pre-coated aluminium sheets (Merck) and visualised using UV irradiation or, in the case of amine intermediates, by staining with a ninhydrin solution. Flash column chromatography was carried out on silica gel 60 (35 to 70 micron particles, FluoroChem). Solvent ratios are described where appropriate. Solvents were removed under reduced pressure using a rotary evaporator at diaphragm pump pressure. Samples were freed of remaining traces of solvents under high vacuum. ^{1}H and ^{13}C NMR spectra were measured on a Bruker DPX300 or a Bruker Avance 500 spectrometer using an internal deuterium lock. Chemical shifts are reported in parts per million (ppm) downfield from TMS in d units and coupling constants are given in hertz (Hz). Coupling constants are reported to the nearest 0.1 Hz. TMS is defined as 0 ppm for ^{1}H NMR spectra and the centre line of the triplet of $CDCl_3$ was defined as 77.10 ppm for ^{13}C NMR spectra. When describing ^{1}H NMR data the following abbreviations are used; s = singlet, d = doublet, t = triplet, q = quartet, m = multiple, br. = broad, app. = apparent. Melting points were determined using a *Reichert Austria melting point microscope* and are uncorrected. Microanalyses were obtained on a Carlo Erba Elemental Analyser MOD 1106 instrument, found composition is reported to the nearest 0.05%. Infrared spectra were recorded on a Perkin-Elmer FTIR spectrometer and samples analysed as solids (unless otherwise stated). Mass spectra (HRMS) were recorded in-house using a

Micromass GCT Premier, using electron impact ionisation (EI) or a Bruker Daltonics micrOTOF, using electron spray ionisation (ES). LC-MS experiments were run on a Bruker Daltonics esquire™ series spectrometer, samples ionised by electrospray and analysed by a quadrupole ion trap mass spectrometer. All experiments were run through a C18 column on an acetonitrile/water gradient (typically 0-100% acetonitrile over 3 minutes). Intermediates **3a, 3e, 3h, 4a** and **4e** have previously been described.[1-3]

Assignment of the compounds is as follows; naming proceeds from N to C terminus where each hydroxyl amino benzoic acid (HABA) residue is assigned a number with respect to its position on the chain. Side-chain assignment follows a peptide nomenclature pattern in which the carbon attached to the alkoxy oxygen is assigned as Ca. Examples of oligomers are given below however monomer intermediates follow the same assignment.

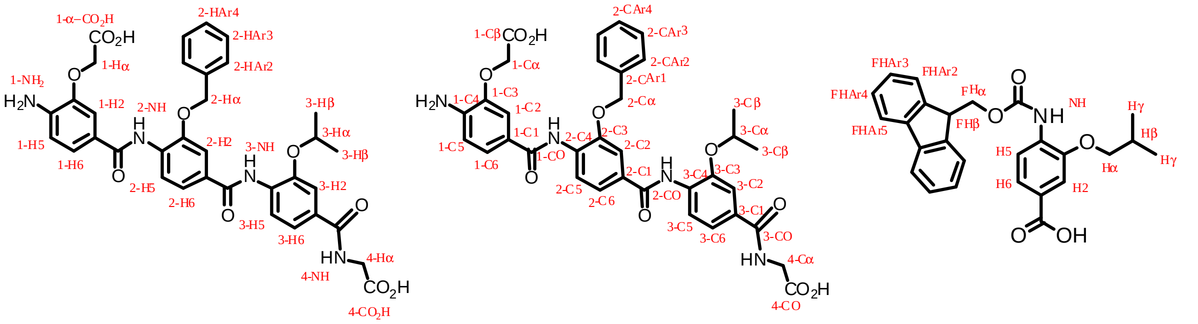

General Procedures for Monomer Syntheses

Procedure A (RBr Alkylation)

To a stirred solution of methyl-3-hydroxy-4-nitrobenzoate **2** (1 eq.) and potassium carbonate (3 eq.) in dimethylformamide (20 mL / g) is added RBr (1.2 eq.) and the resulting mixture stirred at 50°C overnight under a nitrogen atmosphere. The resultant red liquid is allowed to cool and poured into water (40 mL / g) and extracted with ethyl acetate. The combined organic fractions are thoroughly washed with water and further washed by brine, dried (magnesium sulfate), filtered and evaporated to dryness.

Procedure B (*Mitsunobu*)

A stirred solution containing methyl-3-hydroxy-4-nitrobenzoate **2** (1 eq.), ROH (1.1 eq.) and triphenylphosphine (1.5 eq.) in anhydrous tetrahydrofuran (30 mL / g) is cooled to 0°C. Diisopropyl azodicarboxylate (1.5 eq.) is added and the resulting solution allowed to warm to room temperature and left stirring overnight under a nitrogen atmosphere. Organic solvents are removed under reduced pressure and the product is purified *via* column chromatography.

Procedure C (Tin Reduction)

To a stirred solution containing either i) nitro/ester or ii) nitro/acid (1 eq.) in ethyl acetate (20 mL / g) tin(II) chloride dihydrate (6 eq.) is added and the resulting mixture stirred at 50°C overnight, under a nitrogen atmosphere (with a calcium chloride drying tube attached). On completion, the reaction

mixture is allowed to cool and poured over ice. The pH is made slightly basic (~pH 8) by addition of a saturated sodium bicarbonate solution and the resulting basic mixture is allowed to stir for an hour. The aqueous mixture is extracted with ethyl acetate and the combined organic fractions washed thoroughly with brine, dried (magnesium sulfate), filtered and evaporated to dryness

Procedure D (Hydrogenation)

A solution containing either i) nitro/ester or ii) nitro/acid (1 eq.) in methanol (20 mL / g) and palladium on carbon (10 wt%) is evacuated and flushed with nitrogen (3 times) and left under vacuum. Hydrogen is drawn into and the flask and the reaction is left stirring at room temperature overnight. On completion, the reaction mixture is filtered through a celite pad and evaporated to dryness.

Procedure E(Cobalt assisted reduction)

To a solution containing either i) nitro/ester or ii) nitro/acid (1 eq.) in methanol, cobalt (II) chloride hexahydrate (3 eq.) is added and allowed to dissolve. Sodium borohydride (6 eq.) is added slowly and allowed to stir for 30 minutes. A further portion of cobalt (II) chloride hexahydrate (3 eq.) and sodium borohydride (6 eq.) is added as above. The solution is filtered through a celite pad which is washed with dichloromethane and the filtrate thoroughly washed with 1M hydrochloric acid to remove the metal. The organic solvents are washed with brine, dried (magnesium sulfate), filtered and evaporated to dryness.

Procedure F (NaOH Saponification)

To a solution containing either i) amine/ester or ii) nitro/ester (1 eq.) in a 1:1 mixture of methanol: tetrahydrofuran (25 mL / g), a 10% sodium hydroxide solution (5 mL / g) is added and the resulting mixture is allowed to stir at RT overnight. Addition of further portions of the hydroxide solution may be necessary. The organic solvents are removed under reduced pressure and water is added to dissolve the solid. The resulting solution is extracted with dichloromethane (unreacted starting material) and the aqueous layer acidified *via* the addition of hydrochloric acid (conc) to pH 4. The resulting precipitate is extracted into dichloromethane and the combined organic extracts are washed with water and further washed with brine, dried (magnesium sulfate), filtered and evaporated to dryness.

Procedure G (LiOH Saponification)

To a solution containing either i) amine/ester or ii) nitro/ester (1 eq.) in a 1:1 mixture of tetrahydrofuran / water (25 mL / g), a saturated lithium hydroxide solution (1 eq.) is added and the resulting mixture is allowed to stir at RT overnight. The organic solvent is removed under reduced pressure and an additional amount of water is added. The resulting solution is extracted with dichloromethane (unreacted starting material) and the aqueous layer acidified *via* the addition of 1M potassium bisulfate solution to pH 4. The resulting precipitate is extracted into dichloromethane and the combined organic extracts are washed with water and further washed with brine, dried (magnesium sulfate), filtered and evaporated to dryness.

Procedure H (Fmoc protection)

A solution of amine/acid (1 eq.) in anhydrous tetrahydrofuran (20 mL / g) is held at a reflux under a nitrogen atmosphere. A solution of fluorenylmethyloxycarbonyl chloride (1.5 eq.) in anhydrous tetrahydrofuran (10 mL / g) is then added dropwise and the resulting solution is stirred at reflux overnight. The reaction mixture is cooled to ambient temperature, concentrated and the resulting precipitate collected *via* filtration.

Procedure I (Fmoc protection)

A solution of amine/acid (1 eq.) and sodium bicarbonate (3 eq.) in anhydrous tetrahydrofuran (20 mL / g) is held at a reflux under a nitrogen atmosphere. A solution of fluorenylmethyloxycarbonyl chloride (1.5 eq.) in anhydrous tetrahydrofuran (10 mL / g) is then added dropwise and the resulting solution is stirred at reflux overnight. Sodium bicarbonate is removed *via* hot filtration and the reaction mixture is allowed to cool to room temperature, concentrated under reduced pressure and the resulting precipitate collected *via* filtration.

General Procedures for SPS

Acyl Chloride Formation – Method A To a stirred solution of an Fmoc protected building block in anhydrous dichloromethane (20 mL / g), thionyl chloride (10 eq) is added and the resulting mixture refluxed overnight. The organic solvents and thionyl chloride are removed under reduced pressure and the resulting solid re-dissolved in chloroform. Hexane is added to precipitate the acyl chloride which is collected *via* filtration and stored under an inert atmosphere.

Acyl Chloride Preactivation – Method B To a solution containing Fmoc protected monomers (1 equiv.) functionalised with acid sensitive protecting groups in NMP (2.5 mL), 0.9 eq. of Ghosez's reagent is added. The resulting mixture is stored under an inert atmosphere for 3 hours at 50 ºC before the addition to the resin and microwave treatment.

Acyl Chloride *In Situ* Formation- Method C To a solution containing Fmoc protected monomers (1 equiv.) in NMP (2.5 mL), 1 eq. of thionyl chloride is added immediately before addition to the resin and microwave treatment

General Points for Solid Phase Synthesis

Fmoc-Gly-Wang resin (0.79 mmol/g, 100-200 mesh; carrier: polystyrene, crosslinked with 1% DVB), Fmoc-Ile-Wang resin (0.59 mmol/g, 100-200 mesh; carrier: polystyrene, crosslinked with 1% DVB) was purchased from Merck. All solvents used were HPLC grade. Anhydrous *N*-methyl-2-pyrrolidone was purchased from Alfa Aeser and stored in a schlenk tube on molecular sieves under a nitrogen atmosphere. Acyl chlorides were synthesised as in Method A for acyl chloride formation and stored under an inert atmosphere. 1-Chloro-*N*, *N*, 2-trimethyl-1-propenylamine (Ghosez's reagent) was purchased from Sigma-Aldrich. Oligomer formation was carried out on a CEM Liberty automated microwave peptide synthesiser. The volume of the reaction mixture in the reaction vessel was 2.5 mL. Manual SPS was carried out in 1.5 mL 'Extract-Clean' polypropylene reservoirs fitted with 20 mm polyethylene frits, both available from Alltech.

General Procedure for Oligomer Formation – Single Coupling Fmoc protected pre-loaded Wang resin (127 mg, 0.1 mmol, 1 equiv.) is loaded onto a CEM™ microwave peptide synthesiser after being swelled for a total of 30 minutes in NMP and DCM solutions. A series of washes (3 x NMP), deprotection (2 x 20 % Piperidine/NMP, total of 3.5 minutes at 75 °C) and further washes (5 x NMP) prepares the resin for coupling. Fmoc protected acyl chloride **X** (0.4 mmol, 4 equiv.) obtained by pre-activation or prepared separately is dissolved in NMP (2.5 mL), delivered to the reaction vessel and submitted to microwave irradiation at 50 °C for 30 minutes. A final series of filtered washes of the reaction vessel (3 x NMP) finishes a coupling cycle.

General Procedure for Oligomer Formation – Double Coupling Fmoc protected pre-loaded Wang resin (127 mg, 0.1 mmol, 1 equiv.) is loaded onto a CEM™ microwave peptide synthesiser after being swelled for a total of 30 minutes in NMP and DCM solutions. A series of washes (3 x NMP), deprotection (2 x 20 % Piperidine/NMP, total of 3.5 minutes at 75 °C) and further washes (5 x NMP) prepares the resin for coupling. Fmoc protected acyl chloride **X** (0.2 mmol, 2 equiv.) obtained by pre-activation or prepared separately is dissolved in NMP (2.5 mL), delivered to the reaction vessel and submitted to microwave irradiation at 50 °C for 30 minutes. A second solution containing Fmoc protected acyl chloride **X** (0.2 mmol, 2 equiv.) (preactivated or isolated) in NMP (2.5 mL) is delivered to the reaction vessel and submitted to microwave power at 50 °C for 30 minutes. A final series of filtered washes of the reaction vessel (3 x NMP) finishes a coupling cycle.

General Procedure for Cleavage After the required number of cycles, a final Fmoc deprotection is carried out and then the resin is removed from the synthesiser and transferred to a reservoir for manual cleavage. The resin is washed with dichloromethane (10 x 1 mL) and cleaved with a 1.5 mL cleavage cocktail consisting of TFA: DCM: TIPS with varying ratios depending on the side chains. If no protecting groups are present, a simple 1:1 trifluoroacetic acid-dichloromethane mixture is sufficient without the need for a scavenger.

Characterization of Monomers

4-Amino-3-isopropoxybenzoic acid 5a

Procedure F; Methyl-4-amino-3-isopropyloxybenzoate **4a** (9.00 g, 43.0 mmol) in a 1:1 mixture of methanol-tetrahydrofuran (220 mL), 10% aqueous sodium hydroxide solution (50 mL). Work-up afforded the title compound (7.56 g, 38.7 mmol, 90%) as a colourless amorphous powder; (Found C, 61.30; H, 6.75; N, 7.01%. $C_{10}H_{13}NO_3$ requires C, 61.53; H, 6.75; N, 7.18%); d_H (300 MHz, $CDCl_3$) 1.36 (6H, d, *J* = 6.8, Hß), 3.85 (2H, br. s, N<u>H</u>$_2$), 4.63 (1H, sept, *J* = 6.1, Ha), 6.68 (1H, d, *J* = 8.2, H5), 7.53 (1H, s, H2), 7.61 (1H, d, *J* = 8.2, H6); d_C (75MHz, $CDCl_3$); 21.3, 70.9, 113.2, 114.5, 118.4, 124.2, 124.4, 144.1, 169.6; $?_{max}/cm^{-1}$ (solid state) = 3335, 2520, 1769, 1659, 1577, 1443, 1262, 1111, 976; ESI-MS found *m/z* 196.09 $[M+H]^+$;

4-((((9H-fluoren-9-yl)methoxy)carbonyl)amino)-3-isopropoxybenzoic acid 1a

Procedure H; 4-Amino-3-isopropoxybenzoic acid **5a** (7.00 g, 35.9 mmol) in tetrahydrofuran (140 mL), fluorenylmethyloxycarbonyl chloride (13.92 g, 53.8 mmol) in tetrahydrofuran (100 mL). Work up yielded the title compound (12.28 g, 29.4 mmol, 82%) as a colourless amorphous solid; (Found C, 71.80, H, 5.65; N, 3.25%. $C_{25}H_{23}NO_5$ requires C, 71.93; H, 5.55; N, 3.36%); d_H (300 MHz, $CDCl_3$) 1.43 (6H, d, *J* = 6.9, Hß), 4.34 (1H, t, *J* = 6.9, FHß), 4.54 (2H, d, *J* = 6.9, FHa), 4.73 (1H, sept, *J* = 6.9, Ha), 7.34 (2H, t, *J* = 7.5, FHAr4), 7.43 (2H, t, *J* = 7.5, FHAr3), 7.55 (1H, s, H2), 7.60-7.75 (4H, m, H5, H6 + FHAr5), 7.80 (2H, d, *J* = 8.4, FHAr2), 8.16 (1H, s, N<u>H</u>); d_C (75MHz, $CDCl_3$); 21.9, 46.9, 66.7, 71.5, 114.3, 120.5, 125.5, 125.6, 126.3, 127.5, 128.1, 132.8, 141.1, 144.0, 147.4, 153.7, 167.3; $?_{max}/cm^{-1}$ (solid state) = 3333, 2975, 1709, 1599, 1542, 1497, 1442, 1337, 1240, 1105, 1053, 978; ESI-HRMS found *m/z* 418.1649 $[M+H]^+$, $C_{25}H_{24}NO_5$ requires 418.1654;

Methyl 3-isobutoxy-4-nitrobenzoate 3b

Procedure A; Methyl-3-hydroxy-4-nitrobenzoate **2** (10.00 g, 50.72 mmol) and potassium carbonate (2l.0 g, 151.9 mmol) in dimethylformamide (200 mL), 1-bromo-2-methylpropane (6.62 mL, 60.9 mmol). Following work-up, the resulting solid was crystallised (methanol/ hexane) to yield the title compound (9.17 g, 36.2 mmol, 71%) as pale yellow crystals; m.p. 68.5-69.0 °C (methanol/ hexane); d_H (300 MHz,$CDCl_3$) 1.06 (6H, d, *J* = 6.9, H?), 2.16 (1H, sept, *J* = 6.6, Hß), 3.93 (2H, d, *J* = 6.3, Ha), 3.96 (3H, s, CO_2<u>Me</u>), 7.66 (1H, dd, *J* = 8.4, 1.5, H6), 7.72 (1H, d, *J* = 1.5, H2), 7.81 (1H, d, *J* =8.4, H5); d_C (75MHz, $CDCl_3$) 17.5, 28.6, 51.2, 74.4, 113.8, 119.5, 123.6, 133.1, 140.8, 150.5, 163.7; $?_{max}/cm^{-1}$ (solid state) = 2955, 1726, 1609, 1524, 1237, 750; ESI-HRMS found *m/z* 276.0851 $[M+Na]^+$, $C_{12}H_{15}NNaO_5$ requires 276.0842;

Methyl 4-amino-3-isobutoxybenzoate 4b

Procedure C; Methyl 3-isobutoxy-4-nitrobenzoate **3b** (5.00 g, 19.7 mmol), tin(II) chloride dihydrate (26.73 g, 118.5 mmol) in ethyl acetate (150 mL). Following standard work-up the resulting solid was crystallised (dichloromethane/ hexane) to yield the title compound (3.36 g, 15.1 mmol, 76%) as colourless crystalline plates; m.p. 62.3-63.5 °C (dichloromethane/ hexane); (Found C, 64.30; H, 7.65; N, 6.35%. $C_{12}H_{17}NO_3$ requires C, 64.55; H, 7.67; N, 6.27%); d_H (300 MHz, $CDCl_3$) 1.05 (6H, d, *J* = 6.6, H?), 2.13 (1H, sept, *J* = 6.6, Hß), 3.82 (2H, d, *J* = 6.3, Ha), 3.86 (3H, s, CO_2<u>Me</u>), 4.24 (2H, s, N<u>H</u>$_2$), 6.67 (1H, d, *J* = 8.4, H5), 7.43 (1H, d, *J* = 1.5, H2), 7.53 (1H, dd, *J* = 8.4, 1.5, H6); d_C (75MHz, $CDCl_3$) 19.8, 28.7, 52.1, 75.1, 112.4, 113.5, 119.8, 124.3, 141.6, 146.0, 167.8; $?_{max}/cm^{-1}$ (solid state) = 3461, 3342, 2950, 1688, 1622, 1523, 1441, 1269, 1034, 766, 635; ESI-HRMS found *m/z* 224.1287 $[M+H]^+$, $C_{12}H_{18}NO_3$ requires 224.1281;

4-Amino-3-isobutoxybenzoic acid 5b

Procedure F; Methyl 4-amino-3-isobutoxybenzoate **4b** (1.80 g, 8.1 mmol) in a 1:1 mixture of methanol-tetrahydrofuran (50 mL), 10% aqueous sodium hydroxide (17 mL). Following acidification, the precipitate was filtered, dissolved in chloroform and dried with magnesium sulfate. The solution was filtered and the organic solvents removed under reduced pressure yielding a pink solid which was

crystallised (dichloromethane/ hexane) to yield the title compound (1.22 g, 5.8 mmol, 72%) as pale pink microcrystals; m.p. 118.7-119.6 °C (dichloromethane/ hexane); (Found C, 63.15; H, 7.15; N, 6.60%. $C_{11}H_{15}NO_3$ requires C, 63.14; H, 7.23; N, 6.69%); d_H (300 MHz, $CDCl_3$) 1.08 (6H, d, *J* = 6.7, H?), 2.16 (1H, sept, *J* = 6.7, Hß), 3.85 (2H, d, *J* = 6.6, Ha), 6.71 (1H, d, *J* = 8.2, H5), 7.5 (1H, d, *J* = 1.8, H2), 7.65 (1H, dd, *J* = 8.1, 1.8, H6); d_C (75MHz, $CDCl_3$) 19.4, 28.3, 74.7, 112.4, 113.0, 118.4, 124.9, 142.1, 145.5, 172.4; $?_{max}/cm^{-1}$ (solid state) = 3497, 3384, 2813, 1659, 1611, 1306, 765; ESI-HRMS found *m/z* 210.1122 $[M+H]^+$, $C_{11}H_{16}NO_3$ requires 210.1125.

4-((((9H-fluoren-9-yl)methoxy)carbonyl)amino)-3-isobutoxybenzoic acid 1b

Procedure H; 4-Amino-3-isobutoxybenzoic acid **5b** (3.00 g, 14.3 mmol) in tetrahydrofuran (100 mL), fluorenylmethyloxycarbonyl chloride (5.56 g, 21.5 mmol) in tetrahydrofuran (50 mL). Work up yielded the title compound (5.25 g, 12.2 mmol, 85%) as a colourless amorphous solid; (Found C, 72.10; H, 5.80; N, 3.15%. $C_{26}H_{25}NO_5$ requires C, 72.37; H, 5.84; N, 3.25%); d_H (300 MHz, DMSO-d_6) 1.04 (6H, d, *J* = 6.7, H?), 2.15 (1H, sept, *J* = 6.6, Hß), 3.84 (2H, d, *J* = 6.6, Ha), 4.26 (1H, t, *J* = 7.0, FHß), 4.46 (2H, d, *J* = 7.0, FHa), 7.26 (2H, t, *J* = 7.5, FHAr4), 7.36 (2H, t, *J* = 7.5, FHAr3), 7.48-7.51 (2H, m, H5 + H2), 7.56 (2H, d, *J* = 7.3, FHAr5), 7.67-7.74 (3H, m, H6 + FHAr2) 8.08 (1H, br. s, N<u>H</u>); d_C (125MHz, DMSO-d_6) 18.9, 27.5, 46.4, 66.3, 74.6, 112.3, 120.1, 120.3, 122.1, 125.1, 126.0, 127.0, 127.7, 131.3, 140.7, 143.6, 148.6, 153.3, 166.9; $?_{max}/cm^{-1}$ (solid state) = 3337, 2955, 1710, 1677, 1435, 1290, 736; ESI-HRMS found *m/z* 454.1615 $[M+Na]^+$, $C_{26}H_{25}NNaO_5$ requires 454.1625.

Methyl 3-(cyclopropylmethoxy)-4-nitrobenzoate 3c

Procedure A; Methyl-3-hydroxy-4-nitrobenzoate **2** (10.00 g, 50.7 mmol), potassium carbonate (2l.00 g, 151.9 mmol) in dimethylformamide (200 mL), (bromomethyl)cyclopropane (5.91 mL, 60.9 mmol). Following work-up the resultant yellow solid which was crystallised (ethyl acetate) to yield the title compound (10.34 g, 41.2 mmol, 81 %) as large pale yellow rectangular crystals; m.p. 93.7-95.1 °C (ethyl acetate); (Found C, 57.30; H, 5.20; N, 5.55%. $C_{12}H_{13}NO_5$ requires C, 57.37; H, 5.22; N, 5.58%); d_H (300 MHz, $CDCl_3$) 0.43 (2H, m, H?), 0.70 (2H, m, H?'), 1.34 (1H, m, Hß), 3.99 (3H, s, CO_2<u>Me</u>), 4.07 (1H, d, *J* = 6.8, Ha) 7.70 (1H, dd, *J* = 8.2, 1.5, H2), 7.74 (1H, d, *J* = 1.6, H6), 7.83 (1H, d, *J* = 8.3, H5); d_C (75MHz, $CDCl_3$) 3.74, 10.3, 53.2, 75.0, 116.4, 121.7, 125.6, 135.1, 143.2, 152.2, 165.7; $?_{max}/cm^{-1}$ (solid state) = 3111, 1726, 1607, 1522, 1307, 1247, 750; ESI-HRMS found *m/z* 274.0689 $[M+Na]^+$, $C_{12}H_{13}NaNO_5$ requires 274.0686.

Methyl 4-amino-3-(cyclopropylmethoxy)benzoate 4c

Procedure C; Methyl 3-(cyclopropylmethoxy)-4-nitrobenzoate **3c** (10.00 g, 39.8 mmol), tin(II) chloride dihydrate (53.89 g, 238.9 mmol) in ethyl acetate (150 mL). Following work-up, the resulting solid was crystallised (ethyl acetate/ hexane) to yield the title compound (5.77 g, 26.1 mmol, 65%) as colourless microcrystals; m.p. 81.5-82.4 °C (ethyl acetate/ hexane); (Found C, 65.05; H, 6.85; N, 6.35%. $C_{12}H_{15}NO_3$ requires C, 65.14; H, 6.83; N, 6.33%); d_H (300 MHz, $CDCl_3$) 0.29 (2H, m, H?), 0.56 (2H, m, H?'), 1.22 (1H, m, Hß), 3.78 (3H, s, CO_2<u>Me</u>), 3.81 (2H, d, *J* = 7.1, Ha), 4.20 (2H, br. s,

N$\underline{H_2}$), 6.59 (1H, d, *J* = 8.1, H5), 7.34 (1H, d, *J* = 1.7, H2), 7.46 (1H, dd, *J* = 8.1, 1.7, H6); d$_C$ (75MHz, $CDCl_3$) 3.6, 10.7, 52.1, 73.7, 113.0, 113.6, 119.8, 124.4, 141.8, 145.9, 167.7; ?$_{max}$/cm^{-1} (solid state) = 3491, 3355, 2998, 1682, 1614, 1296, 762; ESI-HRMS found *m/z* 222.1130 [M+H]$^+$, $C_{12}H_{16}NO_3$ requires 222.1125.

4-Amino-3-(cyclopropylmethoxy)benzoic acid 5c

Procedure F; Methyl 4-amino-3-(cyclopropylmethoxy)benzoate **4c** (4.64 g, 20.1 mmol) in a 1:1 mixture of methanol : tetrahydrofuran (120 mL), 10% aqueous sodium hydroxide (30 mL). The resulting precipitate was filtered, dissolved in chloroform and dried with magnesium sulfate. The solution was filtered and the organic solvents removed under reduced pressure yielding a beige solid which was crystallised (chloroform/ methanol/ hexane) to yield the title compound (3.65 g, 17.6 mmol, 84%) as large pale orange crystals; m.p. 154.5-155.9 °C (chloroform/ methanol/ hexane); (Found C, 63.25; H, 6.25; N, 6.65%. $C_{11}H_{13}NO_3$ requires C, 63.76; H, 6.32; N, 6.76%); d$_H$ (300 MHz, $CDCl_3$) 0.40 (2H, m, H?), 0.69 (2H, m, H?'), 1.33 (1H, m, Hß), 3.95 (2H, d, *J* = 7.0, Ha), 6.72 (1H, d, *J* = 7.3, H5), 7.50 (1H, d, *J* = 1.7, H2), 7.66 (1H, dd, *J* = 7.3, 1.7, H6); d$_C$ (75MHz, $CDCl_3$) 3.6, 10.7, 73.8, 113.3, 113.5, 118.8, 125.5, 142.7, 145.8, 172.8; ?$_{max}$/cm^{-1} (solid state) = 3501, 3383, 2900, 1666, 1614, 1305, 765; ESI-HRMS found *m/z* 208.0962 [M+H]$^+$, $C_{11}H_{14}NO_3$ requires 208.0968.

4-((((9H-fluoren-9-yl)methoxy)carbonyl)amino)-3-(cyclopropylmethoxy)benzoic acid 1c

Procedure H; 4-Amino-3-(cyclopropylmethoxy)benzoic acid **5c** (3.13 g, 15.1 mmol) in tetrahydrofuran (100 mL), fluorenylmethyloxycarbonyl chloride (5.87 g, 22.7 mmol) in chloroform (30 mL). Work up yielded the title compound (5.26 g, 12.3 mmol, 81%) as a colourless amorphous solid; (Found C, 72.55; H, 5.25; N, 2.95%. $C_{26}H_{23}NO_5$ requires C, 72.71; H, 5.40; N, 3.26%); d$_H$ (300 MHz, $CDCl_3$) 0.42 (2H, m, H?), 0.73 (2H, m, H?'), 1.37 (1H, m, Hß), 3.98 (2H, d, *J* = 7.1, Ha), 4.37 (1H, t, *J* = 6.8, FHß), 4.58 (2H, d, *J* = 6.9, FHa), 7.36 (2H, t, *J* = 7.4, FHAr4), 7.45 (2H, t, *J* = 7.4, FHAr3), 7.57 (1H, d, *J* = 1.7, H2), 7.66-7.69 (3H, m, H5 + FHAr5), 7.75 (1H, d, *J* = 8.5, H6), 7.82 (2H, d, *J* = 7.4, FHAr2); d$_C$ (75MHz, $CDCl_3$) 3.7, 10.6, 47.5, 67.8, 74.5, 113.0, 117.6, 120.5, 124.3, 124.5, 125.4, 127.6, 128.3, 133.0, 141.8, 144.1, 146.9, 153.5, 169.9; ?$_{max}$/cm^{-1} (solid state) = 3329, 2807,1707, 1675, 1541, 1250, 735; ESI-HRMS found *m/z* 430.1628 [M+H]$^+$, $C_{26}H_{24}NO_5$ requires 430.1649.

Methyl 3-(*sec*-butoxy)-4-nitrobenzoate 3d

Procedure B; Methyl-3-hydroxy-4-nitro-benzoate **2** (2.42 g, 12.28 mmol), S(+)-*sec*-butanol (1.00 g, 13.5 mmol), triphenylphosphine (4.82 g, 18.4 mmol) with diisopropyl azodicarboxylate (3.61 mL, 18.4 mmol) in tetrahydrofuran (80 mL). Work up followed by column chromatography yielded the product (2.73 g, 10.81 mmol, 88%) as a pale yellow liquid; d$_H$ (500 MHz, $CDCl_3$) 1.00 (3H, t, *J* = 7.4, H?), 1.36 (3H, d, *J* = 6.1, $CH_a(C\underline{H_3})$), 1.69-1.83 (2H, m, Hß + Hß'), 3.96 (3H, s, $CO_2\underline{Me}$), 4.56 (1H, m, Ha), 7.64 (1H, d, *J* = 8.3, H6), 7.73 (1H, s, H2), 7.76 (1H, d, *J* = 8.3, H5); d$_C$ (125MHz, $CDCl_3$) 9.5, 18.9, 29.0, 52.8, 77.7, 116.7, 120.9, 125.1, 134.4, 143.6, 151.1, 165.4, ?$_{max}$/cm^{-1} (solid state) =

2980, 1731, 1606, 1531, 1294, 1108, 1094; ESI-HRMS found *m/z* 276.0823$[M+Na]^+$, $C_{12}H_{15}NO_5$ requires 276.0842.

4-((((9H-fluoren-9-yl) methoxy)carbonyl)amino)-3-(sec-butoxy)benzoic acid 1d

4-Amino-3-(*sec*-butoxy)benzoic acid **5d** was obtained from methyl 4-amino-3-(*sec*-butoxy)benzoate **4d** by *procedure F* without purification/ isolation**.** Methyl 4-amino-3-(*sec*-butoxy)benzoate **4d** was in turn obtained from methyl 3-(*sec*-butoxy)-4-nitrobenzoate **3d** by *procedure C* without purification/ isolation. *Procedure H*; 4-amino-3-(sec-butoxy)benzoic acid **5d** (2.00 g, 9.56 mmol) in tetrahydrofuran (100 mL), fluorenylmethyloxycarbonyl chloride (3.45 g, 13.4 mmol) in tetrahydrofuran (30 mL). The crude material obtained after the reaction was purified by colum chromatography to yield the title compound (3.18 g, 7.37 mmol, 77%) as an off-white amorphous solid; (Found C, 71.60; H, 5.90; N, 3.10%. $C_{26}H_{25}N_1O_5$ requires C, 72.37; H, 5.84; N, 3.25%); d_H (500 MHz,$CDCl_3$) 1.05 (3H, t, *J* = 7.4, H?), 1,39 (3H, d, *J* = 6.0, $CH_aC\underline{H_3}$), 1.75 (1H, m, Hß), 1.84 (1H, m, Hß'), 4.34 (1H, t, *J* = 7.0, FHß), 4.48-4.53 (3H, m, Ha + FHa), 7.33 (2H, t, *J* = 7.3, FHAr4), 7.43 (2H, t, *J* = 7.3,FHAr3), 7.56-7.63 (4H, m, H2, H5 + FHAr5), 7.74 (1H, d, *J* = 8.5, H6), 7.80 (2H, d, *J* = 7.5, FHAr2), 8.15 (1H br. s, N$\underline{H}$); d_C (125MHz, $CDCl_3$) 9.7, 19.3, 29.1, 47.1, 67.5, 113.7, 117.4, 120.1, 123.0, 124.1, 125.0, 127.2, 127.9, 133.7, 143.7, 145.5, 153.0, 171.7, $?_{max}/cm^{-1}$ (solid state) = 3427, 2960, 2565, 1747, 1690, 1595, 1535, 1483, 1416, 1345, 1301, 1213, 1189, 1058; $[\alpha]_D^{24}$ -12.8 (*c* = 1, chloroform); ESI-HRMS found *m/z* 430.1660 $[M-H]^-$, $C_{26}H_{24}NO_5$ requires 430.1654.

Methyl 4-amino-3-(benzyloxy)benzoate 4e

Procedure C; Methyl-3-benzyloxy-4-nitrobenzoate **3e** (4.00 g, 13.9 mmol), tin(II) chloride dihydrate (18.84 g, 83.6 mmol) in ethyl acetate (100 mL). Following initial work-up, the solvents were removed under reduced pressure and the resultant orange oil was passed through a bed of silica (20% diethyl ether / dichloromethane) and the solvents removed. The resultant solid was crystallised from hexane to yield the title compound (2.58 g, 10.0 mmol, 72%) as colourless square plates; m.p. 82.2-83.6 °C (hexane); (Found C, 69.75; H, 5.85; N, 5.2%. CHN requires C, 70.02; H, 5.88; N, 5.44%); d_H (300 MHz, $CDCl_3$), 3.90 (3H, s, $CO_2\underline{Me}$), 4.34 (2H, br s, $N\underline{H_2}$), 5.14 (2H, s, Ha), 6.72 (1H, d, *J* = 6.6, H5), 7.30-7.50 (5H, m, HAr2, HAr3 + HAr4), 7.60-7.63 (2H, m, H6 + H2); d_C (75 MHz, $CDCl_3$) 52.1, 70.9, 113.1, 113.7, 119.7, 124.8, 128.2, 128.6, 129.0, 137.1, 141.9, 145.7, 167.7; $?_{max}/cm^{-1}$ (solid state) = 3517, 3395, 2930, 1688, 1432, 1279, 1128, 796; ESI-HRMS found *m/z* 258.1117 $[M+H]^+$, $C_{15}H_{16}NO_3$ requires 258.1125.

4-Amino-3-(benzyloxy)benzoic acid 5e

Procedure F; Methyl 4-amino-3-(benzyloxy)benzoate **4e** (5.44 g, 21.1 mmol), in a 1:1 mixture of methanol: tetrahydrofuran (135 mL), 10% aqueous sodium hydroxide (40 mL). Following acidification, the precipitate was filtered, dissolved in chloroform and dried (magnesium sulphate). This solution was then filtered and the organic solvents removed under reduced pressure to yield the title compound (3.90g, 16.0 mmol, 76%) as a colourless amorphous powder; d_H (300 MHz, MeOD-d_4)

5.17 (2H, s, Ha), 6.75 (1H, d, *J* = 4.9, H5), 7.34 (1H, t, *J* = 4.5, HAr4), 7.39-7.42 (2H, t, *J* = 4.5, HAr3), 7.49-7.53 (4H, m, H6, H2 + HAr2); d_C (75MHz, MeOD-d_4) 70.4, 113.0, 113.1, 118.5, 124.8, 127.6, 127.9, 128.5, 137.4, 143.2, 145.3, 169.5; $?_{max}/cm^{-1}$ (solid state) 3353, 2920, 2256, 1690, 1619, 1522, 1442, 1252, 1146, 1024, 879; ESI-HRMS found *m/z* 244.0973 $[M+H]^+$, $C_{14}H_{14}NO_3$ requires 244.0986;

4-(((9H-fluoren-9-yl)methoxy)carbonylamino)-3-(benzoyloxy)benzoic acid 1e

Procedure H; 4-Amino-3-(benzyloxy)benzoic acid **5d** (7.00 g, 28.8 mmol) in tetrahydrofuran (100 mL), fluorenylmethyloxycarbonyl chloride (11.17 g, 43.2 mmol) in tetrahydrofuran (100 mL). Following work up, the resulting solid was crystallised from a 1.5:1 solution of chloroform / methanol to yield the title compound (12.32 g, 26.5 mmol, 92%) as colourless microcrystals; m.p. 242.3-243.8 °C (chloroform / methanol); d_H (500 MHz, DMSO-d_6) 4.37 (1H, t, *J* = 6.9, FHß), 4.50 (2H, d, *J* = 6.9, FHa), 5.31 (2H, s, Ha), 7.36-7.40 (3H, m, HAr4 +FHAr4), 7.43-7.50 (4H, m, HAr3 + FHAr3), 7.56-7.58 (3H, m, H5 + HAr2), 7.62 (1H, s, H2), 7.75-7.81 (3H, m, H6 + FHAr5) 7.96 (2H, d, *J* =7.5, FHAr2), 8.96 (1H, br. s, N<u>H</u>); d_C (125MHz, DMSO-d_6); 46.9, 66.7, 70.3, 113.5, 120.5, 121.2, 122.8, 125.6, 126.5, 127.4, 127.6, 128.1, 128.8, 132.1, 137.1, 141.1, 144.0, 146.4, 148.7, 153.8, 167.2; $?_{max}/cm^{-1}$ (solid state) = 3332, 2891, 1671, 1597, 1501, 1435, 1346, 1217, 1104, 984, 878; ESI-HRMS found *m/z* 488.1488 $[M+Na]^+$, $C_{29}H_{23}NNaO_5$ requires 488.1468;

Methyl 3-((4-(tert-butyl)benzyl)oxy)-4-nitrobenzoate 3f

Procedure A; Methyl-3-hydroxy-4-nitro-benzoate **2** (3.40 g, 17.3 mmol) potassium carbonate (7.15 g, 51.7 mmol), in dimethlyformamide (70 mL), 4-*tert* butyl benzyl bromide (4.10 mL, 22.4 mmol). Work up yielded the title compound (5.98 g, 169.0 mmol, 98%) as a pale yellow amorphous solid; (Found C, 66.25; H, 6.35; N, 4.10%. $C_{19}H_{21}N_1O_5$ requires C, 66.46; H, 6.16; N, 4.08%); d_H (500 MHz, $CDCl_3$) 1.33 (9H, s, $C(C\underline{H}_3)_3$), 3.96 (3H, s, $CO_2\underline{Me}$), 5.25 (2H, s, Ha), 7.44-7.39 (4H, m, HAr2 + HAr3), 7.73 (1H, dd, *J* = 8.4, 1.3, H6), 7.86-7.88 (2H, m, H2 + H5); d_C (75MHz, $CDCl_3$) 31.3, 34.7, 52.8, 71.4, 116.1, 121.6, 125.3, 125.7, 127.2, 132.0, 134.8, 142.9, 151.55, 151.58, 165.2; $?_{max}/cm^{-1}$ (solid state) = 2963, 1725, 1608, 1538, 1439, 1372, 1291, 1247, 1111, 1087; ESI-HRMS found *m/z* 366.1315 $[M+Na]^+$, $C_{19}H_{21}NNaO_5$ requires 366.1317.

Methyl 4- amino-3-((4-(tert-butyl) benzyl)oxy)-4-nitrobenzoate 4f

Procedure C: Methyl 3-((4-(tert-butyl)benzyl)oxy)-4-nitrobenzoate **3f** (5.70 g, 16.6 mmol), tin(II) chloride dihydrate (18.60 g, 82.7 mmol) in ethyl acetate (120 mL). Work up yielded the title compound (4.59 g, 14.7 mmol, 88%) as a yellow amorphous solid; d_H (500 MHz, $CDCl_3$) 1.35 (9H, s, $C(C\underline{H}_3)_3$), 3.88 (3H, s, $CO_2\underline{Me}$), 4.26 (2H, br. s, $N\underline{H}_2$), 5.09 (2H, s, Ha), 6.69 (1H, d, *J* = 8.4, H5), 7.39-7.45 (4H, m, HAr2 + HAr3), 7.57-7.59 (2H, m, H2 + H6); d_C (125MHz, $CDCl_3$) 31.4, 34.6, 51.7, 70.4, 112.6, 113.3, 119.5, 124.3, 125.6, 127.8, 133.7, 141.4, 145.4, 151.3, 167.3; $?_{max}/cm^{-1}$ (solid state) = 3476, 3352, 3199, 2958, 1682, 1620, 1593, 1523, 1441, 1316, 1151, 1109, 1034; ESI-HRMS found *m/z* 336.1577 $[M+Na]^+$, $C_{19}H_{23}NNaO_5$ requires 336.1576.

4- amino-3-((4-(tert-butyl)benzyl)oxy)-4-nitrobenzoic acid 5f

Procedure F; 4-amino-3-((4-(tert-butyl)benzyl)oxy)-4-nitrobenzoicacid **4f** (4.50 g, 14.3 mmol) in a 1:1 mixture of methanol-tetrahydrofuran (110 mL), 10% aqueous sodium hydroxide (25 mL). Work up yielded the title compound (4.05 g, 13.5 mmol, 94%) as a beige amorphous solid; d_H (500 MHz, $CDCl_3$) 1.35 (9H, s, C(C<u>H</u>$_3$)$_3$), 5.11 (2H, s, Ha), 6.71 (1H, d, *J* = 8.0, H5), 7.39-7.45 (4H, m, HAr2 + HAr3), 7.62 (1H, d, *J* = 1.5, H2), 7.66 (1H, dd, *J* = 8.0, 1.5, H6); $?_{max}/cm^{-1}$ (solid state) = 3468, 3367, 2962, 1671, 1614, 1519, 1442, 1407, 1370, 1442, 1407, 1370, 1220, 1148, 1107; ESI-HRMS found *m/z* 322.1414 $[M+Na]^+$, $C_{18}H_{21}NNaO_3$ requires 322.1419.

4-((((9H-fluoren-9-yl) methoxy)carbonyl)amino)-3-((4-(tert-butyl)benzyl)oxy)benzoic acid 1f

Procedure H; 4-amino-3-((4-(tert-butyl) benzyl)oxy)-4-nitrobenzoicacid **5f** (3.50 g, 11.7 mmol) in tetrahydrofuran (70 mL), fluorenylmethyloxycarbonyl chloride (4.53 g, 17.5 mmol) in tetrahydrofuran (45 mL). Work up yielded the title compound (5.20 g, 10.0 mmol, 85%) as a white amorphous solid; (Found C, 75.85; H, 6.00; N, 2.60%. $C_{33}H_{31}NO_5$ requires C, 75.99; H, 5.99; N, 2.69%); d_H (500 MHz, $CDCl_3$) 1.37 (9H, s, C(C<u>H</u>$_3$)$_3$), 4.31 (1H, t, *J* = 7.0, FHß), 4.52 (2H, d, *J* = 7.0, FHa), 5.17 (2H, s, Ha), 7.31(2H, t, *J* = 7.5, FHAr4), 7.39-7.42 (4H, m, HAr3 + FHAr3), 7.48 (2H, d, *J* = 8.2, HAr2), 7.56 (1H, m, H5), 7.60 (2H, d, *J* = 7.6, FHAr5), 7.71 (1H, s, H2), 7.77-7.79 (3H, m, H6 + FHAr2); d_C (75MHz, $CDCl_3$) 31.3, 34.7, 46.9, 67.4, 70.9, 112.5, 117.3, 120.1, 123.2, 124.6, 125.0, 125.8, 127.1, 127.75, 127.8, 132.7, 133.0, 141.3, 143.6, 146.3, 151.7, 152.9, 171.2; $?_{max}/cm^{-1}$ (solid state) = 3430, 2958, 1740, 1681, 1595, 1534, 1489, 1351, 1242, 1224, 1199, 1058; ESI-HRMS found *m/z* 520.2139 $[M-H]^-$, $C_{33}H_{30}NO_5$ requires 520.2124.

Methyl 3-((4-chlorobenzyl)oxy)-4-nitrobenzoate 3g

Procedure A; Methyl-3-hydroxy-4-nitrobenzoate **2** (10.00 g, 50.7 mmol), potassium carbonate (2l.0 g, 151.9 mmol) in dimethylformamide (200 mL), 1-(bromomethyl)-4-chlorobenzene (12.5 g, 60.8 mmol). Following work-up, the resulting solid was crystallised (dichloromethane/ methanol) to yield the title compound (14.83 g, 46.1 mmol, 91%) as pale yellow crystalline plates; m.p. 133.7-134.8°C (dichloromethane/ methanol); (Found C, 55.75; H, 3.70; N, 4.30%. $C_{15}H_{12}NO_5Cl$ requires C, 56.00; H, 3.76; N, 4.35%); d_H (300 MHz, $CDCl_3$) 4.00 (3H, s, CO$_2$<u>Me</u>), 5.29 (2H, s, Ha), 7.42 (4H, m, HAr2 + HAr3), 7.76 (1H, d, *J* = 8.4, H6), 7.84 (1H, s, H2), 7.90 (1H, d, *J* = 8.4, H5); d_C (75MHz, $CDCl_3$) 53.4, 71.0, 116.3, 122.4, 125.9, 128.9, 129.4, 133.9, 134.7, 135.3, 142.9, 151.6, 166.5; $?_{max}/cm^{-1}$ (solid state) =3059, 1726. 1610, 1524, 1296, 1035, 808, 485; ESI-HRMS found *m/z* 344.0306 $[M+Na]^+$, $C_{15}H_{12}ClNNaO_5$ requires 344.0296.

Methyl 4-amino-3-((4-chlorobenzyl)oxy)benzoate 4g

Procedure C; Methyl 3-((4-chlorobenzyl)oxy)-4-nitrobenzoate **3g** (5.00 g, 15.5 mmol), tin(II) chloride dihydrate (21.00 g, 93.1 mmol) in ethyl acetate (100 mL). Following work up, the resulting solid was crystallised (dichloromethane/ hexane) to yield the title compound (3.03 g, 10.4 mmol, 67%) as pale yellow microcrystals; m.p. 118.9-119.6°C (dichloromethane/ hexane); (Found C, 61.80;

H, 4.75; N, 4.70%. $C_{15}H_{14}ClNO_3$ requires C,61.76; H, 4.84; N, 4.80%); d_H (300 MHz, $CDCl_3$) 3.86 (3H, s, H CO_2Me), 4.25 (2H, s, NH_2), 5.09 (2H, s, Ha), 6.69 (1H, d, *J* = 8.1, H5), 7.37 (4H, m, HAr2 + HAr3), 7.52 (1H, d, *J* = 1.5, H2), 7.57 (1H, dd, *J* = 8.1+ 1.5, H6); d_C (125MHz, $CDCl_3$) 50.1, 68.1, 111.0, 111.8, 117.9, 122.9, 127.2, 127.5, 132.4, 133.5, 139.7, 143.4, 165.5; $?_{max}/cm^{-1}$ (solid state) = 3502, 3380, 2944, 1690, 1445, 1292, 1104, 800, 484; ESI-HRMS found *m/z* 292.0740 $[M+H]^+$, $C_{15}H_{15}ClNO_3$ requires 292.0735.

4-Amino-3-((4-chlorobenzyl)oxy)benzoic acid 5g

Procedure F; Methyl 4-amino-3-((4-chlorobenzyl)oxy)benzoate **4g** (2.34 g, 8.02 mmol) in a 1:1 mixture of methanol-tetrahydrofuran (60 mL), 10% aqueous sodium hydroxide (18 mL). The resulting precipitate was filtered, dissolved in chloroform and dried with magnesium sulfate. The solution was filtered and the organic solvents removed under reduced pressure yielding the title compound (2.06 g, 7.4 mmol, 93%) as a colourless amorphous solid; (Found C, 60.60; H, 4.40; N, 4.90%. $C_{14}H_{12}ClNO_3$ requires C, 60.55; H, 4.36; N, 5.04%); d_H (500 MHz, MeOD-d_4) 5.16 (2H, s, Ha), 6.74 (1H, d, *J* = 8.5, H5), 7.40 (2H, d, *J* = 8.5, HAr3), 7.49 (2H, d, *J* = 8.5, HAr2), 7.51-7.54 (2H, m, H6 + H2); d_C (125MHz, MeOD-d_4) 70.6, 114.2, 114.2, 119.5, 126.0, 129.7, 130.3, 134.8, 137.4, 144.4, 146.2, 170.5; $?_{max}/cm^{-1}$ (solid state) = 3438, 3293, 2563, 1688, 1296, 764, 495; ESI-HRMS found *m/z* 278.0565 $[M+H]^+$, $C_{14}H_{13}ClNO_3$ requires 278.0578.

4-((((9H-fluoren-9-yl)methoxy)carbonyl)amino)-3-((4-chlorobenzyl)oxy)benzoic acid 1g

Procedure H; 4-Amino-3-((4-chlorobenzyl)oxy)benzoic acid **5g** (1.92 g, 6.9 mmol) in tetrahydrofuran (100 mL), fluorenylmethyloxycarbonyl chloride (2.68 g, 10.4 mmol) in chloroform (30 mL). Work up yielded the title compound (2.93 g, 5.9 mmol, 85%) as a colourless amorphous solid; (Found C, 69.65; H, 4.40; N, 2.65%. $C_{29}H_{22}ClNO_5$ requires C, 69.67; H, 4.44; N, 2.80%); d_H (300 MHz, DMSO-d_6) 4.32 (1H, t, *J* = 6.9, FHß), 4.46 (2H, d, *J* = 6.9, FHa), 5.25 (2H, s, Ha), 7.32 (2H, t, *J* = 7.5, FHAr4), 7.29-7.35 (4H, m, HAr3 + FHAr3), 7.41-7.58 (4H, m, H5, H2 + HAr2), 7.69 (1H, d, *J* = 8.2, H6), 7.74 (2H, d, *J* = 7.5, FHAr5), 7.91 (2H, d, *J* = 7.5, FHAr2), 9.01 (1H, s, NH); d_C (75MHz, DMSO-d_6) 46.5, 66.2, 69.0, 113.1, 120.1, 121.1, 122.5, 125.2, 126.2, 127.1, 127.7, 128.4, 129.3, 131.7, 132.4, 135.8, 140.7, 143.7, 148.3, 153.5, 166.8; $?_{max}/cm^{-1}$ (solid state) = 3305, 2805, 1697, 1678, 1544, 1256, 739, 484; ESI-HRMS found *m/z* 522.1065 $[M+Na]^+$, $C_{29}H_{22}ClNaNO_5$ requires 522.1079.

Methyl 4-amino-3-(naphthalen-2-ylmethoxy)benzoate 4h

Procedure C; Methyl-3-(2-napthyl)methoxy-4-nitrobenzoate **3h** (6.25 g, 18.5 mmol), tin(II) chloride dihydrate (25.00 g, 110.8 mmol) in ethyl acetate (130 mL). Work afforded the title compound (4.38 g, 14.3 mmol, 77%) as a cream amorphous solid; (Found C, 74.4; H, 5.55; N, 4.35%. $C_{19}H_{17}NO_3$ requires C, 74.25.; H, 5.58; N, 4.65%); d_H (500 MHz, $CDCl_3$) 3.83 (3H, s, CO_2Me), 4.31 (2H, br s, NH_2), 5.26 (2H, s, Ha), 6.67 (1H, d, *J* = 8.1, H5), 7.45-7.60 (5H, m, HAr), 7.80-7.86 (4H, m, HAr); d_C (75MHz, $CDCl_3$) 52.0, 71.1, 113.1, 113.7, 119.7, 124.8, 125.9, 126.6, 126.7, 127.1, 128.1, 128.4,

128.8, 133.6, 133.7, 134.5, 142.0, 156.7, 167.6; $?_{max}/cm^{-1}$ (solid state) = 3373, 2862, 1698, 1618, 1440, 1254, 1023; ESI-MS found *m/z* 308 $[M+H]^+$;

4-Amino-3-(naphthalen-2-ylmethoxy)benzoic acid 5h

Procedure F; Methyl 4-amino-3-(naphthalen-2-ylmethoxy)benzoate **4h** (4.00g, 13.0 mmol) in a 1:1 mixture of methanol-tetrahydrofuran (100 mL), 10% aqueous sodium hydroxide (25 mL). Following acidification, the precipitate was filtered, dissolved in chloroform and dried with magnesium sulfate. This solution was filtered and the organic solvents removed under reduced pressure and the solid was crystallised (chloroform/ methanol) to yield the title compound (3.19 g, 10.9 mmol, 84 %) as a colourless microcrystals; m.p. 161.8-163.5 (chloroform/ methanol); d_H (500 MHz, DMSO-d_6) 5.32 (2H, s, Ha), 6.70 (1H, d, *J* = 8.2, H5), 7.38 (1H, d, *J* = 8.2, HAr), 7.45 (1H, s, H2), 7.50-7.54 (2H, m, HAr), 7.63 (1H, d, *J* = 8.1, H6), 7.91-7.95 (3H, m, HAr), 8.04 (1H, s, HAr); d_C (75 MHz, DMSO-d_6); 69.8, 112.9, 113.1, 117.7, 124.6, 125.8, 126.1, 126.4 126.6, 127.9, 128.1, 128.3, 132.9, 133.2, 135.2, 143.3, 144.4, 167.9; $?_{max}/cm^{-1}$ (solid state) = 3401, 3000, 1678, 1613, 1521, 1441, 1263, 1149, 1025, 764; ESI-HRMS found *m/z* 316. 0983 $[M+Na]^+$, $C_{18}H_{15}NNaO_3$ requires 316.0944;

4-(((9H-fluoren-9-yl)methoxy)carbonylamino)-3-(naphthalen-2-ylmethoxy)-benzoic acid 1h

Procedure H; 4-Amino-3-(naphthalen-2-ylmethoxy)benzoic acid **5h** (3.10 g, 10.6 mmol) in tetrahydrofuran (150 mL), fluorenylmethyloxycarbonyl chloride (4.10 g, 15.9 mmol) in tetrahydrofuran (50 mL). Following work up, the resulting solid which was crystallised (chloroform/ methanol) to yield the title compound (5.22 g, 9.1 mmol, 96%) as colourless microcrystals; m.p. 201.1-202.3 °C (chloroform/ methanol); d_H (300 MHz, DMSO-d_6) 4.31 (1H, t, *J* = 6.9, FHß), 4.46 (2H, d, *J* = 6.9, FHa), 5.36 (2H, s, Ha), 7.29 (2H, t, *J* = 7.4, FHAr4), 7.45 (2H, t, *J* = 5.4, FHAr3), 7.50-7.56 (3H, m, HAr), 7.63 (1H, s, H2), 7.66 (1H, d, *J* = 8.4, H6), 7.72-7.74 (3H, m, ArCH), 7.85-7.96 (5H, m, ArCH), 8.04 (1H, s, H8), 8.93 (1H, s, NH); d_C (75 MHz, DMSO-d_6); 46.9, 66.7, 70.4, 113.6, 120.5, 121.3, 122.9, 125.6, 125.8, 126.4, 126.5, 126.7, 127.4, 128.0, 128.1, 128.2, 128.4, 132.2, 132.9, 133.1, 132.7, 141.1, 144.0, 148.8, 153.8, 167.2; $?_{max}/cm^{-1}$ (solid state) = 3423, 2927, 1673, 1602, 1440, 1216, 1034, 816, 762; ESI-HRMS found *m/z* 516.1800 $[M+H]^+$, $C_{33}H_{26}NO_5$ requires 516.1805.

Methyl 4-nitro-3-(4-trifluoromethyl)benzyloxy)benzoate 3i

Procedure A; Methyl-3-hydroxy-4-nitrobenzoate **2** (1.00 g, 5.1 mmol), potassium carbonate (2.l0 g, 15.2 mmol) in dimethylformamide (20 mL), 4-(trifluoromethyl) benzyl bromide (0.94 mL, 6.0 mmol) Following initial work-up, the solvents were removed under reduced pressure, dissolved in methanol, filtered to remove starting material and left to crystallise by slow evaporation to yield the title compound (1.05g, 3.0 mmol, 61%) as pale yellow plates; mp. 97.8-100.2°C (methanol); (Found C, 54.25; H, 3.35; N, 3.80%. $C_{16}H_{12}NO_5F_3$ requires C, 54.09.; H, 3.40; N, 3.94%); d_H (500 MHz, $CDCl_3$) 3.98 (3H, s, CO_2Me), 5.26 (2H, s, Ha), 7.53 (d, 2H, *J* = 8.2, HAr3), 7.60 (2H, d, *J* = 8.2, HAr2), 7.67 (1H, d, *J* = 8.2, H6), 7.74 (1H, s, H2), 7.81 (1H, d, *J* = 8.2, H5); d_C (125 MHz, $CDCl_3$); 54.7, 72.3,

117.7, 124.0, 125.8 (q, *J* = 270), 127.4, 127.6, 129.0, 132.4 (q, *J* = 32.5), 136.8, 140.9, 144.6, 152.9, 166.9; ?$_{max}$/cm^{-1} (solid state) = 3429, 2955, 1923, 1723 1591, 1334, 831, 590, 493; ESI-HRMS found *m/z* 378.0569 [M+Na]$^+$, $C_{16}H_{12}F_3NNaO_3$ requires 378.0560;

Methyl 4-amino-3-(4-(trifluoromethyl)benzyloxy)benzoate 4i

Procedure C; Methyl 4-nitro-3-(4-trifluoromethyl)benzyloxy)benzoate **3i** (1.00 g, 2.9 mmol), tin(II) chloride dihydrate (3.81 g, 16.9 mmol) in ethyl acetate (30 mL). Work up resulted in the title compound (0.93 g, 1.5 mmol, 97%) as a yellow amorphous solid; d$_H$ (500 MHz, MeOD-d$_4$) 3.79 (3H, s, CO$_2$Me) 4.19 (2H, s, NH$_2$), 5.12 (2H, s, Ha), 6.63 (1H, d, *J* = 8.2, H5), 7.46 (1H, d, *J* = 1.4, H2), 7.49-7.52 (3H, m, H6 + HAr2), 7.59 (2H, d, *J* = 8.1, HAr3); d$_C$ (75 MHz, $CDCl_3$) 52.6, 70.5, 113.5, 114.4, 120.4, 125.5, 126.6, 128.6, 129.7, (q, *J* = 270), 131.0 (q, *J* = 32.3) 141.5, 142.1, 145.7, 170.0; ?$_{max}$/cm^{-1} (solid state) = 3490, 3380, 2927, 1740, 1704, 1615, 1262, 1066, 705, 505; ESI-HRMS found *m/z* 326.0869 [M+H]$^+$, $C_{16}H_{15}F_3NO_3$ requires 326.0999;

4-amino-3-(4-(trifluoromethyl)benzyloxy)benzoic acid 5i

Procedure F; Methyl 4-amino-3-(4-(trifluoromethyl)benzyloxy)benzoate **4i** (0.90 g, 2.7 mmol) in a 1:1 mixture of methanol : tetrahydrofuran (24 mL), 10% aqueous sodium hydroxide (6.0 mL). Following acidification, the resulting precipitate was filtered, dissolved in chloroform and dried with magnesium sulfate. This solution was filtered and the organic solvents removed under reduced pressure yielding a pale yellow solid which was crystallised (chloroform) yielding the title compound (0.53 g, 1.7 mmol, 62%) as fine colourless microcrystals; m.p. 192.4 – 194.1°C (chloroform); d$_H$ (500 MHz, MeOD-d$_4$) 5.14 (2H, s, Ha), 6.63 (1H, d, *J* = 8.4, H5), 7.40-7.42 (2H, m, H6 + H2), 7.58 (4H, m, HAr2, + HAr3); d$_C$ (75 MHz, $CDCl_3$) 70.9, 79.9, 114.6 (q, *J* = 8.0), 119.9, 126.1 (q, *J* = 269.3), 126.5, 126.8 (q, *J* = 3.8), 129.2, 131.4 (q, *J* = 32.0), 143.5, 144.8, 146.4, 170.9; ?$_{max}$/cm^{-1} (solid state) = 3434, 3291, 3116, 2926, 2526, 2159, 2026, 1688, 1296, 1108, 765; ESI-HRMS found *m/z* 312.0856 [M+H]$^+$, $C_{15}H_{13}F_3NO_3$ requires 312.0842;

4-((((9H-fluoren-9-yl)methoxy)carbonyl)amino)-3-((4-(trifluoromethyl)benzyl)oxy)benzoic acid 1i

Procedure H; 4-amino-3-(4-(trifluoromethyl)benzyloxy)benzoic acid **5i** (0.46 g, 1.5 mmol) in chloroform (100 mL), fluorenylmethyloxycarbonyl chloride (0.58 g, 2.2 mmol) in chloroform (50 mL). Work up yielded the title compound (0.60 g, 1.1 mmol, 76%) as a colourless amorphous solid; (Found C, 67.55; H, 4.05; N, 2.25%. $C_{30}H_{22}F_3NO_5$ requires C, 67.54; H, 4.16; N, 2.63%); d$_H$ (500 MHz, DMSO-d$_6$) 4.32 (1H, t, *J* = 6.9, FHß), 4.46 (2H, d, *J* = 6.9, FHa), 5.37 (2H, s, Ha), 7.31 (2H, t, *J* = 7.5, FHAr4), 7.43 (2H, t, *J* = 7.5, FHAr3), 7.60 (2H, d, *J* = 10.5, H5 + H2), 7.70 (1H, d, *J* = 8.4, H6), 7.75 (6H, m, HAr2, HAr3 + FHAr5), 7.91 (2H, *J* = 6.3, FHAr2), 9.10 (1H, s, NH); d$_C$ (75MHz, DMSO-d$_6$) 46.7, 66.6, 69.3, 113.4, 120.4, 121.7, 122.8, 125.6, 126.7, 127.4, 128.1, 128.2, 128.5, 129.1 (q, *J* = 30), 132.1, 141.1, 142.0, 144.0, 148.7, 153.9, 167.2; ?$_{max}$/cm^{-1} (solid state) = 3309, 3200-2200 (br), 1697, 1543, 1256, 1114, 739; ESI-HRMS found *m/z* 534.1500 [M+H]$^+$, $C_{30}H_{23}F_3NO_5$ requires 534.1523;

Methyl 4-nitro-3-(3-trifluoromethyl)benzyloxy)benzoate 3j

Procedure A; Methyl-3-hydroxy-4-nitrobenzoate **2** (1.00 g, 5.1 mmol), potassium carbonate (2.l0 g, 15.2 mmol) in dimethylformamide (20 mL), 3-(trifluoromethyl) benzyl bromide (0.94 mL, 6.0 mmol) Following preliminary work-up, the organic solvent was removed under reduced pressure and the solid dissolved in methanol, filtered to remove 4-(trifluoromethyl) benzyl bromide starting material and left to crystallise by slow evaporation to yield the title compound (1.25 g, 3.5 mmol, 69%) as pale yellow microcrystals; m.p. 105.9 – 107.4 °C (methanol); (Found C, 54.10; H, 3.35; N, 3.80%. $C_{16}H_{12}NO_5F_3$ requires C, 54.09; H, 3.40; N, 3.94%); d_H (500 MHz, $CDCl_3$) 3.90 (3H, s, $CO_2\underline{Me}$), 5.26 (2H, s, Ha), 7.48 (1H, apparent t, *J* = 7.5, HAr5), 7.55 (1H, d, *J* = 7.5, HAr4), 7.63 (1H, d, *J* = 7.5, HAr6), 7.66-7.68 (2H, m, H6 + HAr2), 7.75 (1H, s, H2), 7.82 (1H, d, *J* = 8, H5); d_C (75 MHz, $CDCl_3$) 53.3, 71.0, 116.4 (d, *J* = 10.4), 122.6 (d, *J* = 11.8), 124.3 (q, *J* = 162.4), 124.3 (d, *J* = 9.3), 125.9, 126.1, 129.8 (d, *J* = 11.7), 130.8 (d, J = 11.8), 131.5 (q, *J* = 19.4), 135.4, 136.5, 143.1, 151.5, 165.4; $?_{max}/cm^{-1}$ (solid state) = 3439, 2955, 1952, 1737, 1365, 1229; ESI-MS found 378.1 $[M+Na]^+$;

Methyl 4-amino-3-(3-(trifluoromethyl)benzyloxy)benzoate 4j

Procedure C; Methyl 4-nitro-3-(3-trifluoromethyl)benzyloxy)benzoate **3j** (1.00 g, 2.9 mmol), tin(II) chloride dihydrate (3.81 g, 16.9 mmol) in ethyl acetate (30 mL). Work-up yielded the title compound (0.93 g, 2.9 mmol, 97%) as a colourless amorphous solid; d_H (300 MHz, $CDCl_3$) 3.90 (3H, s, $CO_2\underline{Me}$), 4.31 (2H, s, $N\underline{H_2}$), 5.21 (2H, s, Ha), 6.75 (1H, d, *J* = 8.1, H5), 7.54-7.62 (2H, m, H6 + HAr6), 7.64-7.69, (3H, m, HAr2, HAr4 + HAr5), 7.76 (1H, s, H2); d_C (75 MHz, $CDCl_3$) 52.2, 70.2, 113.0, 113.9, 119.9, 124.8, 124.9, 125.1, 125.4, 125.5, 131.4, 138.0, 141.7, 145.3, 167.6; $?_{max}/cm^{-1}$ (solid state) = 3492, 3356, 2957, 1888, 1772, 1691, 1614, 1266, 1110, 764; ESI-HRMS found *m/z* 348.0817 $[M+Na]^+$, $C_{16}H_{14}F_3NNaO_3$ requires 348.0818;

4-amino-3-(3-(trifluoromethyl)benzyloxy)benzoic acid 5j

Procedure F; Methyl 4-amino-3-(3-(trifluoromethyl)benzyloxy)benzoate **4j** (0.63 g, 1.9 mmol) in a 1:1 mixture of methanol-tetrahydrofuran (20 mL), 10% aqueous sodium hydroxide (11 mL). Following acidification, the precipitate was filtered, dissolved in chloroform and dried with magnesium sulfate.. This solution was filtered and the organic solvents removed under reduced pressure yielding a pale yellow solid which was crystallised (chloroform) to yield the title compound (0.55 g, 1.8 mmol, 92%) as a colourless amorphous solid; (Found C, 57.40; H, 3.90; N, 4.25%. $C_{15}H_{12}NO_3F_3$ requires C, 57.88; H, 3.89; N, 4.50%); d_H (300 MHz, MeOD-d_4) 5.12 (2H, s, Ha), 6.63 (1H, d, *J* = 8.7, H5), 7.39-7.42 (2H, m, H6 + HAr6), 7.44-7.53 (2H, m, H2 + HAr5), 7.64-7.68 (2H, m, HAr2 + HAr4); d_C (75 MHz, MeOD-d_4) 70.8, 114.5, 149.6, 119.8, 125.5, 126.0, 126.0 (q, *J* = 270), 126.4, 130.7, 132.3 (q, *J* = 32), 132.6, 140.3, 144.7, 146.3, 170.8; $?_{max}/cm^{-1}$ (solid state) = 3515, 3418, 3379, 2927, 1879, 1761, 1673, 1524, 1227, 920, 765, 576; ESI-HRMS found *m/z* 312.0835 $[M+H]^+$, $C_{15}H_{13}F_3NO_3$ requires 326.1004;

4-((((9H-fluoren-9-yl)methoxy)carbonyl)amino)-3-((3-(trifluoromethyl)benzyl)oxy)benzoic acid 1j

Procedure H; 4-Amino-3-(3-(trifluoromethyl)benzyloxy)benzoic acid **5j** (0.42 g, 1.4 mmol) in chloroform (100 mL), fluorenylmethyloxycarbonyl chloride (0.58 g, 2.2 mmol) in chloroform (50 mL). Work up yielded the title compound (0.53 g, 1.0 mmol, 73%) as a colourless amorphous solid; d_H (300 MHz, DMSO-d_6) 4.31 (1H, t, *J* = 7.0, FHß), 4.45 (2H, d, *J* = 7.0, FHa), 5.35 (2H, s, Ha), 7.30 (2H, t, *J* = 6.4, FHAr4), 7.42 (2H, t, *J* = 6.4, FHAr3), 7.53 (1H, dd, *J* = 8.1, 1.5, H5), 7.60 (1H, d, *J* = 1.5, H2), 7.65-7.75 (4H, m, H6, HAr5 + FHAr5), 7.85-7.92 (3H, m, HAr6 + FHAr2), 7.97 (1H, s, HAr2), 9.14 (1H, s, N<u>H</u>); d_C (125MHz, DMSO-d_6) 46.5, 66.3, 69.0, 113.2, 120.1, 121.3, 122.6, 124.2 (q, *J* = 2.6), 124.6 (q, *J* = 2.6), 125.2, 126.7, 127.4, 128.1, 129.8, 131.3 (q, *J* = 29.9*)* 131.9, 132.1, 138.7, 141.1, 144.0, 148.8, 153.9, 167.2; $?_{max}/cm^{-1}$ (solid state) = 3347, 3200-2200 (br), 1705, 1436, 1334, 1120, 739; ESI-HRMS found *m/z* 534.1505 $[M+H]^+$, $C_{30}H_{23}F_3NO_5$ requires 534.1523;

Methyl 3-((4-(tert-butoxy)benzyl)oxy)-4-nitrobenzoate 3k

Procedure B; Methyl-3-hydroxy-4-nitrobenzoate **2** (3.50 g, 11.7 mmol), (4-(tert-butoxy) phenyl) methanol (0.96 g, 5.4 mmol) triphenylphosphine (2.12 g, 8.1 mmol) with diisopropyl azodicarboxylate (1.59 mL, 8.1 mmol) in tetrahydrofuran (100 mL). Column chromatography yielded the title compound (1.80 g, 5.0 mmol, 95%) as pale yellow solid; d_H (500 MHz, $CDCl_3$) 1.39 (9H, s, $C(CH_3)_3$), 3.99 (3H, s, CO_2<u>Me</u>), 5.26 (2H, s, Ha), 7.04 (2H, d, *J* = 8.2, HAr3), 7.39 (2H, d, *J* = 8.2, HAr2), 7.73 (1H, d, *J* = 8.2, 1.6, H6), 7.86-7.88 (2H, m, H2 + H5); d_C (75MHz, $CDCl_3$) 28.9, 52.8, 71.4, 78.7, 116.3, 121.7, 124.2, 125.3, 128.1, 129.7, 134.8, 142.9, 151. 5, 155.7, 165.2; $?_{max}/cm^{-1}$ (solid state) = 3309, 2980, 1725, 1508, 1237, 895, 744; ESI-HRMS found *m/z* 382.1255 $[M+Na]^+$, $C_{19}H_{21}NNa_1O_6$ requires 382.1267.

3-((4-(tert-butoxy)benzyl)oxy)-4-nitrobenzoic acid 4k

Procedure F; Methyl 3-((4-(tert-butoxy)benzyl)oxy)-4-nitrobenzoate **3k** (2.24 g, 6.2 mmol) in a 1:1 mixture of methanol-tetrahydrofuran (60 mL), 10% aqueous sodium hydroxide (15 mL). Work up yielded the title compound (1.96 g, 5.7 mmol, 91%) as a yellow solid; d_H (500 MHz, MeOD-d_4)1.36 (9H, s, $C(CH_3)_3$), 5.18 (2H, s, Ha), 6.96 (2H, d, *J* = 8.5, HAr3), 7.29 (2H, d, *J* = 8.4, HAr2), 7.71 (dd, *J* = 8.5, 1.5, H6), 7.79-7.81 (2H, m, H2 + H5); d_C (125MHz, MeOD-d_4) 29.3, 72.3, 79.8, 117.6, 123.0, 125.2, 126.0, 128.9, 129.5, 132.1, 144.6, 152.4, 156.8, 167.7; $?_{max}/cm^{-1}$ (solid state) = 2977, 1692, 1607, 1520, 1506, 1434, 1294, 1250, 1162, 1109; ESI-HRMS found *m/z* 344.1156 $[M-H]^-$, $C_{18}H_{18}NO_6$ requires 344.1134.

4-amino-3-((4-(tert-butoxy)benzyl)oxy)benzoic acid 5k

Procedure E; 3-((4-(tert-butoxy)benzyl)oxy)-4-nitrobenzoic acid (0.50 g, 1.5 mmol), cobalt chloride hexahydrate (2.07 g, 8.7 mmol) and sodium borohydride (0.68 g, 174.0 mmol) in methanol (100 mL). Work up yielded the title compound (336 mg, 1.1 mmol, 74%) as an orange amorphous solid; d_H (500 MHz, $CDCl_3$) 1.36 (9H, s, $C(CH_3)_3$), 5.00 (2H, s, Ha), 6.64 (1H,

d, *J* = 8 , H5), 6.95 (2H, d, *J* = 8.5, HAr3), 7.27 (2H, d, *J* = 8.5, HAr2), 7.54 (1H, d, *J* = 1.6, H2), 7.58 (1H, dd, *J* = 8.2, 1.6, H6); d_C (125MHz, $CDCl_3$) 28.9, 70.4, 78.7, 113.1, 113.3, 118.4, 124.1, 125.3, 128.7, 131.2, 142.1, 145.3, 155.5, 171.7; $?_{max}/cm^{-1}$ (solid state) = 3491, 3384, 2978, 2932, 1692, 1613, 1509, 1421; ESI-HRMS found *m/z* 338.1355 $[M+H]^+$, $C_{18}H_{21}NNaO_4$ requires 338.1363.

4-((((9H-fluoren-9-yl)methoxy)carbonyl)amino)-3-((4-(tert-butoxy)benzyl)oxy)benzoic acid 1k

Procedure I; 4-amino-3-((4-(tert-butoxy)benzyl)oxy)benzoic acid **5k** (288 mg, 0.9 mmol), sodium bicarbonate (153 mg, 1.8 mmol) in tetrahydrofuran (15 mL) and fluorenylmethyloxycarbonyl chloride (354 mg, 1.4 mmol) in tetrahydrofuran (10 mL). The pure product was precipitated from a dichloromethane-hexane solution to leave a grey amorphous solid (302 mg, 0.6 mmol, 62%); d_H (500 MHz, $CDCl_3$) 1.31 (9H, s, $C(CH_3)_3$), 4.23 (1H, t, *J* = 6.8, FHß), 4.45 (2H, d, *J* = 6, FHa), 5.07 (2H, s, Ha), 6.99 (2H, d, *J* = 8.2, HAr2), 7.23-7.29 (4H, m, HAr3 + FHAr4), 7.35 (2H, t, *J* = 7.3, FHAr3), 7.47 (1H, br. s, H5), 7.53 (2H, d, *J* = 7.3, FHAr5), 7.62 (1H, s, H2), 7.69-7.72 (3H, m, H6 + FHAr2), 8.10 (1H, br. s, N<u>H</u>); d_C (125MHz, $CDCl_3$) 28.89, 47.01, 67.43, 71.00, 78.81, 128.69, 112.85, 117.38, 120.07, 123.20, 124.21, 124.62, 124.97, 127.14, 127.84, 130.36, 141.34, 143.59, 146.28, 155.86, 170.40, $?_{max}/cm^{-1}$ (solid state) = 3421, 2973, 1742, 1674, 1594, 1538; ESI-HRMS found *m/z* 560.2030 $[M+Na]^+$, $C_{33}H_{31}NNaO_6$requires 560.2044.

3-(2-(1H-indol-3-yl)ethoxy)-4-aminobenzoic acid 5l

Methyl 3-(2-(1H-indol-3-yl)ethoxy)-4-nitrobenzoate **3l** was obtained from methyl-3-hydroxy-4-nitrobenzoate **2** by *procedure B* without purification/isolation. Methyl 3-(2-(1H-indol-3-yl)ethoxy)-4-aminobenzoate **4l** was obtained from methyl 3-(2-(1H-indol-3-yl)ethoxy)-4-nitrobenzoate **3l** by *procedure C* without purification/isolation. *Procedure F*; Methyl 3-(2-(1H-indol-3-yl)ethoxy)-4-aminobenzoate **4l** (2.50 g, 8.1 mmol) in a 1:1 mixture of methanol-tetrahydrofuran (65 mL), 10% aqueous sodium hydroxide (15 mL). Work up yielded the title compound (1.90 g, 6.4 mmol, 80%) as a cream amorphous solid; d_H (500 MHz, DMSO-d_6) 3.18 (2H, t, *J* = 6.7, Hß), 4.19 (2H, t, *J* = 6.7 Ha), 5.46 (2H, br. s, N<u>H</u>$_2$), 6.62 (1H, d, *J* = 8.1, H5), 6.98 (1H, m, IHAr3), 7.07 (1H, m, IHAr4), 7.30-7.36 (4H, m, H2, H6, IHAr5 + IC<u>H</u>NH), 7.61 (1H, d, *J* = 7.7, IHAr2), 10.87 (1H, s, ICHN<u>H</u>); (125 MHz, DMSO-d_6) 24.8, 68.4, 110.6, 111.4, 111.9, 112.1, 117.3, 118.2, 118.3, 120.9, 123.3, 124.0, 127.3, 136.1, 142.8, 144.2, 167.5; $?_{max}/cm^{-1}$ (solid state) =3488, 3387, 250, 1882 (br) 1667, 1614, 1447, 1274, 745; ESI-HRMS found *m/z* 319.1048 $[M+Na]^+$, $C_{17}H_{16}N_2NaO_3$ requires 319.1053.

3-(2-(1H-indol-3-yl)ethoxy)-4-((((9H-fluoren-9-yl)methoxy)carbonyl)amino)benzoic acid 1l

Procedure H; 3-(2-(1H-indol-3-yl)ethoxy)-4-aminobenzoic acid **5l** (1.58 g, 5.33 mmol) in tetrahydrofuran (50 mL), fluorenylmethyloxycarbonyl chloride (2.07 g, 87.00 mmol) in tetrahydrofuran (20 mL). Work up yielded the title compound as a light grey amorphous solid (2.25 g, 4.3 mmol, 81%); d_H (500 MHz, DMSO-d_6) 3.25 (2H, t, *J* = 6.9, Hß), 4.30-4.38 (3H, m, Ha + FHß), 4.47 (2H, d, J = 6.9, FHa), 6.97 (1H, m, IHAr3), 7.06 (1H, m, IHAr4), 7.28 (1H, d, *J* = 2.1, IC<u>H</u>NH), 7.31-7.36 (3H, m, IHAr5 + FHAr4), 7.44 (2H, t, *J* = 7.4, FHAr3), 7.49-7.54 (2H, m, H2 + H5), 7.62 (1H, d, *J* = 7.7, IHAr2), 7.74-7.77 (3H, m, H6 + FHAr5), 7.92 (2H, d, *J* = 7.5, FHAr2), 8.86 (1H, s,

FNH), 10.92 (1H, s, ICHNH); (125MHz, DMSO-d$_6$) 24.6, 46.5, 66.4, 69.0, 110.3, 111.4, 112.4, 118.2, 118.5, 119.7, 120.2, 121.0, 122.3, 123.2, 125.3, 125.9, 127.2, 127.3, 127.8, 131.6, 136.2, 140.8, 143.7, 148.0, 153.3, 166.9; ?$_{max}$/cm^{-1} (solid state) = 3358, 2951, 1908, 1714, 1673, 1439,1231, 1053, 736; ESI-HRMS found *m/z* 541.1727 [M+Na]$^+$, $C_{32}H_{26}N_2NaO_5$ requires 541.1734.

Methyl 3-(2-(tert-butoxy)-2-oxoethoxy)-4-nitrobenzoate 3m

Procedure A; Methyl-3-hydroxy-4-nitrobenzoate **2** (5.00 g, 25.4 mmol), potassium carbonate (3.50 g, 25.4 mmol) in dimethylformamide (100 mL), *tert*-butyl 2-bromoacetate (4.44 mL, 30.4 mmol). Following work up, the resulting solid was crystallised (ethyl acetate) to yield the title compound (6.26 g, 20.1mmol, 79 %) as large pale yellow crystals; m.p. 73.9-74.9°C (ethyl acetate); (Found C, 54.05; H, 5.50; N, 4.50%. $C_{14}H_{17}NO_7$ requires C, 54.02; H, 5.50; N, 4.50%); d$_H$ (300 MHz, $CDCl_3$) 1.50 (9H, s, $C(CH_3)_3$), 3.97 (3H, s, CO_2Me), 4.74 (2H, s, Ha), 7.63 (1H, d, *J* = 1.5, H2), 7.75 (1H, dd, *J* = 8.4, 1.5, H6), 7.89 (1H, d, *J* = 8.4, H5); d$_C$ (75MHz, $CDCl_3$) 28.0, 52.9, 66.5, 79.9, 83.4, 115.7, 122.3, 125.6, 134.7, 150.9, 165.0, 166.3; ?$_{max}$/cm^{-1} (solid state) = 2981, 1730, 1533,1305, 1225,745; ESI-HRMS found *m/z* 334.0908 [M+Na]$^+$, $C_{14}H_{17}NNaO_7$ requires 334.0897.

3-(2-(tert-butoxy)-2-oxoethoxy)-4-nitrobenzoic acid 4m

Saponification of **3m** preceded reduction to prevent hydrolysis of the ^{t}Butyl group. *Procedure G*; Methyl 3-(2-(tert-butoxy)-2-oxoethoxy)-4-nitrobenzoate **3m** (3.00 g, 9.6 mmol) in tetrahydrofuran (100 mL), lithium hydroxide (0.40 g, 9.6 mmol) in water (100 mL). Following acidification the resulting precipitate was filtered, dissolved in chloroform and dried with magnesium sulfate. The solution was filtered and the organic solvents removed under reduced pressure yielding the title compound (1.72 g, 5.8 mmol, 60%) as a white amorphous powder; (Found C, 52.55; H, 5.00; N, 4.60%. $C_{13}H_{15}NO_7$ requires C, 52.53; H, 5.09; N, 4.71%); d$_H$ (300 MHz, $CDCl_3$) 1.42 (9H, s, $C(CH_3)_3$, 4.69 (2H, s, Ha), 7.62 (1H, d, *J* = 1.5, H2), 7.76 (1H, dd, *J* = 8.1, 1.5, H6), 7.83 (1H, d, *J* = 8.1, H5); d$_C$ (75 MHz, $CDCl_3$) 28.0, 66.5, 83.5, 116.2, 123.1, 125.7, 133.5, 150.9, 166.3, 169.4; ?$_{max}$/cm^{-1} (solid state) = 3118-2555, 2984, 1740, 1695, 1532, 1432, 1265, 788; ESI-HRMS found *m/z* 320.0740 [M+Na]$^+$, $C_{13}H_{15}NNaO_7$ requires 320.0741.

4-Amino-3-(2-(tert-butoxy)-2-oxoethoxy)benzoic acid 5m

Procedure D; 3-(2-(tert-butoxy)-2-oxoethoxy)-4-nitrobenzoic **4m** (2.00 g, 6.7 mmol) in methanol (20 mL), 10% palladium on charcoal (200 mg, 10 wt%) in methanol (10 mL)) and hydrogen gas. Work up yielded the title compound (1.66 g, 6.2 mmol, 81%) as a beige amorphous powder; d$_H$ (500 MHz, MeOD-d$_4$) 1.53 (9H, s, $C(CH_3)_3$), 4.65 (2H, s, Ha), 6.74 (1H, d, *J* = 8.3, H5), 7.39 (1H, d, *J* = 1.8, H2), 7.54 (1H, dd, *J* = 8.2, 1.7, H6); d$_C$ (125MHz, MeOD-d$_4$) 28.3, 31.2, 67.5, 114.5, 116.7, 118.8, 119.4, 125.7, 126.5, 145.7, 170.4; ?$_{max}$/cm^{-1} (solid state) = 3523, 3427, 3123-2565, 2988, 1740, 1685, 1537, 1454, 1276, 788; ESI-HRMS found *m/z* 268.1181 [M+H]$^+$, $C_{13}H_{18}NO_5$ requires 268.1179.

4-((((9H-fluoren-9-yl)methoxy)carbonyl)amino)-3-(2-(tert-butoxy)-2-oxoethoxy)benzoic acid 1m

Procedure I; 4-amino-3-(2-(tert-butoxy)-2-oxoethoxy)benzoic acid **5m** (1.00 g, 3.7 mmol), sodium bicarbonate (0.35 g, 4.1 mmol) in tetrahydrofuran (50 mL), fluorenylmethyloxycarbonyl chloride (0.87 g, 3.4 mmol) in chloroform (30 mL). The resulting precipitate was removed *via* filtration to yield the title compound (1.62 g, 3.3 mmol, 98%) as a colourless amorphous solid; (Found C, 68.40; H, 5.50; N, 2.80%. $C_{28}H_{27}NO_7$ requires C, 68.70; H, 5.56; N, 2.86%); d$_H$ (300 MHz, $CDCl_3$) 1.54 (9H, s, $C(CH_3)_3$), 4.34 (1H, t, *J* = 7.1, FHß), 4.55 (2H, d, *J* = 7.1, FHa), 4.70 (2H, s, Ha), 7.36 (2H, t, *J* = 7.2, FHAr4) 7.45 (2H, t, *J* = 7.2, FHAr3), 7.58 (1H, d, *J* = 1.6, H2), 7.71 (2H, d, *J* = 7.3, FHAr5), 7.80-7.86 (3H, m, H6 + FHAr2), 8.18-8.29 (2H, m, NH + H5); d$_C$ (75MHz, $CDCl_3$) 28.1, 47.0, 67.4, 67.6, 83.2, 114.6, 117.8, 120.1, 123.2, 125.2, 125.8, 127.2, 127.8, 134.2, 141.4, 143.7, 146.1, 153.1, 167.9, 171.2; ?$_{max}$/cm^{-1} (solid state) = 3422, 3200-2200 (br), 1740, 1682, 1533, 1182, 760; ESI-HRMS found *m/z* 512.1675 $[M+Na]^+$, $C_{28}H_{27}NNaO_7$ requires 512.1680.

Methyl 3-(2-(methoxymethoxy)ethoxy)-4-nitrobenzoate 3n

Procedure A; Methyl-3-hydroxy-4-nitrobenzoate **2** (3.00 g, 15.2 mmol), potassium carbonate (3.15 g, 22.8 mmol), in dimethylformamide (60 mL), 2-(methoxymethoxy)ethanol (1.97 mL, 16.7 mmol). Work up afforded the title compound (3.65 g, 12.8 mmol, 89 %) as a yellow glassy solid; (Found C, 50.80; H, 5.21; N, 4.75%. $C_{19}H_{21}N_1O_5$ requires C, 50.33; H, 5.30; N, 4.91%); d$_H$ (500 MHz, $CDCl_3$) 3.26 (3H, s, OMe); 3.87-91 (5H, m, Hß + CO_2Me), 4.28 (2H, t, *J* = 5.0, Ha), 4.64 (2H, s, OCH_2O), 7.63 (1H, dd, *J* = 8.5, 1.0, H6), 7.71 (1H, d, *J* = 1, H2), 7.77 (1H, d, *J* = 8.5, H5); d$_C$ (125MHz, $CDCl_3$) 52.8, 55.3, 65.2, 69.4, 96.6, 115.7, 121.6, 125.4, 134.8, 142.6, 151.7, 165.1; ?$_{max}$/cm^{-1} (solid state) = 2993, 2964, 2941, 2890, 1726, 1613, 1591, 1528, 1431, 1369, 1292, 1241, 1114, 1055, 1025; ESI-HRMS found *m/z* 308.074 $[M+Na]^+$, $C_{12}H_{15}NNaO_7$ requires 308.0746

Methyl 4-amino-3-(2-(methoxymethoxy)ethoxy)benzoate 4n

Procedure D; Methyl 3-(2-(methoxymethoxy)ethoxy)-4-nitrobenzoate **3n** (3.50g, 12.3 mmol) in methanol (70 mL), 10% palladium on charcoal (350 mg, 10 wt%) in methanol (20 mL) and hydrogen gas. Work up yielded the title compound (3.01 g, 11.8 mmol, 96%) as a colourless oil; d$_H$ (500 MHz, $CDCl_3$) 3.42 (3H, s, OMe); 3.88 (3H, s, CO_2Me), 3.94 (2H, t, *J* = 4.5, Hß), 4.25 (2H, t, *J* = 4.5, Ha), 4.15 (2H, br. s, NH_2), 4.73 (2H, s, OCH_2O), 6.70 (1H, d, *J* = 8.2, H5), 7.50 (1H, d, *J* = 1.8, H2), 7.58 (1H, dd, *J* = 8.2, 1.8, H6); d$_C$ (125MHz, $CDCl_3$) 51.6, 55.3, 66.1, 68.2, 96.6, 113.1, 113.3, 119.4, 124.6, 141.7, 145.1, 167.2; ?$_{max}$/cm^{-1} (solid state) = 3481, 3366, 2949, 2887, 1704, 1619, 1521, 1442, 1294, 1264, 1217, 1152, 1108, 1037; ESI-HRMS found *m/z* 278.0999 $[M+Na]^+$, $C_{11}H_{15}NNaO_5$ requires 278.1004.

Methyl 4-amino-3-(2-(methoxymethoxy)ethoxy)benzoic acid 5n

Procedure G; Methyl 4-amino-3-(2-(methoxymethoxy)ethoxy)benzoate **4n** (3.00 g, 11.8 mmol) in a 1:1 mixture of methanol : tetrahydrofuran (80 mL), lithium hydroxide (1.00 g, 23.3 mmol) in water (5 mL). Work up yielded the title compound (2.02 g, 8.4 mmol, 76%) as a colourless amorphous solid; d$_H$ (300 MHz, $CDCl_3$) 3.33 (3H, s, OMe), 3.86 (2H, t, *J* = 4.5, Hß), 4.16 (2H, t, *J* = 4.5, Ha), 4.65 (2H, s, OCH_2O), 6.66 (1H, d, *J* = 8.1, H5), 7.43 (1H, d, *J* = 1.7, H2),7.52 (1H, dd, *J* = 8.1, 1.7, H6); d$_C$

(75MHz, $CDCl_3$) 55.3, 66.1, 68.2, 96.5, 113.3, 113.4, 118.2, 125.6, 142.5, 145.0, 172.1; $?_{max}/cm^{-1}$ (solid state) = 3346, 2938, 1711, 1679, 1622, 1595, 1524, 1445, 1302, 1267, 1233, 1148, 1112, 1044; ESI-HRMS found *m/z* 242.1023 $[M+H]^+$, $C_{11}H_{16}NO_5$ requires 242.1028.

4-(((9H-fluoren-9-yl)methoxy)carbonylamino)-3-(2-(methoxymethoxy)ethoxy)benzoic acid 1n

Procedure I; Methyl 4-amino-3-(2-(methoxymethoxy)ethoxy)benzoic acid **5n** (2.20 g, 9.1 mmol), sodium bicarbonate (1.03 g, 27.4 mmol) in tetrahydrofuran (50 mL) and fluorenylmethyloxycarbonyl chloride (3.54 g, 13.7 mmol) in tetrahydrofuran (30 mL). The reaction mixture was concentrated and column chromatography yielded the title compound (2.28 g, 4.9 mmol, 54%) as a colourless amorphous solid; (Found C, 67.40, H:5.40, N: 2.85%. $C_{26}H_{25}NO_7$ requires C, 67.38, H: 5.44, N: 3.02%); d_H (500 MHz, $CDCl_3$) 3.29 (3H, s, O<u>Me</u>), 3.88 (2H, t, *J* = 4.5, Hß), 4.20 (2H, t, *J* = 4.5, Ha), 4.23 (1H, t, *J* = 6.6, FHß), 4.50 (2H, d, *J* = 6.6, FHa), 4.62 (2H, s, OC<u>H</u>$_2$O), 7.26 (2H, t, *J* = 7.5, FHAr4), 7.35 (2H, t, *J* = 7.4, FHAr3), 7.56-7.58 (2H, m, H2 + H5), 7.75 (2H, d, *J* = 7.3, FHAr5), 7.86 (1H, br. s, H6) 7.93 (2H, d, *J* = 7.6, FHAr2), 8.63 (1H, s, N<u>H</u>); d_C (125MHz, $CDCl_3$) 46.4, 54.5, 65.5, 66.1, 68.8, 95.7, 113.5, 119.2, 120.1, 122.8, 125.0, 125.6, 127.0, 127.7, 132.0, 140.7, 143.5, 147.6, 153.0, 166.7; $?_{max}/cm^{-1}$ (solid state) = 3299, 2945, 1709, 1682, 1597, 1539, 1499, 1415, 1300, 1246, 1114, 1088, 1043; ESI-HRMS found *m/z* 464.1673 $[M+H]^+$, $C_{26}H_{26}NO_7$ requires 464.1709.

Methyl 3-(3-(methylthio)propoxy)-4-nitrobenzoate 3o

Procedure B; Methyl-3-hydroxy-4-nitrobenzoate **2** (5.00 g, 25.3 mmol), 3-(methylthio)propan-1-ol (2.87 mL, 30.4 mmol) triphenylphosphine (9.95 g, 38.0 mmol) with diisopropyl azodicarboxylate (7.45 mL, 38.0 mmol) in tetrahydrofuran (150 mL). Column chromatography yielded the title compound (6.51 g, 22.8 mmol, 90%) as pale yellow solid; d_H (300 MHz, $CDCl_3$) 2.13-2.21 (5H, m, Hß, SC<u>H</u>$_3$), 2.78 (2H, t, *J* = 6.9, H?), 4.00 (3H, s, CO_2<u>Me</u>), 4.33 (2H, t, *J* = 5.7, Ha), 7.72 (1H, dd, *J* = 8.4, H6), 7.79 (1H, d, *J* = 1.2, H2), 7.87 (1H, d, *J* = 8.4, H5); d_C (75MHz, $CDCl_3$) 15.5, 28.1, 30.3, 52.8, 67.8, 115.4, 121.4, 125.3, 134.8, 142.4, 151.7, 165.2; $?_{max}/cm^{-1}$ (solid state) = 2953, 2857, 1719, 1609, 1587, 1520, 1433, 1390, 1296, 1237, 1113; ESI-HRMS found *m/z* 308.0563 $[M+Na]^+$, $C_{12}H_{15}NNaO_5S$ requires 308.0569.

Methyl 4-amino3-(3-(methylthio)propoxy)benzoate 4o

Procedure D; Methyl 3-(3-(methylthio)propoxy)-4-nitrobenzoate **3o** (4.00 g, 12.3 mmol) in ethyl acetate (150 mL) and methanol (50 mL), 10% palladium on charcoal (400 mg, 10 wt%) and hydrogen gas. Following work up, column chromatography yielded the title compound (2.62 g, 10.3 mmol, 73%) as a beige amorphous solid; d_H (300 MHz, $CDCl_3$) 2.01-2.10 (5H, m, Hß, SC<u>H</u>$_3$), 2.63 (2H, t, *J* = 7.2, H?), 3.79 (3H, s, CO_2<u>Me</u>), 4.09 (2H, t, *J* = 6.1, Ha), 4.17 (2H, br. s, N<u>H</u>$_2$), 6.60 (1H, d, *J* = 8.1, H5), 7.38 (1H, d, *J* = 1.8, H2), 7.47 (1H, dd, *J* = 8.1, 1.8, H6); d_C (125MHz, $CDCl_3$) 15.5, 28.6, 30.8, 51.6, 66.7, 112.1, 113.1, 119.3, 124.1, 141.2, 145.1, 167.2; ESI-HRMS found *m/z* 256.1002 $[M+H]^+$, $C_{12}H_{18}NO_3S$ requires 256.1007.

4-Amino-3-(3-(methylthio)propoxy)benzoic acid 5o

Procedure F; Methyl 4-amino3-(3-(methylthio)propoxy)benzoate **4o** (2.50 g, 9.8 mmol) in a 1:1 mixture of methanol- tetrahydrofuran (60 mL), 10% aqueous sodium hydroxide (15 mL). Work up yielded the title compound (2.27 g, 9.4 mmol, 96%) as a cream amorphous solid; d_H (500 MHz,$CDCl_3$) 2.12-2.17 (5H, m, Hß, SCH_3), 2.71 (2H, t, *J* = 7.2, H?), 4.19 (2H, t, *J* = 6.1, Ha), 6.70 (1H, d, *J* = 8.3, H5), 7.51 (1H, d, *J* = 1.8, H2), 7.64 (1H, dd, *J* = 8.3, 1.8, H6); d_C (125MHz, $CDCl_3$) 15.7, 28.7, 31.0, 66.9, 112.6, 113.2, 118.4, 125.2, 142.1, 145.2, 171.95; $?_{max}/cm^{-1}$ (solid state) = 3461, 3334, 2869, 2551, 1667, 1615, 1583, 1526, 1446, 1414, 1365, 1300, 1267, 1225, 1148, 1113, 1031; ESI-HRMS found *m/z* 264.0665 $[M+Na]^+$, $C_{11}H_{15}NNaO_3S$ requires 264.0670.

4-(((9H-fluoren-9-yl) methoxy)carbonylamino)-3-(3-(methylthio)propoxy)benzoic acid 1o

Procedure H; 4-Amino-3-(3-(methylthio)propoxy)benzoic acid **5o** (1.70 g, 7.1 mmol), in tetrahydrofuran (50 mL) and fluorenylmethyloxycarbonyl chloride (2.72 g, 10.6 mmol) in tetrahydrofuran (30 mL). The crude material obtained after concentration was suspended in hexane and filtered (3 times). The solid obtained was then washed with methanol to get the title compound (2.94g, 6.3 mmol, 90%) as a colourless amorphous solid; (Found C, 67.45.; H, 5.35; N, 2.95; S 6.85%. $C_{26}H_{25}NO_5S$ requires C, 67.37; H, 5.44; N, 3.02; S, 6.92%); d_H (500 MHz,$CDCl_3$) 2.19 (3H, s, SCH_3), 2.21 (2H, p, *J* = 6.4, Hß), 2.74 (2H, t, *J* = 6.7, H?), 4.26 (2H, t, *J* = 6.1, Ha), 4.33 (1H, t, *J* = 6.8, FHß), 4.56 (2H, d, *J* = 6.8, FHa), 7.35 (2H, t, *J* = 7.5, FHAr4), 7.44 (2H, t, *J* = 7.5, FHAr3), 7.61 (1H, d, *J* = 1.7, H2), 7.65 (2H, d, *J* = 7.5, FHAr5), 7.70 (1H, br. s, H6), 7.77 (1H, d, *J* = 8.1, H5), 7.80 (2H, FHAr2), 8.18 (1H, br. s, NH); d_C (125MHz, $CDCl_3$) 15.9, 28.3, 31.2, 47.1, 67.4, 67.8, 112.1, 117.3, 120.1, 123.2, 124.5, 125.0, 127.2, 127.9, 133.0, 141.4, 143.7, 146.3, 153.0, 177.6; $?_{max}/cm^{-1}$ (solid state) = 3311, 2954, 2915, 1712, 1682, 1597, 1535, 1499, 1416, 1338, 1300, 1281, 1245, 1227, 1104, 1088, 1046, 1032; ESI-HRMS found *m/z* 486.1341 $[M+Na]^+$, $C_{26}H_{25}NNaO_5S$ requires 486.1351.

Methyl 4-nitro-3-(2-oxo-2-(tritylamino)ethoxy)benzoate 3p

Procedure A; Methyl-3-hydroxy-4-nitrobenzoate **2** (1.17 g, 5.9 mmol), potassium carbonate (2.46 g, 17.8 mmol), in dimethylformamide (40 mL), 2-bromo-N-tritylacetamide (2.25 g, 5.9 mmol). Work-up afforded the title compound (2.73 g, 5.5 mmol, 93 %) as a yellow amorphous solid; d_H (500 MHz, $CDCl_3$) 3.90 (3H, s, CO_2Me), 4.60 (2H, s, Ha), 7.18-7.26 (15H, m, $C(C_6H_5)_3$), 7.66 (1H, d, *J* = 1.4, H2), 7.72 (1H, dd, *J* = 8.5, 1.4, H6), 7.95 (1H, d, *J* = 8.5, H5), 8.06 (1H, s, NH); d_C (125MHz, $CDCl_3$) 53.0, 68.3, 70.7, 115.6, 122.9, 126.4, 127.2, 128.1, 128.7, 135.8, 144.2, 150.2, 164.6, 165.0; $?_{max}/cm^{-1}$ (solid state) = 3408, 3056, 1958, 1728, 1697, 1522, 1234, 700; ESI-HRMS found *m/z* 519.1525 $[M+Na]^+$, $C_{29}H_{24}N_2NaO_6$ requires 519.1527.

Methyl 4-amino-3-(2-oxo-2-(tritylamino)ethoxy)benzoate 4p

Procedure D; Methyl 4-nitro-3-(2-oxo-2-(tritylamino)ethoxy)benzoate **3p** (2.70 g, 5.4 mmol) in ethyl acetate (60 mL), 10% palladium on charcoal (270 mg, 10 wt%) and hydrogen gas. Work up yielded the title compound (2.45 g, 5.3 mmol, 96%) as a beige solid; (Found C, 74.15; H, 5.75; N, 5.65%. $C_{29}H_{26}N_2O_4$ requires C, 74.66; H, 5.64; N, 6.00%); d_H (500 MHz, $CDCl_3$) 3.80 (3H, s, CO_2Me), 4.45

(2H, s, Ha), 6.71 (1H, d, *J* = 8.2, H5), 7.09-7.10 (6H, m, ArH), 7.15-7.22 (9H, m, ArH), 7.45 (1H, d, *J* = 1.4, H2), 7.53-7.55 (2H, m, H6 + NH); d_C (125MHz, $CDCl_3$) 51.9, 68.7, 70.4, 113.7, 114.6, 120.4, 125.6, 127.2, 128.1, 128.6, 140.6, 143.9, 144.3, 166.7, 166.7; $?_{max}/cm^{-1}$ (solid state) = 3488, 3370, 3033, 1966, 1691, 1619, 1520, 1434, 1258, 698; ESI-HRMS found *m/z* 489.1799 $[M+Na]^+$, $C_{29}H_{26}N_2NaO_6$ requires 489.1785.

4-Amino-3-(2-oxo-2-(tritylamino)ethoxy)benzoic acid 5p

Procedure F; Methyl 4-amino-3-(2-oxo-2-(tritylamino)ethoxy)benzoate **4p** (2.40 g, 5.1 mmol) in a 1:1 mixture of methanol-tetrahydrofuran (60 mL), 10% aqueous sodium hydroxide (15 mL). Work up yielded the title compound (1.46 g, 3.2 mmol, 63%) as a colourless amorphous solid; d_H (500 MHz, DMSO-d_6) 4.71 (2H, s, Ha), 5.53 (2H, br. s, NH_2) 6.64 (1H, d, *J* = 8.5, H5), 7.15-7.26 (15H, m, $C(C_6H_5)_3$),7.38-7.39 (2H, m, H2 + H6) 8.69 (1H, brs, NH); d_C (125MHz, $CDCl_3$) 69.2, 71.7, 93.4, 115.0, 115.5, 125.8, 128.1, 128.7, 129.0, 130.0, 141.2, 145.6, 145.7, 170.3, 175.6; ESI-HRMS found *m/z* 451.1667 $[M-H]^-$, $C_{28}H_{23}N_2O_4$ requires 451.1663.

4-((((9H-fluoren-9-yl)methoxy)carbonyl)amino)-3-(2-oxo-2-(tritylamino)ethoxy)benzoic acid 1p

Procedure I; 4-Amino-3-(2-oxo-2-(tritylamino)ethoxy)benzoic acid **5p** (1.40 g, 3.1 mmol), sodium bicarbonate (350 mg, 9.3 mmol) in tetrahydrofuran (50 mL) and fluorenylmethyloxycarbonyl chloride (1.20 g, 4.6 mmol) in tetrahydrofuran (10 mL). Work up yielded the title compound (1.52 g, 2.3 mmol, 73%) as a colourless amorphous solid; d_H (500 MHz, DMSO-d_6) 4.29 (1H, t, *J* = 6.6, FHß), 4.45 (2H, d, *J* = 6.6, FHa), 4.85 (2H, s, Ha), 7.13-7.14 (6H, m, ArH), 7.14-7.23 (9H, m, ArH), 7.29 (2H, t, *J* = 7.4, FHAr4), 7.41 (2H, t, *J* = 7.4, FHAr3), 7.55-7.56 (2H, m, H2 + H6), 7.73 (2H, d, *J* = 7.6, FHAr5), 7.78 (1H, br. s, H5), 7.90 (2H, d, *J* = 7.6, FHAr2), 8.83 (1H, s, NH), 9.05 (1H, s, FNH); d_C (125MHz, DMSO-d_6) 46.7, 66.3, 67.6, 69.4, 113.4,119.8, 120.1, 123.0, 125.2, 125.7, 126.5, 127.1, 127.5, 127.7, 128.4, 132.0, 140.7, 143.6, 144.4, 147.3, 153.3, 166.8, 167.1; $?_{max}/cm^{-1}$ (solid state) = 3397, 3261, 3059, 1951, 1736, 1668, 1530, 1221, 1190, 1046, 739, 699; ESI-HRMS found *m/z* 697.2284 $[M+Na]^+$, $C_{43}H_{34}N_2NaO_6$ requires 697.2309.

Methyl 3-(2-((tert-butoxycarbonyl)amino)ethoxy)-4-nitrobenzoate 3q

Procedure B; Methyl-3-hydroxy-4-nitrobenzoate **2** (3.00 g, 15.2 mmol), tert-butyl (2-hydroxyethyl)carbamate (2.59 mL, 16.7 mmol), triphenylphosphine (6.00 g, 22.8 mmol) with diisopropyl azodicarboxylate (4.48 mL, 22.8 mmol) in tetrahydrofuran (90 mL). Column chromatography yielded the title compound (4.14 g, 12.2 mmol, 80%) as a pale yellow solid; (Found C, 53.00; H5.90; N, 8.20%. $C_{15}H_{20}N_2O_7$ requires C, 52.94; H, 5.92; N, 8.23%); d_H (500 MHz, $CDCl_3$); 1.45 (9H, s, $C(CH_3)$), 3.61 (2H, m, Hß), 3.96 (3H, s, CO_2Me), 4.24 (2H, t, *J* = 4.8, Ha), 5.09 (1H, br. s, NH), 7.71-7.73 (2H, m, H2 + H6), 7.87 (1H, d, *J* = 8.3, H5); d_C (125MHz, $CDCl_3$); 28.4, 39.7, 52.9, 69.4, 79.8, 115.7, 121.8, 125.5, 135.1, 142.3, 151.6, 155.9, 165.0; $?_{max}/cm^{-1}$ (solid state) = 3375, 2973, 1724, 1703, 1605, 1518, 1494, 1454, 1441, 1392, 1359, 1304, 1280, 1246, 1171, 1117, 1089, 1072; ESI-HRMS found *m/z* 363.1160 $[M+Na]^+$, $C_{15}H_{20}N_2NaO_5$ requires 363.1168.

Methyl 4-amino-3-(2-((tert-butoxycarbonyl)amino)ethoxy)benzoate 4q

Procedure D; Methyl 3-(2-((tert-butoxycarbonyl)amino)ethoxy)-4-nitrobenzoate **3q** (2.00 g, 5.9 mmol) in 1:1 ethyl acetate-methanol (40 mL), 10% palladium on charcoal (200 mg, 10 wt%) and hydrogen gas. Work up yielded the title compound (1.6 g, 5.4 mmol, 91%) as a beige solid; d_H (500 MHz, $CDCl_3$) 1.49 (9H, s, C(C$\underline{H}_3$)), 3.59 (2H, m, Hß), 3.86 (3H, s, $CO_2\underline{Me}$), 4.12 (2H, t, *J* = 5.1, Ha), 4.28 (2H, br. s, N$\underline{H}_2$), 4.91 (1H, br. s, N$\underline{H}$), 6.68 (1H, d, *J* = 8.2, H5), 7.43 (1H, s, H2), 7.56 (1H, d, *J* = 8.2, H6); d_C (75MHz, $CDCl_3$); 28.4, 40.1, 51.7, 67.97, 79.60, 113.2, 113.3, 119.4, 124.4, 141.2, 144.9, 156.0, 167.2; ?$_{max}$/cm^{-1} (solid state) = 3439, 3387, 3063, 2948, 1712, 1696, 1670, 1601, 1523, 1482, 1422, 1352, 1267, 1251, 1207, 1122, 1017; ESI-HRMS found *m/z* 333.1421 $[M+Na]^+$, $C_{15}H_{22}N_2NaO_5$ requires 333.1426.

4-Amino-3-(2-((tert-butoxycarbonyl)amino)ethoxy)benzoic acid 5q

Procedure G; Methyl 4-amino-3-(2-((tert-butoxycarbonyl)amino)ethoxy)benzoate **4q** (1.00 g, 3.2 mmol) in a 1:1 mixture of methanol- tetrahydrofuran (20 mL), lithium hydroxide (270 mg, 6.5 mmol) in water (5 mL). Work up yielded the title compound (0.92 g, 3.1 mmol, 96%) as a colourless amorphous solid; d_H (500 MHz, MeOD-d_4) 1.47 (9H, s, C(C$\underline{H}_3$)), 3.51 (2H, t, *J* = 5.1, Hß), 4.05 (2H, t, *J* = 5.1, Ha), 6.71 (1H, d, *J* = 8.2, H5), 7.42 (1H, s, H2), 7.51 (1H, d, *J* = 8.2, H6); d_C (125MHz, MeOD-d_4); 28.7, 41.0, 68.9, 80.2, 113.3, 113.9, 119.3, 125.9, 144.4, 146.4, 158.7, 170.6; ?$_{max}$/cm^{-1} (solid state) = 3347, 2972, 1675, 16171584, 1518, 1444, 1402, 1368, 1293, 1271, 1222, 1161, 1123, 1057; ESI-HRMS found *m/z* 297.1445 $[M+H]^+$, $C_{14}H_{21}N_2O_5$ requires 297.1450.

4-((((9H-fluoren-9-yl)methoxy)carbonyl)amino)-3-(2-((tert-butoxycarbonyl)amino) ethoxy)benzoic acid 1q

Procedure I; 4-Amino-3-(2-((tert-butoxycarbonyl)amino)ethoxy)benzoic acid **5q** (1.40 g, 3.1 mmol), sodium bicarbonate (1.19 g, 14.2 mmol) in tetrahydrofuran (30 mL) and fluorenylmethyloxycarbonyl chloride (1.83 g, 7.1 mmol) in tetrahydrofuran (20 mL). The reaction mixture was concentrated and column chromatography yielded the title compound (1.83 g, 3.5 mmol, 75%) as a colourless amorphous solid; (Found C, 66.95; H, 5.80; N, 5.25%. $C_{29}H_{30}N_2O_7$ requires C, 67.17; H, 5.83; N, 5.40%); d_H (500 MHz, DMSO-d_6) 1.39 (9H, s, C(C$\underline{H}_3$)), 3.42 (2H, m, Hß), 4.03 (2H, m, Ha), 4.36 (1H, t, *J* = 7.0, FHß), 4.50 (2H, d, *J* = 7.0, FHa), 7.31-7.37 (3H, m, H2 + FHAr4), 7.42-7.45 (3H, m, H6 + FHAr3), 7.50 (1H, d, *J* = 8.4, H5), 7.78 (2H, d, *J* = 7.5, FHAr5), 7.83 (1H, br. s, N$\underline{H}$), 7.93 (2H, d, *J* = 7.5, FHAr2), 9.01 (1H, s, FN$\underline{H}$); d_C (125MHz, DMSO-d_6) 27.95, 46.2, 66.2, 68.2, 77.7, 111.3, 118.6, 119.9, 122.1, 124.8, 125.1, 126.9, 127.5, 131.3, 140.5, 143.4, 147.2, 153.1, 155.6, 166.6; ?$_{max}$/cm^{-1} (solid state) = 3423, 3361, 2986, 2947, 1741, 1682, 1605, 1532, 1489, 1440, 1347, 1297, 1248, 1227, 1198, 1132, 1053; ESI-HRMS found *m/z* 541.1945 $[M+Na]^+$, $C_{29}H_{30}N_2NaO_7$ requires 541.1951.

3-((5-((tert-butoxycarbonyl)amino)pentyl)oxy)-4-nitrobenzoic acid 4r

Methyl 3-((5-((tert-butoxycarbonyl)amino)pentyl)oxy)-4-nitrobenzoate **3r** was obtained from methyl-3-hydroxy-4-nitrobenzoate **2** by *procedure B* without further purification / isolation. *Procedure F*; methyl 3-((5-((tert-butoxycarbonyl)amino)pentyl)oxy)-4-nitrobenzoate **3r** (5.80 g, 15.2 mmol) in a

1:1 mixture of methanol-tetrahydrofuran (150 mL) and 10% aqueous sodium hydroxide (30 mL). Work up yielded the title compound (4.95 g, 13.4 mmol, 89% over two steps) as a cream amorphous solid; d$_H$ (500 MHz, $CDCl_3$) 1.45-1.56 (13H, m, H?, Hd + C(C$\underline{H}_3$)), 1.87 (2H, m, Hß), 3.16 (2H, m, He), 4.16 (2H, t, *J* = 6.1, Ha), 7.72 (1H, d, *J* = 7.7, H6), 7.75 (1H, s, H2), 7.81 (1H, d, *J* = 7.7, H5); d$_C$ (125MHz, $CDCl_3$) 23.1, 28.4, 29.6, 40.4, 41.5, 69.7, 79.5, 115.8, 121.8, 125.2, 134.4, 142.9, 156.3, 158.3, 168.4; ?$_{max}$/cm^{-1} (solid state) = 3377, 2980, 2944, 1693, 1521, 1308, 1249, 1177; ESI-HRMS found *m/z* 391.1495 $[M+Na]^+$ $C_{17}H_{24}N_2NaO_7$ requires 391.1481.

4-amino-3-((5-((tert-butoxycarbonyl)amino)pentyl)oxy)benzoic acid 5r

Procedure D; 3-((5-((tert-butoxycarbonyl)amino)pentyl)oxy)-4-nitrobenzoic acid **4r** (4.90 g, 13.4 mmol) a 1:2 mixture of ethyl acetate-methanol (90 mL), 10% palladium on charcoal (490 mg, 10 wt%) and hydrogen gas. Work up yielded the title compound (4.50 g, 13.3 mmol, 91%) as a beige amorphous solid; (Found C, 59.00; H, 7.70; N, 8.60%. $C_{17}H_{25}N_2O_7$ requires C, 60.34; H, 7.74; N, 8.28%); d$_H$ (500 MHz, MeOD-d$_4$) 1.43 (9H, s, C(C$\underline{H}_3$)), 1.53 (4H, m, H? + Hd), 1.85 (2H, m, Hß), 3.07 (2H, t, *J* = 6.2, He), 4.04 (2H, t, *J* = 6.3, Ha), 6.69 (1H, d, *J* = 8.2, H5), 7.41 (1H, s, H2), 7.48 (1H, d, *J* = 8.2, H6); d$_C$ (125MHz, MeOD-d$_4$) 28.5, 28.8, 29.2, 30.1, 41.3, 69.3, 79.9, 113.3, 113.4, 119.6, 125.5, 125.6, 144.2, 146.7, 151.0, 158.6, 170.8; ?$_{max}$/cm^{-1} (solid state) = 3492, 3347, 2940, 1703, 1690, 1657, 1620, 1588, 1576, 1518, 1417, 1367, 1308, 1268, 1237, 1169, 1153, 1029; ESI-HRMS found *m/z* 337.1769 $[M-H]^-$ $C_{17}H_{25}N_2O_7$ requires 337.1763.

4-((((9H-fluoren-9-yl)methoxy)carbonyl)amino)-3-((5-((tert-butoxycarbonyl) amino) pentyl)oxy)benzoic acid 1r

Procedure I; 4-amino-3-((5-((tert-butoxycarbonyl)amino)pentyl)oxy)benzoic acid **5r** (4.00 g, 11.8 mmol), sodium bicarbonate (2.98 g, 34.2 mmol) in tetrahydrofuran (80 mL) and fluorenylmethyloxycarbonyl chloride (4.58 g, 17.8 mmol) in tetrahydrofuran (40 mL). Precipitation of the product *via* hexane yielded the title compound (5.50 g, 9.8 mmol, 79%) as a colourless amorphous solid; d$_H$ (500 MHz, $CDCl_3$) 1.44 (9H, s, C(C$\underline{H}_3$)), 1.54 (2H, m, H?), 1.60 (2H, m, Hd), 1.91 (2H, m, Hß), 3.17 (2H, m, He), 4.13 (2H, t, *J* = 6.6, Ha), 3.34 (1H, t, *J* = 6.9, FHß), 4.55 (3H, m, N$\underline{H}$ + FHa), 7.34 (2H, t, *J* = 7.4, FHAr4), 7.43 (2H, t, *J* = 7.5, FHAr3), 7.49 (1H, br. s, H6), 7.57 (1H, d, *J* = 1.5, H2), 7.64 (2H, d, *J* = 7.5, FHAr5), 7.75 (1H, d, *J* = 8.3, H5), 7.80 (2H, d, *J* = 7.5, FHAr2), 8.14 (1H, br. s, FN$\underline{H}$); d$_C$ (125MHz, $CDCl_3$) 14.2, 21.1, 23.3, 28.4, 28.7, 29.9, 47.1, 60.4, 67.4, 68.8, 94.9, 112.0, 117.3, 120.1, 124.2, 125.0, 127.2, 127.9, 141.4, 143.7, 146.4, 149.3, 153.0, 156.0, 171.2; ?$_{max}$/cm^{-1} (solid state) =3334, 2937, 1706, 1672, 1595, 1531, 1496, 1431, 1281, 1243, 1214, 1173, 1085, 1045; ESI-HRMS found *m/z* 559.2460 $[M-H]^-$ $C_{32}H_{35}N_2O_7$ requires 559.2450.

4-((((9H-fluoren-9-yl)methoxy)carbonyl)amino)-3-methylbenzoic acid 1s

Procedure H; 4-Amino-3-methylbenzoic acid **6** (3.00 g, 19.9 mmol) was dissolved in tetrahydrofuran (100 mL), fluorenylmethyloxycarbonyl chloride (7.70 g, 29.8 mmol) in tetrahydrofuran (50 mL). Work up yielded the title compound (7.12 g, 19.1 mmol, 96%) as a colourless amorphous solid; (Found C, 73.90; H, 5.10; N, 3.65%. $C_{23}H_{19}NO_4$ requires C, 73.98; H, 5.13; N, 3.75%); d$_H$ (300 MHz,

DMSO-d_6) 2.26 (3H, s, ArCH_3), 4.32 (1H, t, *J* = 7.5, FHß), 4.48 (2H, d, *J* = 7.5, FHa), 7.34 (2H, t, *J* = 7.5, FHAr4), 7.41-7.47 (3H, m, H2 + FHAr3) 7.64-7.76 (4H, m, H6, H5 + FHAr5), 7.90 (2H, d, *J* = 7.5, FHAr2), 9.21 (2H, s, NH); d_C (75MHz, DMSO-d_6) 17.8, 46.6, 65.9, 120.2, 123.3, 125.2, 126.4, 127.1, 127.4, 127.7, 130.6, 131.5, 140.6, 140.8, 143.7, 153.9, 167.0; $?_{max}$/cm^{-1} (solid state) = 3271, 2831, 1701, 1685, 1528, 1253, 736; ESI-HRMS found *m/z* 374.1390 $[M+H]^+$, $C_{23}H_{20}NO_4$ requires 374.1387.

Characterization of Oligomers

NH_2-[*O*-*i*Pr-(3-HABA)]-[*O*-*i*Pr-(3-HABA)]-[*O*-*i*Pr-(3-HABA)]-Gly-CO_2H 7

d_H (500 MHz, DMSO-d_6) 1.32 (6H, d, *J* = 6.0, 1-Hß), 1.35 (6H, d, *J* = 6.0, 3-Hß), 1.39 (6H, d, *J* = 6.0, 2-Hß), 3.93 (2H, d, *J* = 4.5, 4-Ha), 4.60 (1H, sept, *J* = 6.0, 1-Ha), 4.72 (1H, sept, *J* = 6.0, 1.9, 3-Ha), 4.79 (1H, sept, *J* = 6.0, 1.5, 2-Ha), 5.43 (2H, br. s, 1-NH_2), 6.72 (1H, d, *J* = 8.1, 1-H5), 7.32-7.35 (2H, m, 1-H2, 1-H6), 7.51-7.61 (4H, m, 2-H2, 2-H6, 3-H2, 3-H6), 8.12 (1H, app t, *J* = 8.1, 3-H5), 8.29 (1H, app t, *J* = 7.5, 2-H5), 8.81 (1H, t, *J* = 5.8, 4-NH), 8.94 (1H, s, 3-NH), 9.27 (1H, s, 2-NH); d_C (125MHz, DMSO-d_6) 21.8, 21.8, 21.9, 41.2, 70.36, 71.39, 71.41 112.2, 122.6, 112.8, 119.9, 120.0, 120.3, 120.9, 121.4, 121.6, 129.2, 129.9, 131.1, 132.2, 143.24, 146.9, 147.0, 147.8, 147.9, 164.2, 164.4, 165.8, 171.3; ESI-HRMS found *m/z* 629.2558 $[M+Na]^+$, $C_{32}H_{38}N_4O_8Na$ requires 629.2582.

NH_2-[*O*-Bn-(3-HABA)]-[*O*-Bn-(3-HABA)]-[*O*-Bn-(3-HABA)]-Gly-CO_2H 8

d_H (300 MHz, DMSO-d_6) 3.95 (2H, d, *J* = 5.6, 4-Ha), 5.14 (2H, s, 1-Ha), 5.26 (2H, s, 2-Ha), 5.29 (2H, s, 3-Ha), 6.78 (1H, d, *J* = 8.2, 1-H5) 7.26-7.64 (19H, m, ArCH), 7.73 (2H, m, 3-HAr2), 8.00 (1H, d, *J* = 8.3, 2-H5), 8.15 (1, d, *J* = 8.3, 3-H5), 8.88 (1H, t, *J* = 5.6, 4-NH), 9.20 (1H, s, 2-NH), 9.57 (1H, s, 3-NH); d_C (125MHz, DMSO-d_6) 41.2, 69.5, 70.2, 70.3, 111.1, 111.6, 111.8, 113.2, 120.0, 120.4, 121.2, 121.6, 122.7, 127.4, 127.5, 127.6, 127.8, 127.9, 128.0, 128.4, 129.7, 130.3, 130.5, 131.4, 136.6, 136.8, 137.0, 144.7, 148.7, 149.6, 164.3, 164.5, 165.8, 171.4; ESI-HRMS found *m/z* 751.2785 $[M+H]^+$, $C_{44}H_{39}N_4O_8$ requires 751.2762.

NH_2-[*O*-*i*Pr-(3-HABA)]-[*O*-2-CH_2-Nap-(3-HABA)]-[*O*-Bn-(3-HABA)]-Gly-CO_2H 9

d_H (500 MHz, DMSO-d_6) 1.13 (6H, d, *J* = 6.0, 1-Hß), 3.94 (2H, d, *J* = 5.8, 4-Ha), 4.40 (1H, sept, *J* = 6.0, 1-Ha), 5.27 (2H, s, 3-Ha), 5.39 (2H, s, 2-Ha), 6.71 (1H, d, *J* = 8.1, 1-H5), 7.27 (1H, t, *J* = 7.3, 3-HAr4), 7.31 (1H, d, *J* = 1.7, 1-H2), 7.34-7.38 (3H, m, 1-H6 + 3-HAr3), 7.52-7.57 (5H, m, 3-HAr2 + ArCH), 7.61 (1H, dd, *J* = 8.4, 1.6, 2-H2), 7.68-7.70 (2H, m, 2-H2 + 2-H6), 7.78 (1H, d, *J* = 1.5, 3-H2), 7.87 (1H, m, ArCH), 7.92-7.95 (2H, m, ArCH), 8.01 (1H, d, *J* = 8.1, 3-H5), 8.06 (1H, s, HAr2), 8.18 (1H, d, *J* = 8.3, 2-H5), 8.83 (1H, t, *J* = 5.8, 4-NH), 9.18 (1H, s, 2-NH), 9.54 (1H, s, 3-NH); d_C (125 MHz, DMSO-d_6) 21.6, 41.2, 70.2, 70.4, 70.4, 111.5, 111.7, 112.5, 113.5, 120.0, 120.4, 121.4, 121.6, 121.7, 122.8, 125.5, 126.3, 126.3, 126.4, 127.4, 127.4, 127.6, 127.7, 127.9, 128.1, 128.4, 128.4, 129.8, 130.3, 130.5, 131.4, 132.6, 132.8, 134.1, 136.7, 141.9, 143.7, 148.9, 149.7, 164.4, 164.6, 165.9, 171.3; ESI-HRMS found *m/z* 751.2803 $[M-H]^-$, $C_{44}H_{39}N_4O_8$ requires 751.2773.

NH_2-[*O*-Bn-(3-HABA)]-[*O*-2-CH_2-Nap-(3-HABA)]-[*O*-*i*Pr-(3-HABA)]-Gly-CO_2H 10

d_H (300 MHz,DMSO-d_6) 1.35 (6H, d, *J* = 6.0, 3-Hß), 3.95 (2H, d, *J* = 5.8, 4-Ha), 4.73 (1H, sept, *J* = 6.0, 3-Ha), 5.11 (2H, s, 2-Ha), 5.52 (2H, s, 1-Ha), 6.76 (1H, d, *J* = 8.1, 1-H5), 7.31-7.41 (3H, m, ArCH), 7.43-7.46 (3H, m, 1-H6 + ArCH), 7.50-7.56 (4H, m, 3-H6 + 1-H2 + ArCH), 7.60 (1H, d, *J* = 1.6, 3-H2), 7.63 (1H, dd, *J* = 8.3, 1.6, 2-H6), 7.73 (1H, dd, *J* = 8.3, 1.6, ArCH), 7.79 (1H, d, *J* = 1.8, 2-H2), 7.88 (2H, m, ArCH), 7.94 (1H, d, *J* = 8.3, 2-HAr2), 8.10 (2H, m, 3-H5 + 2-HAr8), 8.21 (1H, d, *J* = 8.3, 2-H5), 8.84 (1H, t, *J* = 6.0, 4-NH), 9.27 (1H, s, 2-NH), 9.34 (1H, s, 3-NH); d_C (125 MHz, DMSO-d_6) 21.8, 41.2, 69.5, 70.4, 71.5, 111.2, 111.8, 112.6, 113.1, 119.9, 120.2, 121.5, 121.6, 121.8, 125.3, 125.9, 126.2, 126.4, 127.4, 127.6, 127.7, 128.1, 128.4, 129.9, 130.1, 131.1, 131.5, 132.6, 132.8, 134.4, 136.9, 144.6, 148.1, 148.9, 164.2, 164.6, 165.9, 171.4; ESI-HRMS found *m/z* 751.2774 $[M-H]^-$, $C_{44}H_{39}N_4O_8$ requires 751.2773.

NH_2-[*O*-Bn-(3-HABA)]-[*O*-*p*CF_3-Bn-(3-HABA)]-[*O*-*i*Pr-(3-HABA)]-Gly-CO_2H 11

d_H (500MHz, DMSO-d_6) 1.33 (6H, d, *J* = 6, 3-Hß), 3.93 (2H, d, *J* = 4.9, 4-Ha), 4.70 (1H, sept, *J* = 6.2, 3-Ha), 5.14 (2H, s, 1-Ha), 5.45 (2H, s, 2-Ha), 5.51 (2H, br. s, 1-NH_2), 6.71 (1H, d, *J* = 8.1, 1-H5), 7.32 (1H, t, *J* = 7, 1-HAr4), 7.36-7.41 (4H, m, 1-H2, 1-H6, 1-HAr2), 7.47-7.53 (3H, m, 1-HAr3, 3-H6), 7.58 (1H, s, 3-H2), 7.61 (1H, dd, *J* = 8.4, 1.6, 2-H6), 7.69 (1H, s, 2-H2), 7.73 (2H, d, *J* =8.3, 2-HAr3), 7.79 (2H, d, *J* = 8.1, 2-HAr2), 8.07 (1H, d, *J* = 8.1, 3-H5), 8.17 (1H, d, *J* = 8.3, 2-H5), 8.81 (1H, t, *J* = 4.9, 4-NH), 9.22 (1H, s, 2-NH), 9.29 (1H, s, 3-NH); d_C (125MHz, DMSO-d_6) 21.8, 41.2, 69.2, 69.5, 71.4, 111.2, 111.6, 112.5, 112.6, 119.9, 120.2, 120.8, 121.9, 121.9, 124.2 (*J* = 272.3) 125.3, 127.4, 127.7, 128.4, 128.4 (*J* = 31.7), 129.9, 130.1, 131.1, 131.5, 137.0, 141.7, 142.4, 144.3, 148.1, 148.7, 164.2, 164.7, 165.8, 171.4; ESI-HRMS found *m/z* 771.2633 $[M+H]^+$, $C_{41}H_{38}F_3N_4O_8$ requires 771.2636.

NH_2-[*O*-*p*Cl-Bn-(3-HABA)]-[*O*-*m*CF_3-Bn-(3-HABA)]-[*O*-*i*Pr-(3-HABA)]-Gly-CO_2H 12

d_H (500MHz, DMSO-d_6) 1.34 (6H, d, *J* = 6.2, 3-Hß), 3.93 (2H, d, *J* = 5.8, 4-Ha), 4.71 (1H, sept, *J* = 6, 3-Ha), 5.13 (2H, s, 1-Ha), 5.41 (2H, s, 2-Ha), 6.71 (1H, d, *J* = 8.1, 1-H5), 7.39 (1H, dd, *J* = 8.0, 1.5, 1-H6), 7.44 (2H, d, *J* = 8.5, 1-HAr2), 7.48 (1H, d, *J* = 1.5, 1-H2), 7.51-7.54 (3H, m, 1-HAr3, 3-H6), 7.58 (1H, d, *J* = 1.5, 3-H2), 7.59-7.63 (2H, m, 2-H6, 2-HAr5), 7.67 (1H, d, *J* = 7.0, 2-HAr6), 7.72 (1H, d, *J* = 1.5, 2-H2), 7.85 (1H, d, *J* = 7.0, 2-HAr4), 8.00 (1H, s, HAr2), 8.07 (1H, d, *J* = 8.3, 3-H5), 8.14 (1H, d, *J* = 8.3, 2-H5), 8.81 (1H, t, *J* = 5.8, 4-NH), 9.27 (1H, s, 2-NH), 9.31 (1H, s, 3-NH); d_C (125MHz, DMSO-d_6) 22.3, 41.8, 69.2, 69.8, 72.0, 112.0, 112.2, 113.2, 113.3, 120.7, 121.4, 121.7, 122.1, 122.4, 122.6, 124.4, 124.7 (J = 272.4), 125.1, 128.9, 129.8, 129.8 (*J* = 32), 130.0, 130.6, 130.6, 131.6, 131.8, 132.0, 132.8, 136.6, 138.8, 142.3, 144.9, 148.6, 149.5, 164.7, 165.1, 166.4, 171.9; ESI-HRMS found *m/z* 803.2133$[M-H]^-$, $C_{41}H_{35}ClF_3N_4O_8$ requires 803.2101.

NH_2-[*O*-Bn-(3-HABA)]-[*O*-*p*Cl-Bn-(3-HABA)]-[*O*-*i*Pr-(3-HABA)]-Gly-CO_2H 13

d_H (500 MHz, DMSO-d_6) 1.37 (6H, d, *J* = 6.0, 3-Hß), 3.95 (2H, d, *J* = 5.8, 4-Ha), 4.74 (1H, sept, *J* = 6.0, 3-Ha), 5.16 (2H, s, 1-Ha), 5.35 (2H, s, 2-Ha), 6.76 (1H, d, *J* = 8.3, 1-H5), 7.34 (1H, t, *J* = 7.5, 1-HAr4), 7.39-7.46 (5H, m, 1-H6, 1-HAr3, 2-HAr3), 7.49-7.56 (4H, m, 1-H2, 1-HAr2, 3-H6), 7.60-7.63 (4H, m, 2-H6, 2-HAr2 + 3-H2), 7.71 (1H, d, *J* = 1.7, 2-H2), 8.10 (1H, d, *J* = 8.3, 2-H5), 8.19 (1H, d,

J = 8.3, 3-H5), 8.83 (1H, t, *J* = 6.0, 4-NH), 9.19 (1H, s, 2-NH), 9.31 (1H, s, 3-NH); d$_C$ (125MHz, DMSO-d$_6$) 21.8, 41.2, 68.3, 69.5, 71.4, 111.2, 111.7, 112.6, 112.8, 119.9, 120.2, 121.3, 121.6, 121.7, 121.9, 127.5, 127.8, 128.4, 128.4, 129.3, 129.8, 130.1, 131.1, 131.5, 132.6, 135.8, 137.0, 144.5, 148.1, 148.7, 164.2, 164.6, 165.8, 171.4; ESI-HRMS found *m/z* 735.2243 $[M-H]^-$, $C_{40}H_{36}ClNO_6$ requires 735.2227.

NH_2-[*O*-*i*Pr-(3-HABA)]-[3-Me-ABA]-[*O*-*i*Pr-(3-HABA)]-Gly-CO_2H 14

d$_H$ (500 MHz, DMSO-d$_6$) 1.3 (6H, d, *J* = 6.0, 1-Hß), 1.36 (6H, d, *J* = 6.0, 3-Hß), 2.32 (3H, s, 2-Ha), 3.93 (2H, d, *J* = 5.8, 4-Ha), 4.58 (1H, sept, *J* = 6.0, 1-Ha), 4.72 (1H, sept, *J* = 6.0, 3-Ha), 6.70 (1H, d, *J* = 8.3, 1-H5), 7.44-7.45 (2H, m, 1-H2 + 1-H6), 7.52 (1H, d, *J* = 8, 3-H6), 7.58-7.59 (2H, m, 2-H2 + 2-H6), 7.76 (1H, d, *J* = 7.5, 2-H5), 7.82 (1H, s, 3-H2), 8.10 (1H, d, *J* = 8, 3-H5), 8.81 (1H, t, 5.8, 4-NH), 9.26 (1H, s, 3-NH), 9.52 (1H, s, 2-NH); d$_C$ (125MHz, DMSO-d$_6$) 18.21, 21.8, 22.0, 41.2, 70.5, 71.4, 112.6, 113.5, 119.9, 121.1, 121.6, 122.0, 125.0, 125.6, 129.5, 130.0, 130.7, 131.2, 132.9, 140.6, 143.0, 143.2, 148.0, 164.5, 165.1, 165.8, 171.4; ESI-HRMS found *m/z* 561.2358 $[M-H]^-$, $C_{30}H_{33}N_4O_7$ requires 561.2355.

NH_2-[*O*-*i*Pr-(3-HABA)]-[*O*-*iLeu*-(3-HABA)]-[*O*-*i*Pr-(3-HABA)]-Gly-CO_2H 15

d$_H$ (500 MHz, DMSO-d$_6$) 0.97 (3H, t, *J* = 7.5, 2-H?), 1.31-1.36 (15H, m, 1-Hß, 2-CH$_a$(CH$_3$) + 3-Ha), 1.67-1.81 (2H, m, 2-Hß + 2-Hß'), 3.93 (2H, d, *J* = 5.6, 4-Ha), 4.60 (1H, m, 2-Ha), 4.72 (2H, sept, *J* = 6.0, 1-Ha, 3-Ha), 6.74 (1H, d, *J* = 8.1, 1-H5), 7.32-7.35 (2H, m, 1-H2 + 1-H6), 7.52-7.59 (4H, m, 2-H2, 2-H6, 3-H2 + 3-H6), 8.11 (1H, d, *J* = 8.3, 2-H5), 8.29 (1H, d, *J* = 8.3, 3-H5), 8.80 (1H, t, *J* = 5.7, 4-NH), 8.95 (1H, s, 2-NH), 9.27 (1H, s, 3-NH); d$_C$ (125MHz, DMSO-d$_6$) 9.4, 18.9, 21.9, 28.6, 41.2, 70.4, 71.4, 76.1, 112.1, 112.6, 113.2, 119.9, 120.0, 120.3, 121.3, 121.6, 129.4, 129.9, 131.2, 132.2, 143.5, 147.2, 147.9, 164.2, 164.4, 165.8, 171.4; ESI-HRMS found *m/z* 643.2738 $[M+Na]^+$, $C_{33}H_{40}N_4NaO_8$ requires 643.2744.

NH_2-[*O*-*i*Pr-(3-HABA)]-[*O*-*p*-^{t}Bu-Bn-(3-HABA)]-[*O*-*i*Pr-(3-HABA)]-Gly-CO_2H 16

d$_H$ (500 MHz, DMSO-d$_6$) 1.30 (9H, s, 2-Ar-C(CH$_3$)), 1.38 (6H, m, 1-Hß, 3-Hß), 3.95 (2H, d, *J* = 6.0, 4-Ha), 4.59 (1H, sept, *J* = 6.0, 1-Ha), 4.74 (1H, sept, *J* = 6.0, 3-Ha), 5.30 (2H, s, 2-Ha), 6.73 (1H, d, *J* = 8.0, 1-H5), 7.36 (1H, d, *J* = 8, 1-H6), 7.40-7.44 (3H, m, 1-H2 + 2-HAr3), 7.51 (2H, d, *J* = 8.0, 2-HAr2), 7.55 (1H, d, *J* = 8.0, 2-H6), 7.61-7.63 (2H, m, 2-H2 + 3-H6), 7.76 (1H, s, 3-H2), 8.11 (1H, d, *J* = 8.2, 2-H5), 8.21 (1H, d, *J* = 8.2, 3-H5), 8.85 (1H, t, *J* = 6.0, 4-NH), 9.14 (1H, s, 2-NH), 9.35 (1H, s, 3-NH); d$_C$ (125MHz, DMSO-d$_6$) 27.1, 27.2, 36.3, 39.5, 46.5, 75.3, 75.7, 76.7, 116.8, 117.9, 118.1, 118.3, 125.1, 125.4, 126.6, 126.6, 126.8, 127.1, 130.4, 132.6, 135.0, 135.3, 136.4, 136.8, 138.9, 148.7, 153.3, 154.0, 155.7, 169.5, 169.8, 171.1, 176.6; ESI-HRMS found *m/z* 733.3189 $[M+Na]^+$, $C_{40}H_{46}N_4NaO_8$ requires 733.3208.

NH_2-[*O*-*i*Pr-(3-HABA)]-[*O*-CH_2-CH_2-indole-(3-HABA)]-[*O*-*i*Pr-(3-HABA)]-Gly-CO_2H 18

LC-MS analysis of this reaction indicated reasonable coupling of the central indole monomer to give the target trimer, however it was not possible to purify and isolate this oligomer. ESI-MS found *m/z* 708 $[M+H]^+$.

NH_2-[*O*-*i*Pr-(3-HABA)]-[*O*-CH_2-COOH-(3-HABA)]-[*O*-*i*Pr-(3-HABA)]-Gly-CO_2H 19

d_H (500 MHz, DMSO-d_6) 1.31 (6H, d, *J* = 6.0, 1-Hß), 1.35 (6H, d, *J* = 6.0, 3-Hß), 3.93 (2H, d, *J* = 5.8, 4-Ha), 4.61 (1H, sept, *J* = 6.0, 1-Ha), 4.71 (1H, sept, *J* = 6.0, 3-Ha), 4.91 (2H, s, 2-Ha), 5.43 (2H, br. s, 1-NH_2), 6.70 (1H, d, *J* = 8.5, 1-H5), 7.42-7.43 (2H, m, 1-H2 + 1-H6), 7.52 (1H, d, *J* = 8.0, 2-H6), 7.58 (1H, s, 2-H2), 7.61-7.66 (2H, m, 3-H2 + 3-H6), 8.06 (1H, d, *J* = 8.5, 2-H5), 8.32 (1H, d, *J* = 8.1, 3-H5), 8.80 (1H, t, *J* = 5.8, 4-NH), 9.28 (1H, s, 3-NH), 9.43 (1H, s, 2-NH); d_C (125MHz, DMSO-d_6) 21.8, 67.2, 70.4, 71.5, 112.6, 112.7, 112.8, 113.5, 119.8, 120.1, 120.7, 121.4, 121.4, 122.0, 129.1, 130.1, 131.1, 132.5, 143.3, 143.5, 147.8, 148.1, 164.0, 164.5, 165.8, 170.7, 171.4; ESI-HRMS found *m/z* 645.2087 $[M+Na]^+$, $C_{31}H_{34}N_4NaO_{10}$ requires 645.2167.

NH_2-[*O*-*i*Pr-(3-HABA)]-[*O*-CH_2-CH_2-OH-(3-HABA)]-[*O*-*i*Pr-(3-HABA)]-Gly-CO_2H 20

d_H (500 MHz, DMSO-d_6) 1.31 (6H, d, *J* = 6.0, 1-Hß) , 1.35 (6H, d, *J* = 6.0, 3-Hß), 3.79 (2H, t, *J* = 4.6, 2-Hß), 3.93 (2H, d, *J* = 5.8, 4-Ha), 4.21 (2H, t, *J* = 4.6, 2-Ha), 4.61 (1H, sept, *J* = 6.0, 1-Ha), 4.71 (1H, sept, *J* = 6.0, 3-Ha), 6.71 (1H, d, *J* = 8.8, 1-H5), 7.37-7.39 (2H, m, 1-H2 + 1-H6), 7.52 (1H, dd, *J* = 8.3, 1.7, 2-H6), 7.58-7.60 (2H, m, 2-H2 + 3-H6), 7.63 (1H, d, *J* = 1.9, 3-H2), 8.08 (1H, d, *J* = 8.3, 2-H5), 8.33 (1H, d, *J* = 8.3, 3-H5), 8.81 (1H, t, *J* = 5.8, 4-NH), 9.20 (1H, s, 2-NH), 9.28 (1H, s, 3-NH); d_C (125MHz, DMSO-d_6) 21.8, 21.9, 41.2, 59.5, 70.4, 71.5, 71.5, 112.1, 112.6, 112.8, 112.9, 119.9, 120.0, 120.5, 121.0, 121.6, 121.7, 129.2, 130.0, 131.2, 143.2, 143.3, 148,0, 164.2, 164.8, 165.8, 171.4; ESI-HRMS found *m/z* 631.2378 $[M+Na]^+$, $C_{31}H_{36}N_4NaO_9$ requires 631.2374.

NH_2-[*O*-*i*Pr-(3-HABA)]-[*O*-CH_2-CH_2-CH_2-S-CH_2-(3-HABA)]-[*O*-*i*Pr-(3-HABA)]-Gly-CO_2H 21

d_H (500 MHz, DMSO-d_6) 1.31 (6H, d, *J* = 6.0, 1-Hß), 1.35 (6H, d, *J* = 6.0, 3-Hß), 2.05 (3H, s, 2-SCH_3), 2.10 (2H, m, 2-Hß), 2.69 (2H, t, *J* = 7.1, 2-H?), 3.93 (2H, d, *J* = 5.8, 4-Ha), 4.24 (2H, t, *J* =5.5, 2-Ha), 4.60 (1H, sept, *J* = 6.0 , 1-Ha), 4.72 (1-H, sept, *J* = 6.0, 3-Ha), 6.71 (1H, d, *J* = 8.0, 1-H5), 7.35-7.42 (2H, m, 1-H2, 1-H6), 7.52 (1H, d, *J* = 8.0, 2-H6), 7.56-7.60 (3H, m, 2-H2, 3-H2, 3-H6), 8.09 (1H, d, *J* = 8.0, 2-H5), 8.20 (1H, d, *J* = 8.0, 3-H5), 8.81 (1H, t, *J* = 5.8, 4-NH), 9.06 (1H, s, 2-NH), 9.30 (1H, s, NH); d_C (125MHz, DMSO-d_6) 14.7. 21.8, 21.9, 28.2, 30.0, 41.2, 67.2, 70.4, 71.5, 110.7, 112.6, 112.8, 112.9, 119.9, 121.0, 121.1, 121.6, 121.7, 129.6, 130.0, 131.2, 131.3, 143.1, 143.3, 148.1, 148.7, 164.2, 164.7, 165.8, 171.4; ESI-HRMS found *m/z* 675.2433 $[M+Na]^+$, C33H40N4NaO8S requires 675.2459.

NH_2-[*O*-*i*Pr-(3-HABA)]-[*O*-CH_2-$CONH_2$-(3-HABA)]-[*O*-*i*Pr-(3-HABA)]-Gly-CO_2H 22

d_H (500 MHz, DMSO-d_6) 1.31 (6H, d, *J* = 6.0, 1-Hß), 1.36 (6H, d, *J* = 6.0, 3-Hß), 3.93 (2H, d, *J* = 5.6, 4-Ha), 4.61-4.71 (4H, m, 1-Ha, 2-Ha + 3-Ha), 6.71 (1H, d, *J* = 8.8, 1-H5), 7.46-7.47 (2H, m, 1-H2 + 1-H6), 7.52 (1H, d, *J* = 8.5, 2-H6), 7.58-7.63 (2H, m, 2-H2 + 3-H6), 7.86 (1H, s, 3-H2), 8.06 (1H, d, *J* = 8.5, 2-H5), 8.11 (1H, d, *J* = 8.5, 3-H5), 8.81 (1H, t, *J* = 5.6, 4-NH), 9.31 (1H, s, 3-NH), 9.73 (1H, s, 2-NH); d_C (125MHz, DMSO-d_6) 21.8, 41.2, 66.5, 68.2, 71.5, 112.7, 119.8, 121.0, 121.1, 122.0, 129.8,

130.1, 130.2, 131.0, 131.1, 132.1, 143.3, 148.2, 148.3, 164.1, 164.9, 165.8, 170.2, 171.5; ESI-HRMS found 622.2517 *m/z* $[M+H]^+$, $C_{31}H_{36}N_5O_9$ requires 622.2508.

NH_2-[*O*-*i*Pr-(3-HABA)]-[*O*-CH_2-CH_2-NH_2-(3-HABA)]-[*O*-*i*Pr-(3-HABA)]-Gly-CO_2H 23

d_H (500 MHz, DMSO-d_6) 1.31 (6H, d, *J* = 6.0, 1-Hß), 1.35 (6H, d, *J* = 6.0, 3-Hß), 3.37 (2H, m, 2-Hß), 3.93 (2H, d, *J* = 5.8, 4-Ha), 4.38 (2H, t, *J* = 4.1, 2-Ha), 4.60 (1H, sept, *J* = 6.0, 1-Ha), 4.72 (1H, sept, *J* = 6.0, 3-Ha), 6.72 (1H, d, *J* = 8.1, 1-H5), 7.39-7.42 (2H, m, 1-H2 + 1-H6), 7.53 (1H, dd, *J* = 8.3, 1.8, 2-H6), 7.59-7.63 (3H, m, 2-H2, 3-H2 + 3-H6), 8.06 (1H, d, *J* = 8.3, 2-H5), 8.29 (1H, d, *J* = 8.9, 3-H5), 8.83 (1H, t, *J* = 5.8, 4-NH), 9.29 (1H, s, 2-NH), 9.32 (1H, s, 3-NH); d_C (125MHz, DMSO-d_6) 20.7, 20.8, 63.9, 69.4, 70.3, 109.6, 111.6, 112.3, 118.8, 119.2, 119.9, 120.0, 120.9, 121.1, 128.3, 129.0, 129.9, 130.2, 142.1, 142.1, 146.6, 147.1, 163.0, 164.3, 164.7, 170.25; ESI-HRMS found *m/z* 608.2701 $[M+H]^+$, $C_{31}H_{38}N_5O_8$ requires 608.2715.

NH_2-[*O*-*i*Pr-(3-HABA)]-[*O*-CH_2-CH_2-CH_2-CH_2-CH_2-NH_2-(3-HABA)]-[*O*-*i*Pr-(3-HABA)]-Gly-CO_2H 24

d_H (500 MHz, DMSO-d_6) 1.31 (6H, d, *J* = 6.0, 1-Hß), 1.35 (6H, d, *J* = 6.0, 3-Hß), 1.51 (2H, m, 2-H?), 1.60 (2H, m, 2-Hd), 1.85 (2H, m, 2-Hß), 2.78 (2H, m, 2-He), 3.93 (2H, d, *J* = 5.5, 4-Ha), 4.17 (2H, t, *J* = 6.5, 2-Ha), 4.60 (1H, sept, *J* = 6.0, 1-Ha), 4.72 (1H, sept, *J* = 6.0, 3-Ha), 5.42 (2H, br. s, 1-NH_2), 6.70 (1H, d, *J* = 8.0, 1-H5), 7.35 (1H, d, *J* = 8.0, 1-H6), 7.38 (1H, s, 1-H2), 7.53 (1H, d, *J* = 8.5, 3-H6), 7.52-7.70 (3H, m, 2-H2, 2-H6 + 3-H2), 8.09 (1H, d, *J* = 8.0, 3-H5), 8.19 (1H, d, *J* = 8.5, 2-H5), 8.81 (1H, t, *J* = 5.5, 4-NH), 9.01 (1H, s, 2-NH), 9.28 (1H, s, 3-NH); d_C (125MHz, DMSO-d_6) 21.8, 21.9, 22.2, 26.5, 27.9, 38.7, 41.2, 68.0, 70.5, 71.4, 110.9, 112.6, 112.9, 113.2, 119.8, 119.9, 121.1, 121.4, 121.7, 129.7, 130.0, 131.1, 131.3, 142.6, 143.5, 148.0, 148.8, 164.2, 164.6, 165.8, 171.4; ESI-HRMS found *m/z* 648.3048 $[M-H]^-$, $C_{34}H_{42}N_5O_8$ requires 648.3039.

NH_2-[*O*-*i*Pr-(3-HABA)]-[*O*-CH_2-*c*Pr-(3-HABA)]-[*O*-*i*Bu-(3-HABA)]-[*O*-*i*Bu-(3-HABA)]-Gly-CO_2H 25

d_H (500 MHz, DMSO-d_6) 0.44 (2H, m, 2-H?), 0.62 (2H, m, 2-H?'), 1.06 (12H, m, 3-H? + 4-H?), 1.31 (6H, d, *J* = 6.0, 1-Hß), 1.34 (1H, m, 2-Hß), 2.16 (2H, m, 3-Hß + 4-Hß), 3.91-3.93 (4H, m, 3-Ha + 4-Ha), 4.04 (2H, d, *J* = 6.8, 2-Ha), 4.63 (1H, sept, *J* = 6.2, 1-Ha), 6.74 (1H, d, *J* = 8.3, 1-H5), 7.37-7.42 (2H, m, 1-H2 + 1-H6), 7.54-7.68 (6H, m, 2-H2, 2-H6, 3-H2, 3-H6, 4-H2 + 4-H6), 8.05 (1H, d, *J* = 8.3, 4-H5), 8.11 (1H, d, *J* = 8.1, 3-H5), 8.26 (1H, d, *J* = 8.1, 2-H5), 8.86 (1H, t, *J* = 5.6, 5-NH), 9.05 (1H, s, 2-NH), 9.44 (1H, s, 4-NH), 9.46 (1H, s, 3-NH); d_C (125MHz, DMSO-d_6) 2.9, 10.0, 19.1, 21.9, 25.1, 27.8, 41.2, 67.0, 70.4, 73.2, 74.5, 74.6, 110.8, 110.9, 111.1, 112.7, 112.8, 119.8, 119.9, 120.2, 120.4, 120.8, 121.4, 122.3, 129.3, 130.1, 130.4, 130.6, 130.7, 131.5, 143.2, 143.4, 148.4, 149.7, 149.8, 164.2, 164.3, 164.5, 171.4; ESI-HRMS found *m/z* 846.3699 $[M+H]^+$, $C_{45}H_{53}N_5NaO_{10}$ requires 846.3685.

NH_2-[*O*-*i*Pr-(3-HABA)]-[*O*-CH_2-*c*Pr-(3-HABA)]-[*O*-CH_2-*c*Pr-(3-HABA)]-[*O*-*i*Bu-(3-HABA)]-Gly-CO_2H 26

d_H (500 MHz, DMSO-d_6) 0.42 (4H, m, 2-H? + 3-H?), 0.58 (4H, m, 2-H?' + 3-H?'), 1.31 (6H, d, *J* = 6.0, 1-Hß), 1.35 (6H, d, *J* = 6.0, 4-Hß), 1.35 (2H, m, 2-Hß + 3-Hß), 3.93 (2H, d, *J* = 4.9, 5-Ha), 4.61 (1H, sept, *J* = 6.0, 1-Ha), 4.72 (1H, sept, *J* = 6.0, 4-Ha), 6.72 (1H, d, *J* = 8.1, 1-H5), 7.36 (1H, dd, *J* = 8.1, 1.8, 1-H6), 7.38 (1H, d, *J* = 1.8, 1-H2), 7.53 (1H, d, *J* = 8.3, H5), 7.53-7.61 (5H, m, 2-H2, 3-H2 + H5), 8.08 (1H, d, *J* = 8.5, 4-H5), 8.12 (1H, d, *J* = 8.3, 3-H5), 8.26 (1H, d, *J* = 8.3, 2-H5), 8.81 (1H, t, *J* = 4.9, 5-NH), 9.02 (1H, s, 2-NH), 9.32 (1H, s, 4-NH), 9.39 (1H, s, 3-NH); d_C (125MHz, DMSO-d_6) 21.9, 21.9, 10.0, 21.8, 21.9, 41.2, 70.5, 71.4, 73.0, 73.2, 111.3, 111.6, 112.6, 112.7, 112.8, 119.9, 120.2, 120.3, 120.9, 121.4, 121.8, 122.0, 129.3, 130.1, 130.5, 130.0, 131.1. 131.6, 143.3, 148.1, 148.4, 149.5, 164.2, 164.3, 164.5, 165.8, 171.4; ESI-HRMS found *m/z* 830.3364 $[M+Na]^+$, $C_{44}H_{49}N_5NaO_{10}$ requires 830.3372.

NH_2-[*O-i*Pr-(3-HABA)]-[*O-i*Bu-(3-HABA)]-[*O-i*Leu-(3-HABA)]-Ile-CO_2H 27

d_H (500 MHz, DMSO-d_6) 0.88 (3H, t, *J* = 7.4, 4-Hd), 0.93-0.97 (6H, m, 3-H? + 4-$CH_ß$(C$\underline{H}_3$)), 1.04 (6H, d, *J* = 6.6, 2-H?), 1.25-1.31 (10H, m, 1-Hß + 3-CH_a(C$\underline{H}_3$) + 4-H?'), 1.53 (1H, m, 4-H?), 1.63-1.79 (2H, m, 3-Hß + 3-Hß'), 1.96 (1H, m, 4-Hß), 2.15 (1H, 2-Hß), 3.95 (2H, d, *J* = 6.6, Ha), 4.35 (1H, t, *J* = 7.7, 4-Ha), 4.53-4.64 (2H, m, 1-Ha + 3-Ha), 6.76 (1H, d, *J* = 8.1, 1-H5), 7.36 (1H, dd, *J* = 8.1, 1.5, 1-H6), 7.39 (1H, d, *J* = 1.5, 1-H2), 7.56-7.61 (4H, m, 2-H2, 2-H6, 3-H2 + 3-H6), 8.09 (1H, d, *J* = 8.1, 3-H5), 8.22 (1H, d, *J* = 8.1, 2-H5), 8.40 (1H, d, *J* = 7.7, 4-NH), 9.03 (1H, s, 2-NH), 9.30 (1H, s, 3-NH); d_C (125MHz, DMSO-d_6) 9.3, 11.0, 15.7, 18.9, 19.0, 21.9, 25.2, 27.8, 28.5, 35.7, 57.2, 70.5, 74.6, 76.0, 110.6, 112.7, 123.0, 113.5, 119.9, 120.2, 120.8, 121.4, 121.5, 121.8, 129.6, 130.3, 131.1, 131.3, 142.2, 143.7, 148.1, 148.7, 158.1, 158.3, 164.2, 164.5, 166.1, 173.2; ESI-HRMS found *m/z* 713.3508 $[M+Na]^+$, $C_{38}H_{50}N_4NaO_8$ requires 713.3521.

NH_2-[*O-i*Pr-(3-HABA)]-[*O-i*Pr-(3-HABA)]-[*O-i*Pr-(3-HABA)]-[*O-i*Pr-(3-HABA)]-[*O-i*Pr-(3-HABA)]-[*O-i*Pr-(3-HABA)]-Gly-CO_2H 28

d_H (500 MHz, DMSO-d_6) 1.34-1.42 (36H, m, 1-6Hß), 3.95 (2H, d, *J* = 5.5, 4-Ha), 4.59-4.76 (6H, m, 1-6Ha), 6.78 (1H, d, *J* = 8.0, 1-H5), 7.34-7.37 (2H, m, 1-H2 + 1-H6), 7.54-7.64 (10H, m, 2-H2, 2H-6, 3-H2, 3-H6, 4-H2, 4-H6, 5-H2, 5-H6, 6-H2, 6-H6), 8.16 (1H, d, *J* = 8.5, H5), 8.24-8.27 (3H, m, H5), 8.34 (1H, d, *J* = 8.5, H5), 8.59 (1H, m, 7-NH), 8.87 (1H, s, 2-NH), 9.16-9.22 (4H, m, 3-NH, 4-NH, 5-NH, 6-NH); d_C unable to obtain meaningful spectrum; ESI-HRMS found *m/z* 1160.4982 $[M+Na]^+$, $C_{62}H_{71}N_7NaO_{14}$ requires 1160.4951; found *m/z* 1182.4777 $[M+2Na-H]^+$, $C_{62}H_{70}N_7Na_2O_{14}$ requires 1182.4771.

Molecular Modelling

Conformational search and Superposition with a gp41 extended helix

A conformational search was performed on hexamer **28**. The structure was minimised by employing a full *Monte Carlo* search in the software Macromodel® using the MMFFs (Merk Molecular Force Fields) method. Water was chosen as implicit solvent and free rotation around the amide bonds was allowed in order to increase the accuracy of the conformational search. The results revealed the lowest energy conformation was the extended structure and all six side chains lie on the same face; a

conformation displaying an alternative arrangement of side-chains however, has a relative potential energy of +3.2 kJ mol^{-1} demonstrating a variety of rotamers are accessible. Using a crystal structure of gp41 (PDB ID: 1AIK) we took a series of superpositions from our hexamer using different combinations of side chains (eg side chains 1,2 + 3 or 5,4 + 3) and the extended helix using different combinations of residues (e.g. *i*, *i* + 3 and *i* + 7 or *i*, *i* + 4 and *i* + 8) and at varying positions on the helix (e.g. towards the *N* or *C* terminus). From the relatively small set we sampled in comparison to the available combinations, we achieved RMSD (Root Mean Square Deviation) values ranging from 0.421-0.788 when superimposing 3 atom pairs consisting of the oxygen of the alkoxy group and the α carbon of the amino acids.

The superposition of the lowest energy conformation of the hexamer using the alkoxy oxygen from rings 2, 3, and 4 with residues at *i*, *i* + 3 and *i* + 7 positions respectively (residues Thr569, Leu566 and Gln562) is shown in Figure S1**a**. This demonstrates side chains from rings 1, 2, 3, 4 and 5 are orientated in a very similar fashion to residues at the *i* - 4, *i*, *i* + 3, *i* + 7 and *i* + 10 positions. Demonstrated by molecular modelling studies and crystal structures (Fig. 3**c**-**e**), the possibility of side chains having different arrangements is thermodynamically viable. With this in mind, the Ar-CO bond on ring 5 has been rotated and Figure S1 **b-d** shows side chain 6 is found to occupy the same space as residues in the *i* + 14, *i* + 15, and *i* + 16 positions. These studies thus show the prospect of the longer oligoamides to mimic extended a-helices.

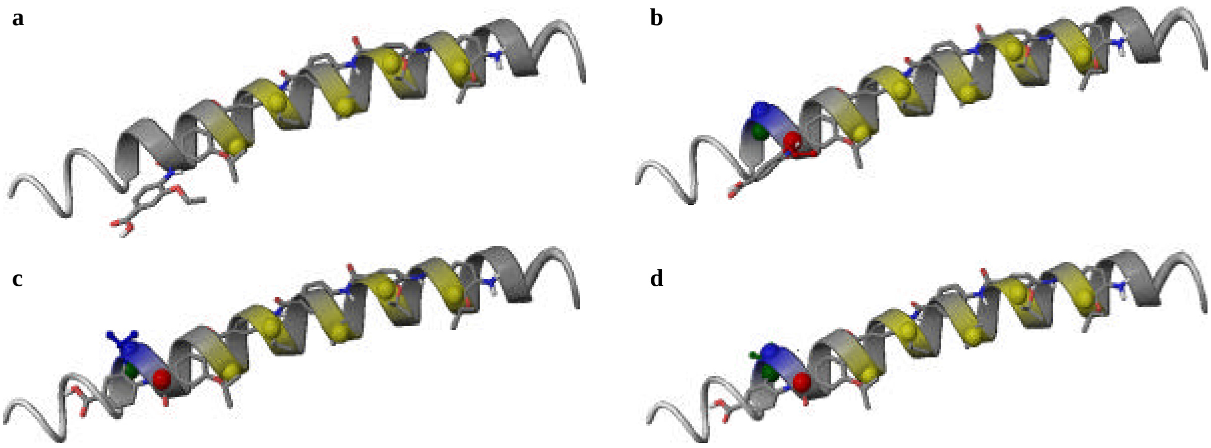

Figure S1. *Figure showing superposition of hexamer with gp41extended helix (PDB ID: 1AIK). a; low energy conformation of hexamer, b-d; demonstrating how rotation of side chain from ring 6 allows mimicry of residues at the i + 14, i + 15, and i + 16 positions.*

Single Crystal X-ray Crystallographic Studies

Crystal Data I Trimer O_2N-[*O*-iPr (3-HABA)]-[*O*-iPr (3-HABA)]-[*O*-iPr (3-HABA)]CO_2Me: This was reported previously for crystals obtained from THF. CCDC 870274 contains the

supplementary crystallographic data for this structure. These data can be obtained free of charge from the Cambridge Crystallographic Data Centre via www.ccdc.cam.ac.uk/data_request/cif.

Crystal Data II Trimer O_2N-[*O*-iPr (3-HABA)]-[*O*-iPr (3-HABA)]-[*O*-iPr (3-HABA)]CO_2Me: Prismatic crystals were obtained by the slow evaporation of a solution of the compound in chloroform/ cyclohexane. A crystal of size 0.14 x 0.03 x 0.01 mm was used for data collection; *?* range = 3.03≤ θ ≤ 22.50°, Crystals belong to Monoclinic; Space group P 21/c; Formula = $C_{31.5}H_{35.5}Cl_{1.5}N_3O_9$; Formula weight = 653.30; *a* = 19.020(4) Å, *b* = 6.9807(14) Å; *c* = 25.837(5) Å, *b* = 101.860(11)°, Volume = 3357.2(12), *Z* = 2, D (calculated): 1. 293 g/cm^3, μ = 0.209 mm^{-1}, Reflections collected 16094; Independent reflections 4244; Observed reflections 1775 [$I > 2\sigma(I)$]; *R* value = 0.1122, wR_2 = 0.2564. Measurements were carried out at 150 K on a Bruker-Nonius Apex X8 diffractometer equipped with an Apex II CCD detector and using graphite monochromated Mo-Ka radiation from a FR591 rotating anode generator. The structure was solved by direct methods using SHELXLS-97 and refined using SHELXL-97. The compound crystallises in the monoclinic space group P21/c with one molecule and half of a $CHCl_3$ in the asymmetric unit. Within the unit cell, each full $CHCl_3$ molecule is disordered across two symmetry generated positions. All non-hydrogen atoms were refined anisotropically. Hydrogen atoms were placed in calculated positions and refined using a riding model. All Uiso(H) values were constrained to be 1.2 times (1.5 for methyl) Ueq of the parent atom. C-Cl bond lengths were restrained to be chemically reasonable. Crystals were very small and did not diffract to high angles hence the data was cut at 2θ = 45°. The data collected was weak leading to high residual R-factors. CCDC 906042 contains the supplementary crystallographic data for this paper. These data can be obtained free of charge from the Cambridge Crystallographic Data Centre via www.ccdc.cam.ac.uk/data_request/cif.

Crystal Data III Trimer O_2N-[*O*-iPr (3-HABA)]-[*O*-iPr (3-HABA)]-[*O*-iPr (3-HABA)]CO_2Me: Prismatic crystals were obtained by the slow evaporation of a solution of the compound in chloroform/ cyclohexane. A crystal of size 0.21 x 0.15 x 0.11 mm was used for data collection; *?* range = 2.42≤ θ ≤ 28.48°, Crystals belong to Triclinic; Space group P-1; Formula = $C_{32}H_{38}N_3O_{9.5}$; Formula weight = 616.65; *a* = 6.4288(6) Å, *b* = 12.3084(15) Å; *c* = 21.217(2) Å, *a* = 95.839(4)°, *b* = 96.993(4)°, *g* = 95.523(11)°, Volume = 1647.7(3), *Z* = 2, D (calculated): 1. 243 g/cm^3, μ = 0.092 mm^{-1}, Reflections collected 31327; Independent reflections 7370; Observed reflections 4764 [$I > 2\sigma(I)$]; *R* value = 0.0737, wR_2 = 0.2110. Measurements were carried out at 150 K on a Bruker-Nonius Apex X8 diffractometer equipped with an Apex II CCD detector and using graphite monochromated Mo-Ka radiation from a FR591 rotating anode generator. The structure was solved by direct methods using SHELXLS-97 and refined using SHELXL-97. Compound crystallises in the triclinic space group *P*-1 with one molecule and half of an EtOH in the asymmetric unit. The half EtOH is disordered over two positions in the asymmetric unit and there is further symmetry-imposed disorder across the inversion centre. All non-hydrogen atoms were refined anisotropically.

Hydrogen atoms were placed in calculated positions and refined using a riding model. All Uiso(H) values were constrained to be 1.2 times (1.5 for methyl) Ueq of the parent atom. C-C and C-O bond lengths of the disordered EtOH were restrained to be chemically reasonable and restraints were also employed on their anisotropic displacement parameters. CCDC 906041 contains the supplementary crystallographic data for this paper. These data can be obtained free of charge from the Cambridge Crystallographic Data Centre via www.ccdc.cam.ac.uk/data_request/cif.

LC-MS Data

Oligomer 7

**Acquisition**	Ion Source Type	ESI	Ion Polarity	Positive	Alternating Ion Polarity	off
	Mass Range Mode	Std/Enhanced	Scan Begin	50 m/z	Scan End	1300 m/z
**Evaluation**	Expected Formula 1		Expected Formula 2		Adductions	H,Na
	Expected Mass 1		Expected Mass 2		Neutral Losses	

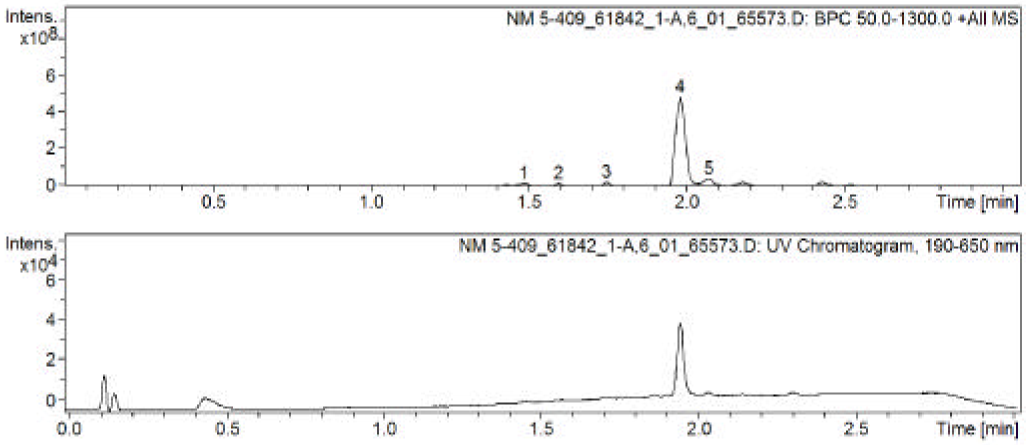

#	Cmpd. Label	RT [min]	Range [min]	Max. m/z	Area	Area %	Area Frac. %
1	Cmpd 1, 1.48 min	1.48	1.45 - 1.53	327.1	25358247	2.4	2.1
2	Cmpd 2, 1.59 min	1.59	1.56 - 1.68	456.2	20744027	2.0	1.7
3	Cmpd 3, 1.74 min	1.74	1.70 - 1.83	430.2	30523664	2.9	2.5
4	Cmpd 4, 1.98 min	1.98	1.92 - 2.03	607.3	1037173932	100.0	86.5
5	Cmpd 5, 2.07 min	2.07	2.03 - 2.12	607.3	84689867	8.2	7.1

Oligomer 8

Acquisition	Ion Source Type	ESI	Ion Polarity	Positive	Alternating Ion Polarity	off
	Mass Range Mode	Std/Enhanced	Scan Begin	50 m/z	Scan End	1300 m/z
Evaluation	Expected Formula 1		Expected Formula 2		Adductions	H,Na
	Expected Mass 1		Expected Mass 2		Neutral Losses	

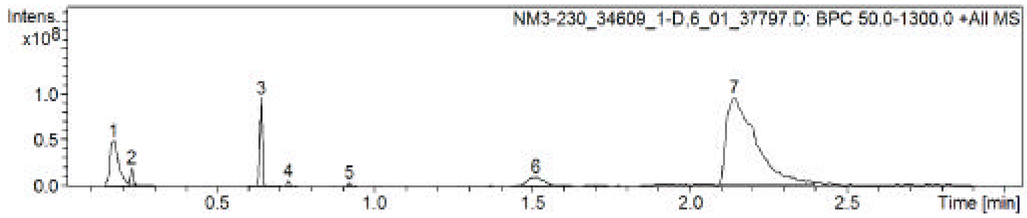

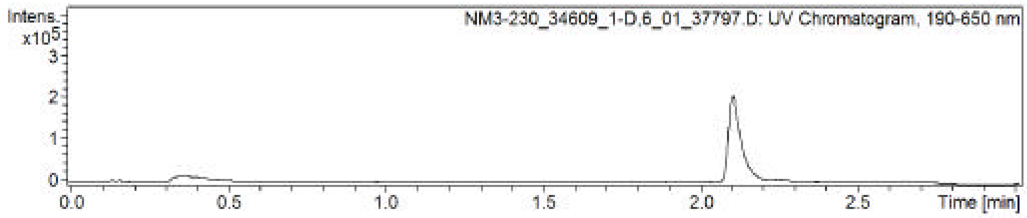

#	Cmpd. Label	RT [min]	Range [min]	Max. m/z	Area	Area %	Area Frac. %
1	Cmpd 1, 0.17 min	0.17	0.14 - 0.22	251.0	96419381	15.3	11.5
2	Cmpd 2, 0.23 min	0.23	0.22 - 0.28	383.5	14295030	2.3	1.7
3	Cmpd 3, 0.64 min	0.64	0.60 - 0.71	94.7	56153086	8.9	6.7
4	Cmpd 4, 0.73 min	0.73	0.72 - 0.76	929.1	3233226	0.5	0.4
5	Cmpd 5, 0.92 min	0.92	0.91 - 0.94	125.8	1866441	0.3	0.2
6	Cmpd 6, 1.51 min	1.51	1.43 - 1.63	420.2	37147460	5.9	4.4
7	Cmpd 7, 2.14 min	2.14	2.09 - 2.40	751.2	630891153	100.0	75.1

Oligomer 9

Acquisition	Ion Source Type	ESI	Ion Polarity	Positive	Alternating Ion Polarity	off
	Mass Range Mode	Std/Enhanced	Scan Begin	50 m/z	Scan End	1300 m/z
Evaluation	Expected Formula 1		Expected Formula 2		Adductions	H,Na
	Expected Mass 1		Expected Mass 2		Neutral Losses	

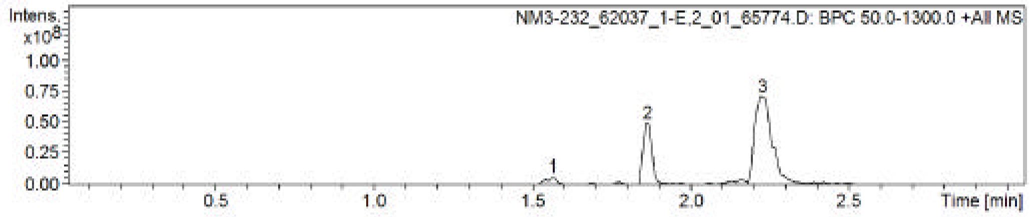

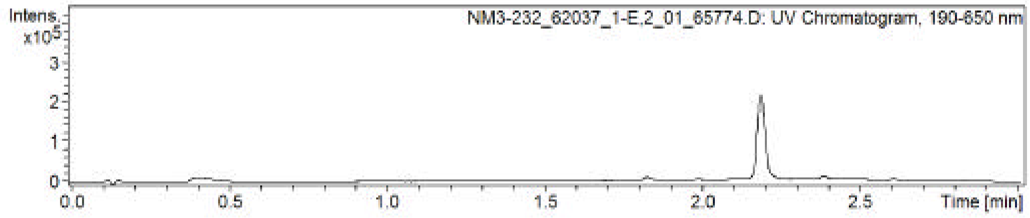

#	Cmpd. Label	RT [min]	Range [min]	Max. m/z	Area	Area %	Area Frac. %
1	Cmpd 1, 1.56 min	1.56	1.51 - 1.63	242.3	14189441	5.1	3.7
2	Cmpd 2, 1.86 min	1.86	1.82 - 1.93	613.2	96583100	35.0	25.0
3	Cmpd 3, 2.22 min	2.22	2.18 - 2.38	753.3	276068411	100.0	71.4

Oligomer 10

**Acquisition**	Ion Source Type	ESI	Ion Polarity	Positive	Alternating Ion Polarity	off
	Mass Range Mode	Std/Enhanced	Scan Begin	50 m/z	Scan End	1300 m/z
**Evaluation**	Expected Formula 1		Expected Formula 2		Adductions	H,Na
	Expected Mass 1		Expected Mass 2		Neutral Losses	

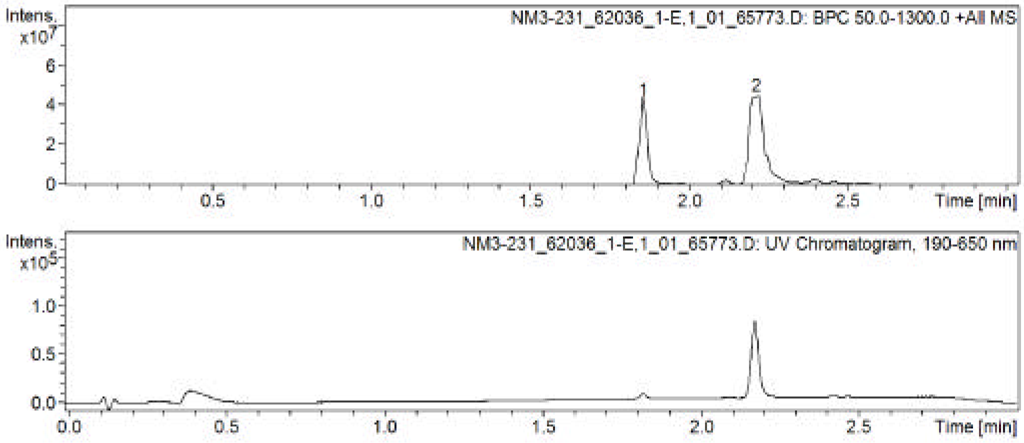

#	Cmpd. Label	RT [min]	Range [min]	Max. m/z	Area	Area %	Area Frac. %
1	Cmpd 1, 1.85 min	1.85	1.81 - 1.95	613.2	76484575	48.5	32.7
2	Cmpd 2, 2.21 min	2.21	2.15 - 2.35	753.3	157634812	100.0	67.3

Oligomer 11

**Acquisition**	Ion Source Type	ESI	Ion Polarity	Positive	Alternating Ion Polarity	off
	Mass Range Mode	Std/Enhanced	Scan Begin	50 m/z	Scan End	1300 m/z
**Evaluation**	Expected Formula 1		Expected Formula 2		Adductions	H,Na
	Expected Mass 1		Expected Mass 2		Neutral Losses	

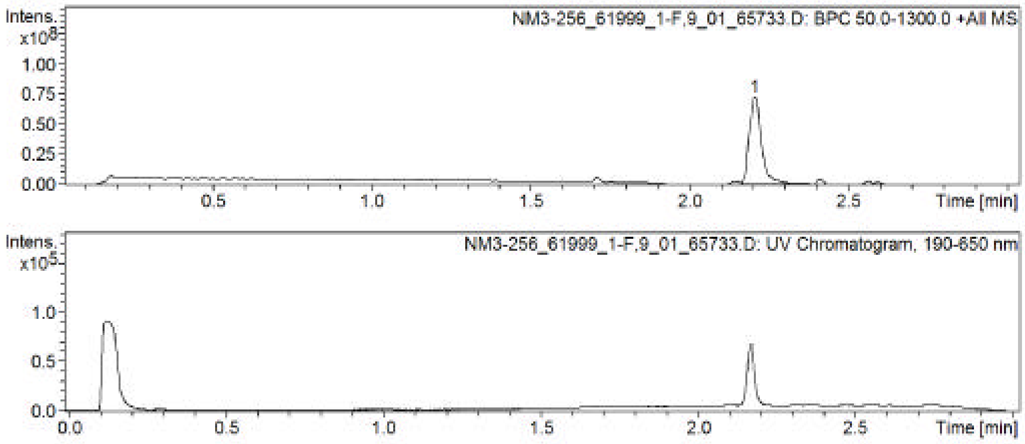

#	Cmpd. Label	RT [min]	Range [min]	Max. m/z	Area	Area %	Area Frac. %
1	Cmpd 1, 2.20 min	2.20	2.16 - 2.35	771.3	203710504	100.0	100.0

Oligomer 12

**Acquisition**	Ion Source Type	ESI	Ion Polarity	Positive	Alternating Ion Polarity	off	
	Mass Range Mode	Std/Enhanced	Scan Begin	50 m/z	Scan End	1300 m/z	
**Evaluation**	Expected Formula 1		Expected Formula 2		Adductions	H,Na	
	Expected Mass 1		Expected Mass 2		Neutral Losses		

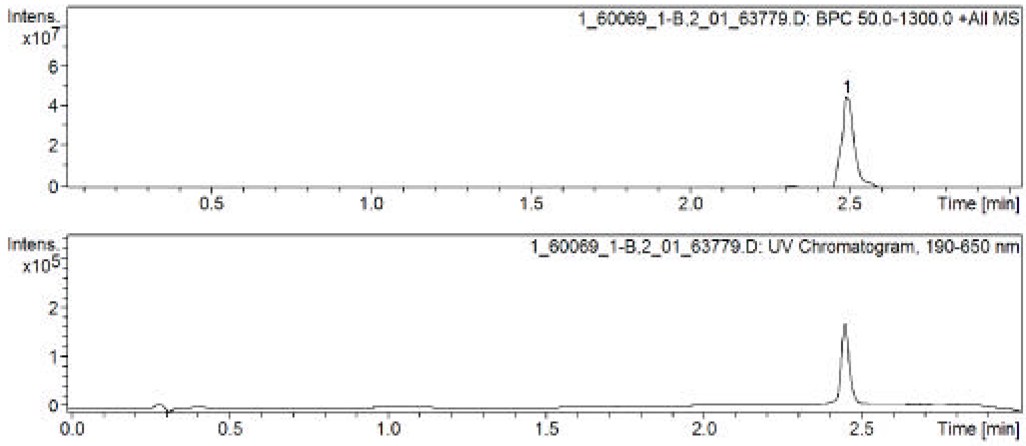

#	Cmpd. Label	RT [min]	Range [min]	Max. m/z	Area	Area %	Area Frac. %
1	Cmpd 1, 2.49 min	2.49	2.42 - 2.64	805.4	122632523	100.0	100.0

Oligomer 13

**Acquisition**	Ion Source Type	ESI	Ion Polarity	Positive	Alternating Ion Polarity	off	
	Mass Range Mode	Std/Enhanced	Scan Begin	50 m/z	Scan End	1300 m/z	
**Evaluation**	Expected Formula 1		Expected Formula 2		Adductions	H,Na	
	Expected Mass 1		Expected Mass 2		Neutral Losses		

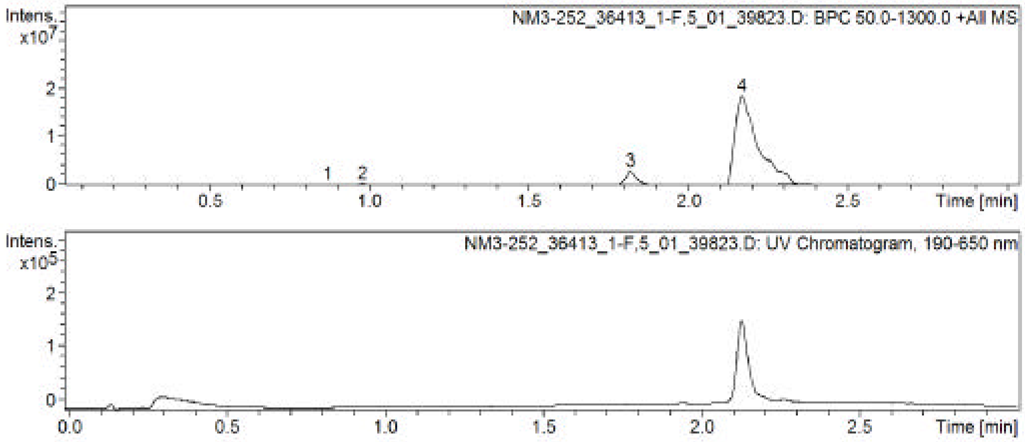

#	Cmpd. Label	RT [min]	Range [min]	Max. m/z	Area	Area %	Area Frac. %
1	Cmpd 1, 0.87 min	0.87	0.85 - 0.92	191.5	120244	0.1	0.1
2	Cmpd 2, 0.98 min	0.98	0.94 - 1.05	722.7	287364	0.3	0.3
3	Cmpd 3, 1.82 min	1.82	1.78 - 1.92	875.7	6474779	7.0	6.5
4	Cmpd 4, 2.17 min	2.17	2.11 - 2.38	737.5	92940069	100.0	93.1

Oligomer 14

**Acquisition**	Ion Source Type	ESI	Ion Polarity	Positive	Alternating Ion Polarity	off	
	Mass Range Mode	Std/Enhanced	Scan Begin	50 m/z	Scan End	1300 m/z	
**Evaluation**	Expected Formula 1		Expected Formula 2		Adductions	H,Na	
	Expected Mass 1		Expected Mass 2		Neutral Losses		

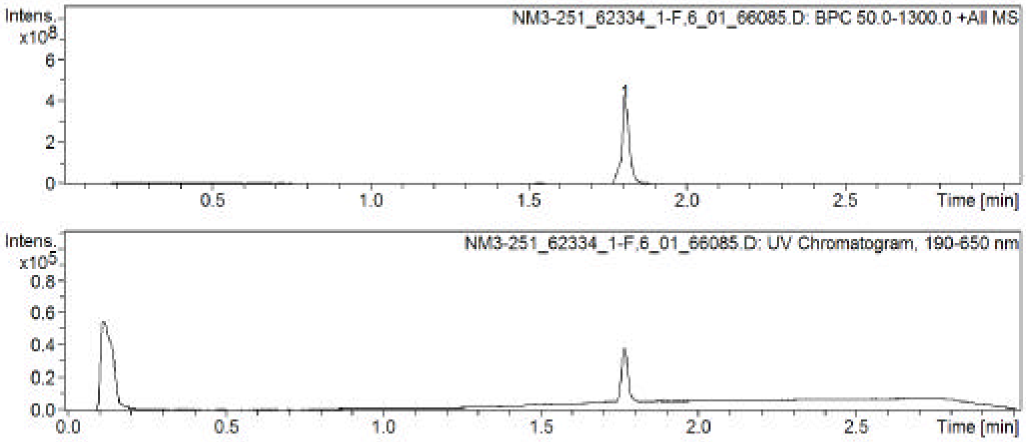

#	Cmpd. Label	RT [min]	Range [min]	Max. m/z	Area	Area %	Area Frac. %
1	Cmpd 1, 1.80 min	1.80	1.77 - 1.99	563.2	709482936	100.0	100.0

Oligomer 15

**Acquisition**	Ion Source Type	ESI	Ion Polarity	Positive	Alternating Ion Polarity	off	
	Mass Range Mode	Std/Enhanced	Scan Begin	50 m/z	Scan End	1300 m/z	
**Evaluation**	Expected Formula 1		Expected Formula 2		Adductions	H,Na	
	Expected Mass 1		Expected Mass 2		Neutral Losses		

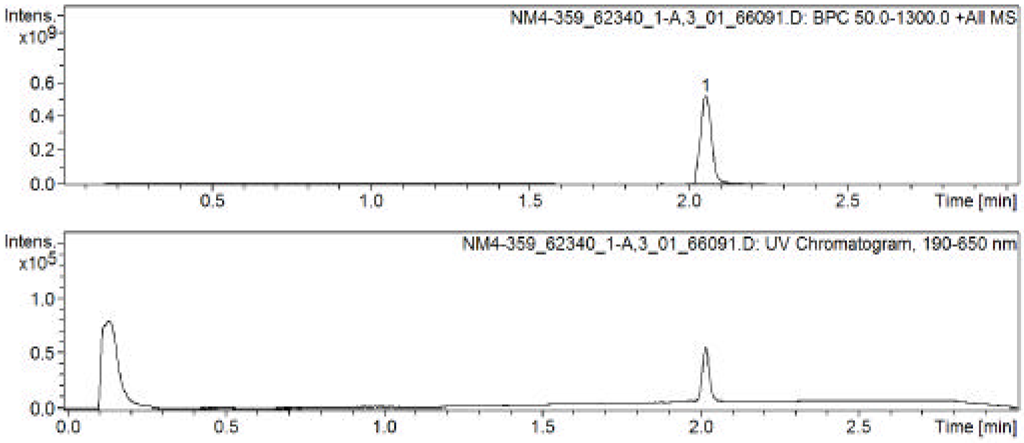

#	Cmpd. Label	RT [min]	Range [min]	Max. m/z	Area	Area %	Area Frac. %
1	Cmpd 1, 2.05 min	2.05	1.99 - 2.24	621.3	1264567769	100.0	100.0

Oligomer 16

**Acquisition**	Ion Source Type	ESI	Ion Polarity	Positive	Alternating Ion Polarity	off
	Mass Range Mode	Std/Enhanced	Scan Begin	50 m/z	Scan End	1300 m/z
**Evaluation**	Expected Formula 1		Expected Formula 2		Adductions	H,Na
	Expected Mass 1		Expected Mass 2		Neutral Losses	

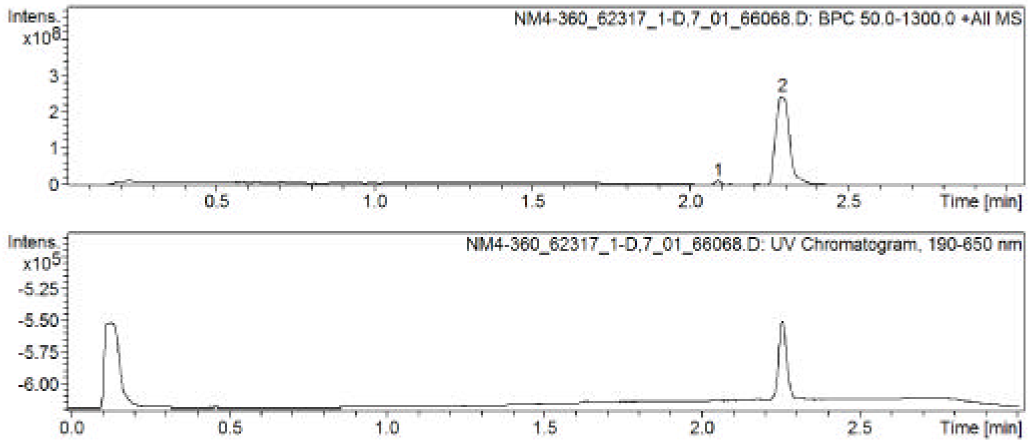

#	Cmpd. Label	RT [min]	Range [min]	Max. m/z	Area	Area %	Area Frac. %
1	Cmpd 1, 2.09 min	2.09	2.06 - 2.17	534.3	20357789	2.7	2.6
2	Cmpd 2, 2.29 min	2.29	2.23 - 2.48	711.4	752915618	100.0	97.4

Oligomer 18 (note: unable to isolate and purify)

**Acquisition**	Ion Source Type	ESI	Ion Polarity	Positive	Alternating Ion Polarity	off
	Mass Range Mode	Std/Enhanced	Scan Begin	50 m/z	Scan End	1300 m/z
**Evaluation**	Expected Formula 1		Expected Formula 2		Adductions	H,Na
	Expected Mass 1		Expected Mass 2		Neutral Losses	

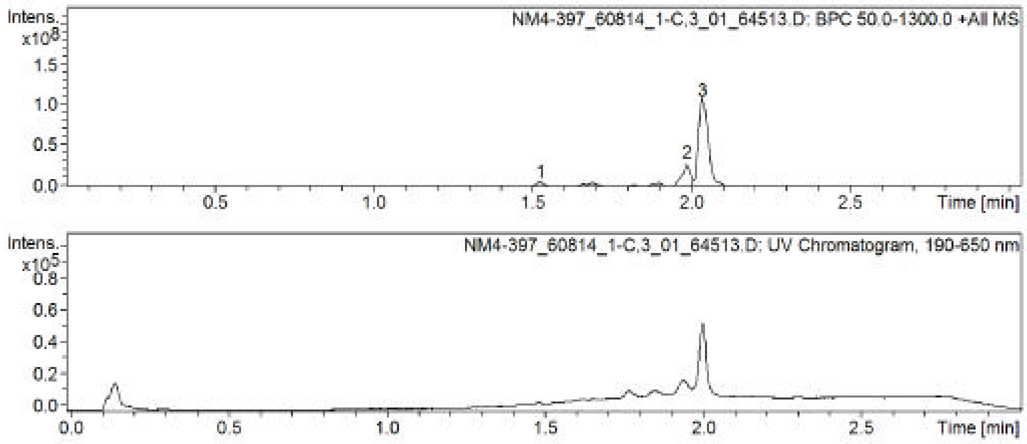

#	Cmpd. Label	RT [min]	Range [min]	Max. m/z	Area	Area %	Area Frac. %
1	Cmpd 1, 1.53 min	1.53	1.49 - 1.55	701.5	8232429	3.5	2.8
2	Cmpd 2, 1.98 min	1.98	1.94 - 2.00	712.3	49312897	21.0	16.9
3	Cmpd 3, 2.03 min	2.03	2.00 - 2.17	708.4	234714603	100.0	80.3

Oligomer 19

**Acquisition**	Ion Source Type	ESI	Ion Polarity	Positive	Alternating Ion Polarity	off
	Mass Range Mode	Std/Enhanced	Scan Begin	50 m/z	Scan End	1300 m/z
**Evaluation**	Expected Formula 1		Expected Formula 2		Adductions	H,Na
	Expected Mass 1		Expected Mass 2		Neutral Losses	

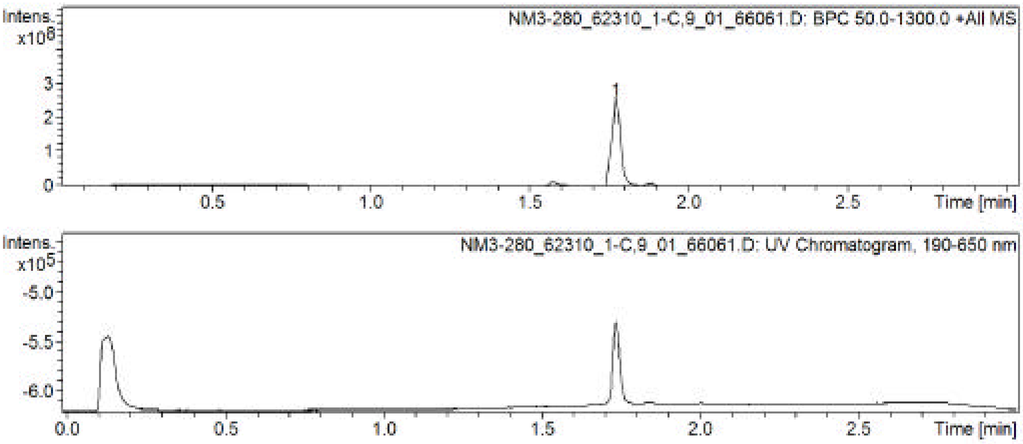

#	Cmpd. Label	RT [min]	Range [min]	Max. m/z	Area	Area %	Area Frac. %
1	Cmpd 1, 1.77 min	1.77	1.73 - 1.85	623.2	497265312	100.0	100.0

Oligomer 20

**Acquisition**	Ion Source Type	ESI	Ion Polarity	Positive	Alternating Ion Polarity	off
	Mass Range Mode	Std/Enhanced	Scan Begin	50 m/z	Scan End	1300 m/z
**Evaluation**	Expected Formula 1		Expected Formula 2		Adductions	H,Na
	Expected Mass 1		Expected Mass 2		Neutral Losses	

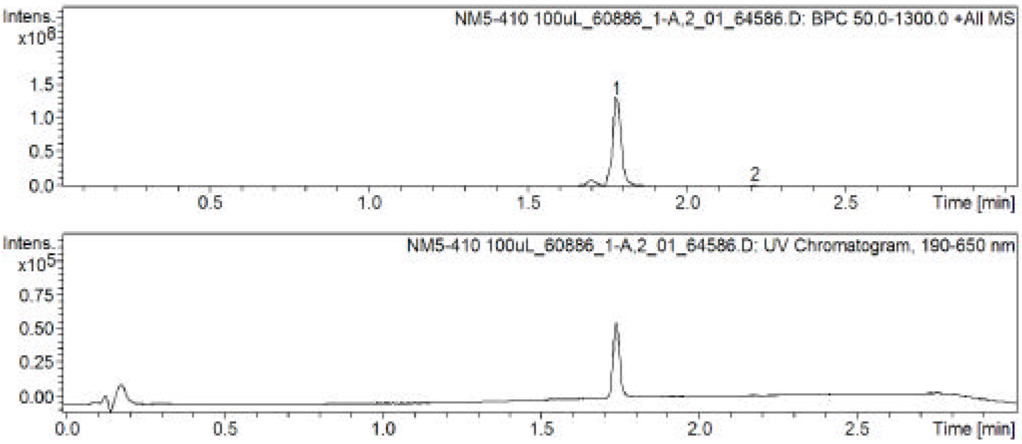

#	Cmpd. Label	RT [min]	Range [min]	Max. m/z	Area	Area %	Area Frac. %
1	Cmpd 1, 1.78 min	1.78	1.74 - 1.88	609.3	251743413	100.0	99.3
2	Cmpd 2, 2.21 min	2.21	2.18 - 2.25	707.8	1716700	0.7	0.7

Oligomer 21

**Acquisition**	Ion Source Type	ESI	Ion Polarity	Positive	Alternating Ion Polarity	off
	Mass Range Mode	Std/Enhanced	Scan Begin	50 m/z	Scan End	1300 m/z
**Evaluation**	Expected Formula 1		Expected Formula 2		Adductions	H,Na
	Expected Mass 1		Expected Mass 2		Neutral Losses	

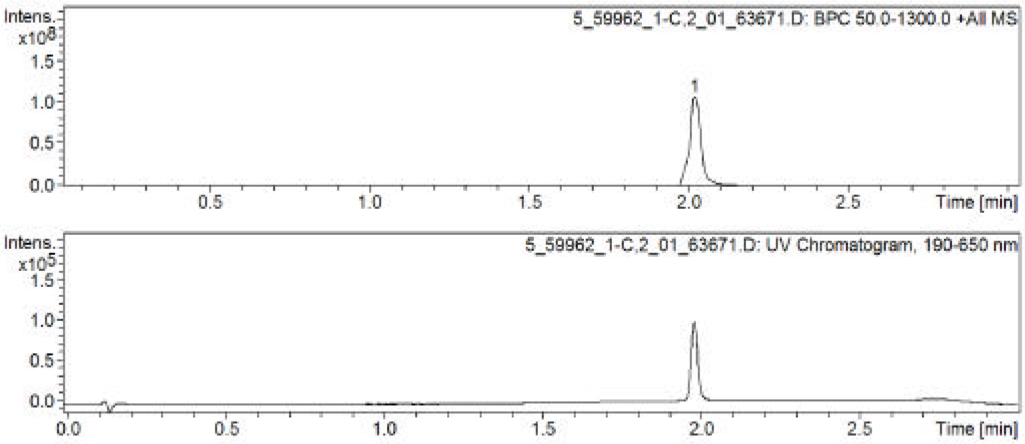

#	Cmpd. Label	RT [min]	Range [min]	Max. m/z	Area	Area %	Area Frac. %
1	Cmpd 1, 2.02 min	2.02	1.96 - 2.27	653.3	278679219	100.0	100.0

Oligomer 22

**Acquisition**	Ion Source Type	ESI	Ion Polarity	Positive	Alternating Ion Polarity	off
	Mass Range Mode	Std/Enhanced	Scan Begin	50 m/z	Scan End	1300 m/z
**Evaluation**	Expected Formula 1		Expected Formula 2		Adductions	H,Na
	Expected Mass 1		Expected Mass 2		Neutral Losses	

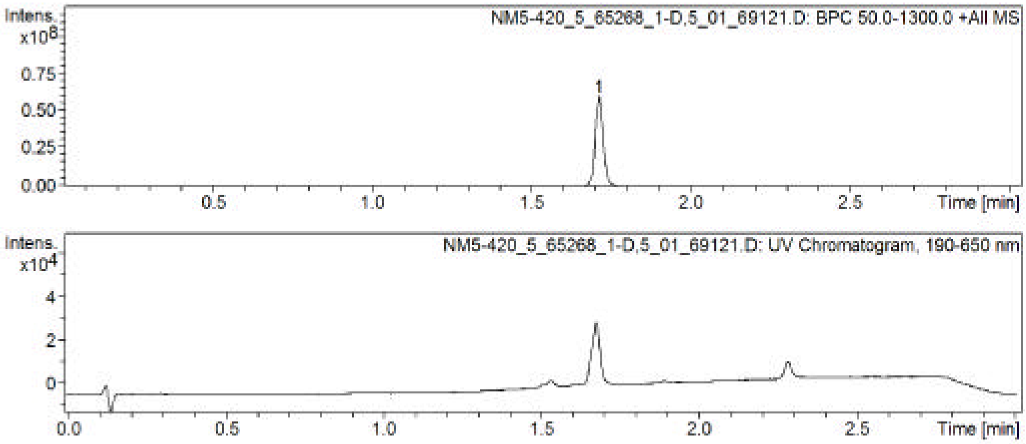

#	Cmpd. Label	RT [min]	Range [min]	Max. m/z	Area	Area %	Area Frac. %
1	Cmpd 1, 1.71 min	1.71	1.63 - 1.82	622.3	101801008	100.0	100.0

Oligomer 23

**Acquisition**	Ion Source Type	ESI	Ion Polarity	Positive	Alternating Ion Polarity	off
	Mass Range Mode	Std/Enhanced	Scan Begin	50 m/z	Scan End	1300 m/z
**Evaluation**	Expected Formula 1		Expected Formula 2		Adductions	H,Na
	Expected Mass 1		Expected Mass 2		Neutral Losses	

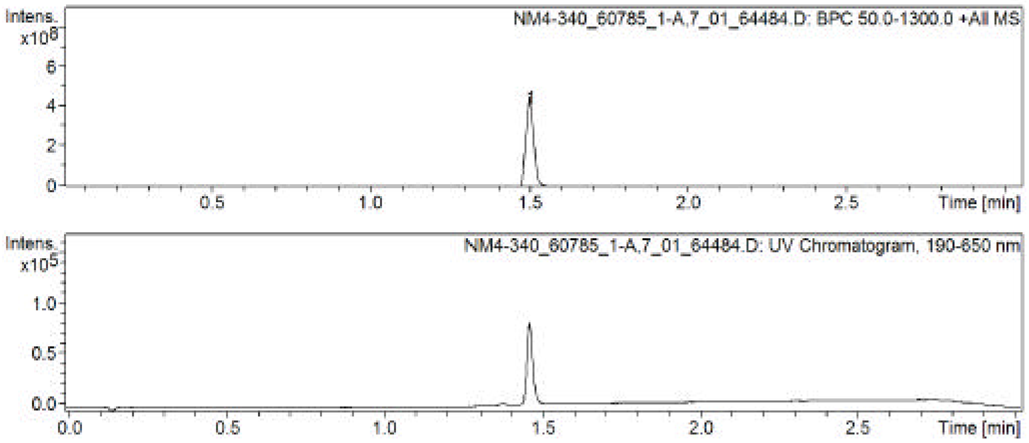

#	Cmpd. Label	RT [min]	Range [min]	Max. m/z	Area	Area %	Area Frac. %
1	Cmpd 1, 1.50 min	1.50	1.46 - 1.59	608.3	744429582	100.0	100.0

Oligomer 24

**Acquisition**	Ion Source Type	ESI	Ion Polarity	Positive	Alternating Ion Polarity	off
	Mass Range Mode	Std/Enhanced	Scan Begin	50 m/z	Scan End	1300 m/z
**Evaluation**	Expected Formula 1		Expected Formula 2		Adductions	H,Na
	Expected Mass 1		Expected Mass 2		Neutral Losses	

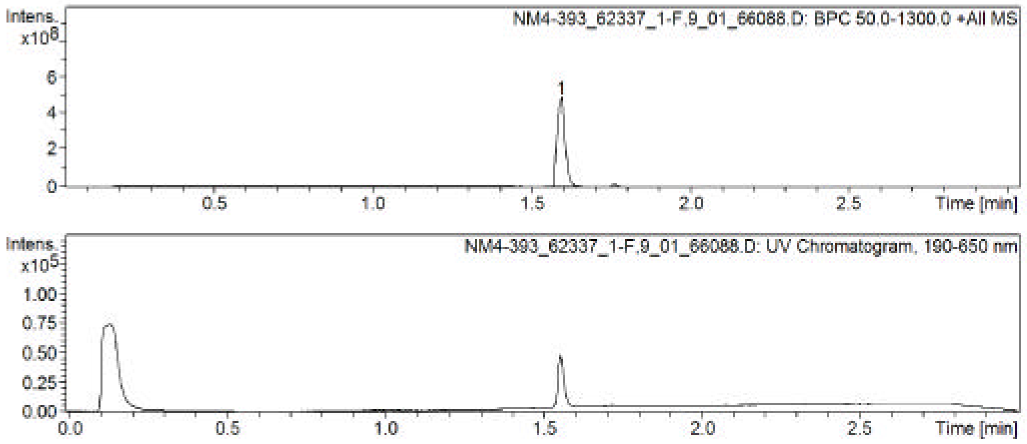

#	Cmpd. Label	RT [min]	Range [min]	Max. m/z	Area	Area %	Area Frac. %
1	Cmpd 1, 1.59 min	1.59	1.54 - 1.66	650.3	843373401	100.0	100.0

Oligomer 25

Acquisition	Ion Source Type	ESI	Ion Polarity	Positive	Alternating Ion Polarity	off
	Mass Range Mode	Std/Enhanced	Scan Begin	50 m/z	Scan End	1300 m/z
**Evaluation**	Expected Formula 1		Expected Formula 2		Adductions	H,Na
	Expected Mass 1		Expected Mass 2		Neutral Losses	

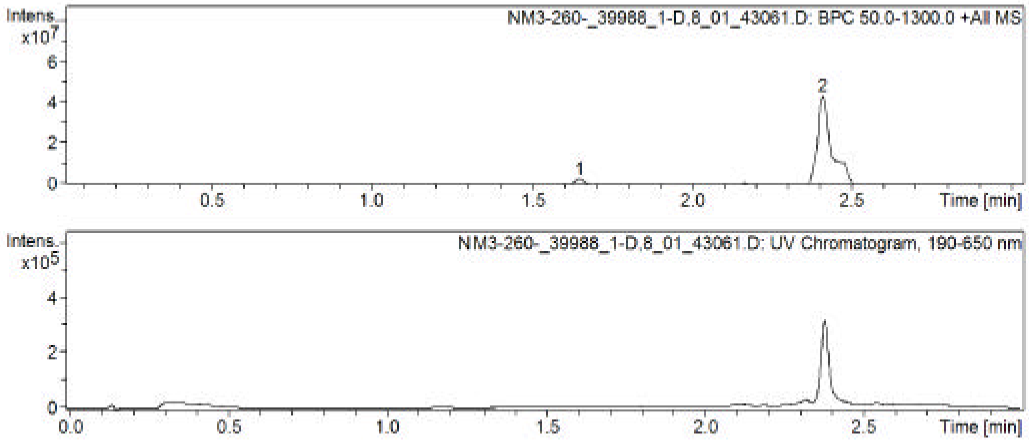

#	Cmpd. Label	RT [min]	Range [min]	Max. m/z	Area	Area %	Area Frac. %
1	Cmpd 1, 1.65 min	1.65	1.62 - 1.71	728.5	4958405	3.7	3.6
2	Cmpd 2, 2.41 min	2.41	2.35 - 2.53	824.8	133264320	100.0	96.4

Oligomer 26

Acquisition	Ion Source Type	ESI	Ion Polarity	Positive	Alternating Ion Polarity	off
	Mass Range Mode	Std/Enhanced	Scan Begin	50 m/z	Scan End	1300 m/z
**Evaluation**	Expected Formula 1		Expected Formula 2		Adductions	H,Na
	Expected Mass 1		Expected Mass 2		Neutral Losses	

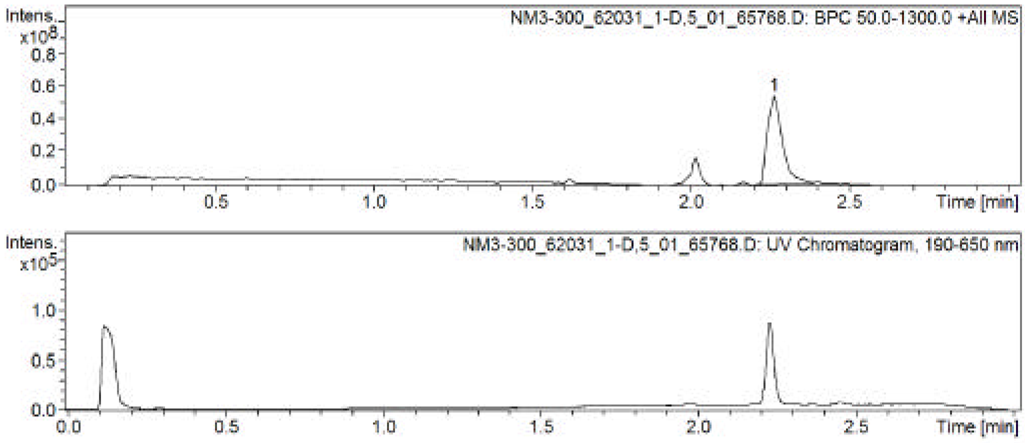

#	Cmpd. Label	RT [min]	Range [min]	Max. m/z	Area	Area %	Area Frac. %
1	Cmpd 1, 2.26 min	2.26	2.21 - 2.38	808.5	171368597	100.0	100.0

Oligomer 27

**Acquisition**	Ion Source Type	ESI	Ion Polarity	Positive	Alternating Ion Polarity	off	
	Mass Range Mode	Std/Enhanced	Scan Begin	50 m/z	Scan End	1300 m/z	
**Evaluation**	Expected Formula 1		Expected Formula 2		Adductions	H,Na	
	Expected Mass 1		Expected Mass 2		Neutral Losses		

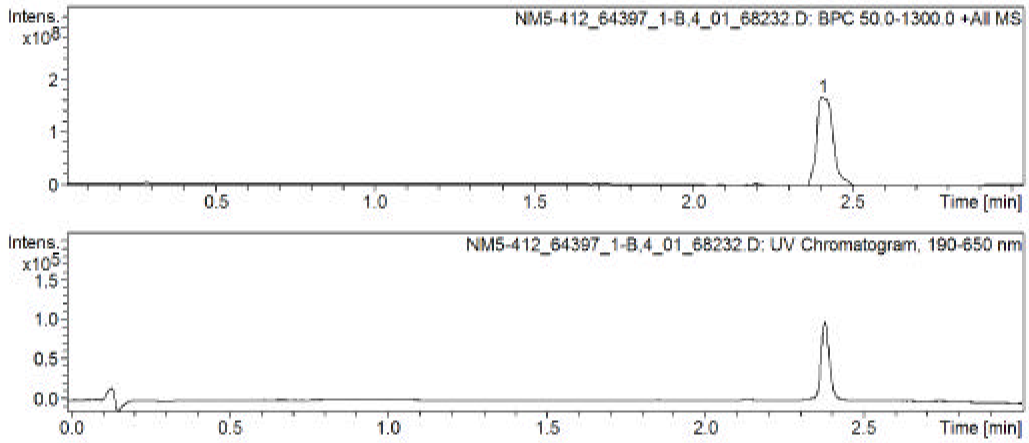

#	Cmpd. Label	RT [min]	Range [min]	Max. m/z	Area	Area %	Area Frac. %
1	Cmpd 1, 2.40 min	2.40	2.35 - 2.55	691.5	593333667	100.0	100.0

References

[1] P. Prabhakaran, V. Azzarito, T. Jacobs, M. J. Hardie, C. A. Kilner, T. A. Edwards, S. L. Warriner, A. J. Wilson, *Tetrahedron* **2012**, *68*, 4485-4491.

[2] J. P. Plante, T. Burnley, B. Malkova, M. E. Webb, S. L. Warriner, T. A. Edwards, A. J. Wilson, *Chem. Commun.* **2009**, 5091-5093.

[3] J. Plante, F. Campbell, B. Malkova, C. Kilner, S. L. Warriner, A. J. Wilson, *Org. Biomol. Chem.* **2008**, *6*, 138-146.
